# Effectiveness and Safety of Chinese Herbal Injections Combined with SOX Chemotherapy Regimens for Advanced Gastric Cancer: a Bayesian Network Meta-Analysis

**DOI:** 10.7150/jca.91301

**Published:** 2024-01-01

**Authors:** Zhi-jun Bu, Shu-run Wan, Peter Steinmann, Ze-tao Yin, Jin-ping Tan, Wen-xin Li, Zhen-yan Tang, Shuo Jiang, Meng-meng Ye, Jin-yang Xu, You-you Zheng, Xue-hui Wang, Jian-ping Liu, Zhao-lan Liu

**Affiliations:** 1 Centre for Evidence-Based Chinese Medicine, Beijing University of Chinese Medicine, Beijing, China.; 2 Dongzhimen Hospital, Beijing University of Chinese Medicine, Beijing Key Laboratory, Beijing, China.; 3 Swiss Tropical and Public Health Institute, Allschwil, Switzerland.; 4 University of Basel, Basel, Switzerland.; 5 Critical Respiratory Medicine, China-Japan Friendship Hospital, Beijing University of Chinese Medicine, Beijing, China.; 6 School of Pharmacy, Hubei University of Chinese Medicine, Wuhan, China.; 7 The First Affiliated Hospital of Nanchang University, Nanchang, China.; 8 School of the First Clinical College, Hubei University of Chinese Medicine, Wuhan, China.; 9 School of Basic Medicine, Hubei University of Chinese Medicine, Wuhan, China.

**Keywords:** Chinese herbal injections, SOX chemotherapy, Advanced gastric cancer, Bayesian network meta-analysis

## Abstract

**Background:** Randomized controlled trials (RCTs) have demonstrated that combining Chinese herbal injections (CHIs) with oxaliplatin plus tegafur (SOX) chemotherapy regimens improves clinical effectiveness and reduces adverse reactions in patients with advanced gastric cancer (AGC). These RCTs highlight the potential applications of CHIs and their impact on AGC patient prognosis. However, there is insufficient comparative evidence on the clinical effectiveness and safety of different CHIs when combined with SOX. Therefore, we performed a network meta-analysis to rank the clinical effectiveness and safety of different CHIs when combined with SOX chemotherapy regimens. This study aimed to provide evidence for selecting appropriate CHIs in the treatment of patients with AGC.

**Methods:** We searched eight databases from their inception until March 2023. Surface Under the Cumulative Ranking Curve (SUCRA) probability values were used to rank the treatment measures, and the Confidence in Network Meta-Analysis (CINeMA) software assessed the grading of evidence.

**Results:** A total of 51 RCTs involving 3,703 AGC patients were identified. Huachansu injections + SOX demonstrated the highest clinical effectiveness (SUCRA: 78.17%), significantly reducing the incidence of leukopenia (93.35%), thrombocytopenia (80.19%), and nausea and vomiting (95.15%). Shenfu injections + SOX improved Karnofsky's Performance Status (75.59%) and showed a significant reduction in peripheral neurotoxicity incidence (88.26%). Aidi injections + SOX were most effective in reducing the incidence of liver function damage (75.16%). According to CINeMA, most confidence rating results were classified as “low”.

**Conclusion:** The combination of CHIs and SOX shows promising effects in the treatment of AGC compared to SOX alone. Huachansu and Shenfu injections offer the greatest overall advantage among the CHIs, while Aidi injections are optimal for reducing the incidence of liver damage. However, further rigorous RCTs with larger sample sizes and additional pharmacological studies are necessary to reinforce these findings.

## 1. Introduction

Gastric cancer (GC) is a common malignant tumor of the digestive system, with various risk factors contributing to its development, including Helicobacter pylori infections, low socioeconomic status, dietary factors, family history, and inherited predisposition 1. Globally, GC poses a significant burden, accounting for over 1 million new cases and approximately 769,000 deaths in 2020, making it one of the leading causes of mortality 2. Early symptoms of GC are often nonspecific and can be mistaken for common digestive disorders, resulting in delayed diagnosis and advanced disease stage at presentation 1. Advanced gastric cancer (AGC) patients experience severe abdominal pain, hemorrhage, and melena, and surgical intervention may not be a suitable treatment option. Chemotherapy, such as the SOX regimen, becomes crucial in slowing down disease progression and improving quality of life 3.

The SOX regimen, comprising Oxaliplatin and Tegafur, is widely used as a first-line clinical treatment for AGC. Oxaliplatin targets DNA by forming platinum-DNA cross-links, thereby inhibiting tumor cell proliferation and differentiation 4. Tegafur enhances the antitumor effect by increasing the concentration of 5-fluorouracil (5-FU) monophosphate, a phosphorylated metabolite of 5-FU, in the tumor 5. However, despite the effectiveness of the SOX regimen, it may also lead to significant side effects, including nausea, vomiting, liver function damage, and peripheral neurotoxicity 6. Therefore, exploring alternative treatment options that can complement or enhance the clinical effectiveness and reduce adverse events of conventional therapy is of great importance.

Traditional Chinese medicine (TCM) has a long history of treating AGC, with reported effects including inhibiting the proliferation of AGC cells by promoting their apoptosis and reducing the toxic side effects of chemotherapy 7. Chinese herbal injections (CHIs) are formulated by extracting active ingredients from Chinese medicine using modern science and technology. In contrast to Chinese medicine decoctions, CHIs can directly enter the circulatory system, which improves their effectiveness, onset time, and duration of action 8. Currently, CHIs are commonly used with the SOX regimen in clinical practice in China to improve treatment effectiveness and reduce adverse reactions. While there are a variety of CHIs, there is insufficient evidence regarding their relative effectiveness, safety, and optimal combination with the SOX regimen for treating AGC.

As a novel evidence-based medical statistical analysis method, Network Meta-Analysis (NMA) combines direct and indirect evidence to evaluate multiple treatments in a single analysis, expanding upon the principles of conventional meta-analysis 9, 10. NMA enables the simultaneous evaluation of various interventions, providing valuable information for clinical decision-making even when direct comparisons are not available 11, 12. Additionally, NMA allows us to rank each intervention based on its effectiveness and the probability of being the optimal treatment 13_._ Hence, we performed a NMA to rank the clinical effectiveness and safety of different CHIs when combined with SOX chemotherapy regimens. The objective of this study was to provide evidence for the selection of appropriate CHIs in the treatment of patients with AGC. By doing so, we aimed to improve the clinical effectiveness and reduce the occurrence of adverse events in patients with AGC, thereby enhancing their overall treatment outcomes.

## 2. Methods

This systematic review was reported by the Preferred Reporting Items for Systematic Review (PRISMA) 2020 guidelines 14 and the Cochrane Handbook for Systematic Reviews of Interventions 15. In the **Supplement S1**, we provide the PRISMA checklist. At PROSPERO, we have registered the protocol with registration number CRD42022383478.

### 2.1. Search strategy

We applied the search strategy to eight databases: China National Knowledge Infrastructure, WanFang, Chinese Scientific Journal Database (VIP), SinoMed, PubMed, Cochrane Library, Excerpt Medica Database (Embase), and Web of Science (WOS). Medical Subject Headings (MeSH) and free-text words were combined. **Supplement S2** lists the search strategies for the corresponding databases. Only Chinese and English studies were included.

### 2.2. Eligibility criteria

(1) Study type: Published randomized controlled trials (RCTs).

(2) Study subjects: Patients with a pathologically confirmed diagnosis of AGC and Tumor, Node, Metastasis (TNM) staging of III-IV 16, regardless of age, gender, or race.

(3) Intervention group: Patients with AGC who were treated with the SOX regimen (Oxaliplatin plus Tegafur) combined with CHIs.

(4) Outcomes: The primary outcomes were clinical effectiveness and improvement rate measured with the Karnofsky Performance Status (KPS) score. The clinical effectiveness was based on the World Health Organization (WHO) effectiveness criteria for solid tumors 17 or Response Evaluation Criteria in Solid tumors (RECIST) criteria. The two criteria are consistent for complete response (CR) and partial response (PR) 18. Total clinical effectiveness = ((number of CR + number of PR)/total number of cases). The KPS score is closely related to the patient's health status. By comparing the KPS score before and after treatment, an increase of ≥10 points after treatment was considered an improvement, a decrease of ≥10 points was rated as a deterioration, and a change of <10 points was classified as stable. The improvement rate of KPS was thus defined as the proportion of patients with KPS improvement (≥10 points) among all patients 19. Secondary outcomes included leukopenia, thrombocytopenia, nausea and vomiting, liver function damage, peripheral neurotoxicity, and survival data.

### 2.3. Study selection

Two reviewers independently read the titles, abstracts, and full texts to identify suitable studies and extracted data from eligible studies. A third reviewer checked and verified the database. If the data extracted by the two reviewers were inconsistent, the three reviewers would discuss agreement. The data we extracted are as follows: first author name, year of publication, sample size (number of AGC patients in the intervention group, number of AGC patients in the control group), mean age (mean age of the intervention group, mean age of the control group), treatment mode (intervention group treatment mode, control group treatment mode), drug doses, frequency of drug use, course of treatment, and outcome measures for the intervention and control groups.

### 2.4. Risk of bias and evidence quality assessment

Two reviewers individually evaluated the included studies for bias with the Cochrane 2.0 Bias Risk Tool. After the evaluation, if there was any disagreement, a third reviewer was consulted, and the three reviewers worked together to determine the final bias evaluation result. We assessed the quality of the included studies as follows 20: (a) bias during randomization, (b) bias of expected interventions, (c) bias of missing outcome data, (d) bias of outcome measurement, and (e) bias of selective outcome reporting. Each aspect was assessed on a scale of (a) low risk, (b) some concerns, and (c) high risk.

Following guidance published on the Confidence in Network Meta-Analysis (CINeMA) website (https://cinema.ispm.unibe.ch/), we evaluated the evidence from the included studies. CINeMA considers six aspects: within-study bias, reporting bias, indirectness, imprecision, heterogeneity, and incoherence 21.

### 2.5. Statistical analysis

Analyses were conducted with the R 3.6.1 gemtc package for Bayesian NMA. We used relative risk (RR) and 95% confidence intervals (CI) as measures of binary outcomes. We set the number of pre-iterations and the number of iterations to 20,000 and 50,000, respectively. Based on a trajectory plot, a density plot, and a Brooks-Gelman-Rubin diagnostic plot, we determined whether a satisfactory degree of convergence had been achieved. If RCTs presented excellent homogeneity in article design, intervention details, control details, and outcomes, a random-effects model was used for the analysis. If there was heterogeneity between the study results (I^2^ > 50% or P < 0.1), further subgroup analysis was conducted. In addition, based on the R3.6.1 software, we sequentially completed data processing, a network evidence diagram, heterogeneity analysis, and forest plots. We calculated surface under the cumulative ranking curve (SUCRA) values for each outcome measure and different interventions and used an annular heat map to reflect the ranking of different treatments. STATA 17.0 was used for the detection of publication bias. We employed funnel plots to assess potential publication bias, and the Peters test was used for additional validation. There was no closed loop between intervention and control measures in all included studies.

## 3. Results

### 3.1. Study selection

**Figure 1:**
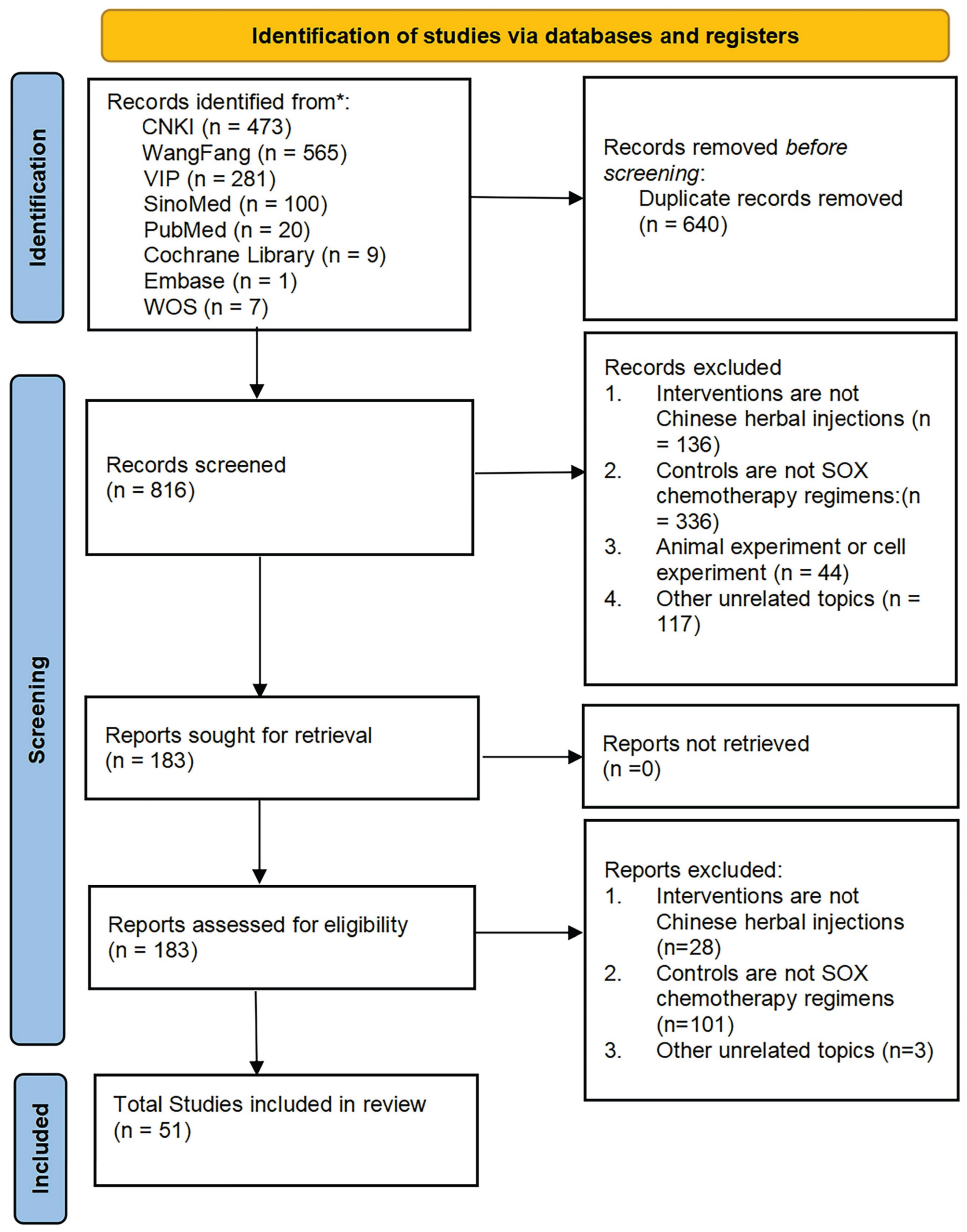
Flowchart of the search for eligible studies. CNKI: China National Knowledge Infrastructure; SinoMed: the Chinese Biomedical Literature Database; WanFang: the WanFang Database; VIP: the Chinese Scientific Journals Full-Text Database; Embase Database: Excerpta Medica Database; WOS Database: Web of Science Database.

Using the search strategy, we identified 1456 studies from eight databases. Among them, 640 duplicates were found using EndNote X9.3.3. After filtering by study title and abstract, another 633 studies were removed. The full text of the 183 retained studies was screened, and 51 studies were identified for inclusion in our review. The flowchart is shown in **Fig. 1**.

### 3.2. Study characteristics

The 51 included studies reported on 3,703 patients with AGC who had a pathological diagnosis of gastric malignancy and were classified as TNM stage III-IV. Of these, 1,858 patients were in the intervention group, and 1,845 were in the control group. A total of 9 different CHIs were used in these 51 studies, including Aidi injections (ADI), Shenfu injections (SFI), Shenqifuzhen injections (SQFZI), Fufangkushen injections (FFKSI), Kangai injections (KAI), Kanglaite injections (KLTI), Huachansu injections (HCSI), Xiaoaiping injections (XAPI), and Huangqi injections (HQI). All CHIs are listed on the website of the China Drug Administration (https://www.nmpa.gov.cn). Furthermore, taxonomic validation of the species composition of all CHIs has been conducted on the following three websites (http://mpns.kew.org/mpns-portal/, http://www.plantsoftheworldonline.org, and https://www.catalogueoflife.org/). In **Supplement S3**, we describe details of the composition, source, indications, and adverse reactions of these CHIs. Among the 51 included studies that were included, a total of 41 assessed clinical effectiveness, while 18 studies assessed the improvement rate of the KPS score. In addition, in terms of adverse events, 26 studies assessed the incidence of leukopenia, 22 studies that of thrombocytopenia, 23 studies mentioned nausea and vomiting, 19 studies investigated liver function damage, and 31 studies peripheral neurotoxicity. An analysis of 51 studies identified a prevalent SOX chemotherapy regimen. This regimen dictates the intravenous administration of 130 mg/m² of Oxaliplatin once a day (qd) on the first day d1 of each 21-day treatment cycle. Tegafur, the regimen's second component, is orally administered. Its dosage is modulated according to the patient's body surface area (BSA).

Specifically, each dosage is 40 mg for patients with a BSA less than 1.25 m², 50 mg for those with a BSA from 1.25 m² to 1.5 m², and 60 mg for those exceeding 1.5 m². Tegafur is taken twice daily (bid), starting from the first day and continuing for 14 days (d1~d14) of each cycle. Each treatment cycle lasts 21 days. It commences with the administration of Oxaliplatin and a 14-day course of Tegafur, followed by a 7-day intermission before the next cycle begins. **Table 1** shows the basic features of the included studies.

### 3.3. Risk of bias in included studies

**Figure 2:**
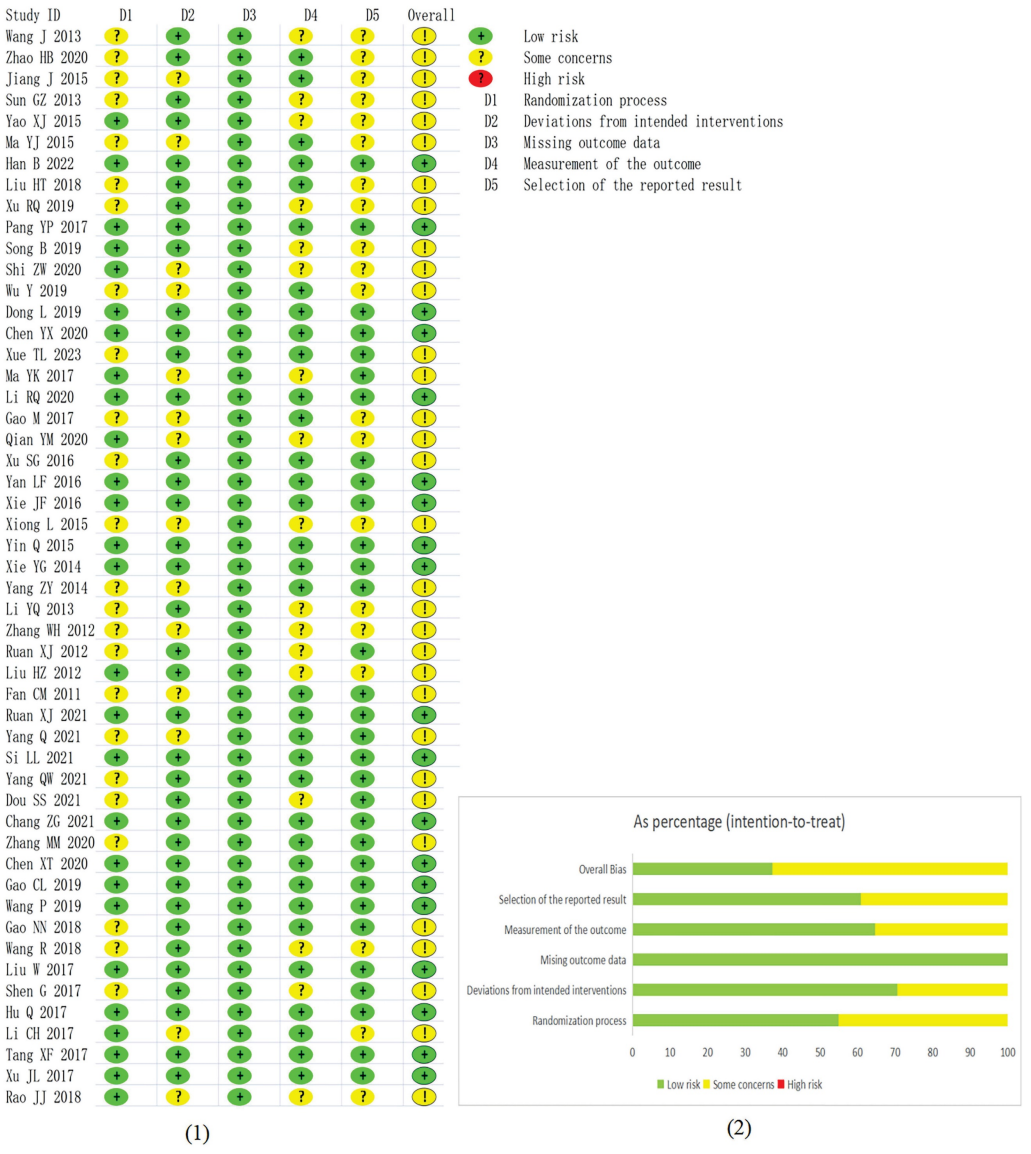
The risk of bias for each study, as well as the overall summary risk of bias. (1) Risk of bias for each included study. (2) overall summary risk of bias.

In terms of bias during randomization, we found 27 studies that precisely described the generation of the random allocation sequences, of which 24 studies employed the random number table method, 1 study used the random drawing method, 1 study used the random envelope method, and 1 study used randomization according to odd and even numbers in the order of enrollment. 24 studies only mentioned randomization without further specifying the method. There was no baseline difference between all 51 studies. None of the 51 studies explicitly stated the use of a blinding method. All studies reported all outcome data. In terms of bias in outcome measures, 34 studies were found appropriate, and 17 were not informative. Data analysis of results was consistent with an analysis plan that had been predetermined before the acquisition of unblinded outcome data in 31 studies. In comparison, 20 studies were unspecific on this point. All said, 19 studies had a low risk of bias in terms of overall quality, while for the remaining 32 studies, there were some concerns about quality. The risk of bias for each study, and the overall summary risk of bias, are displayed in **Fig. 2**.

### 3.4. Pairwise meta-analysis

We performed a pairwise meta-analysis of all interventions for eight outcomes. Forest plots and heterogeneity analyses for pairwise meta-analysis of outcomes are presented in **Supplement S4**. In terms of clinical effectiveness, we found that compared with the SOX regimen exclusively, the addition of CHIs had a RR = 1.37 (95% CI: 1.27 to 1.47, Z = 8.680, P < 0.001), while the improvement rate of the KPS score had a RR = 1.48 (95% CI: 1.32 to 1.65, Z = 6.923, P < 0.001). The incidence of leukopenia had a RR = 0.65 (95% CI: 0.58 to 0.73, Z = -7.211, P < 0.001), the incidence of thrombocytopenia a RR = 0.64 (95%CI: 0.54 to 0.77, Z= -4.913, P < 0.001), the incidence of nausea and vomiting a RR = 0.65 (95%CI: 0.55 to 0.77, Z = -5.214, P < 0.001), liver function damage a RR = 0.56 (95% CI: 0.44 to 0.71, Z = -4.789, P < 0.001), and the incidence of peripheral neurotoxicity a RR = 0.64 (95%CI: 0.56 to 0.74, Z= -6.474, P < 0.001). All pairwise comparisons of CHIs combined with the SOX regimen versus the SOX regimen alone were statistically significant. The results of the heterogeneity analyses show that most combinations of CHIs and SOX regimen were homogeneous (I^2^ < 50% and P > 0.1) except for the incidence of nausea and vomiting (I^2^ = 59.7%, P < 0.001). Specifically, there was significant heterogeneity between the use of ADI combined with SOX chemotherapy and SOX chemotherapy alone (I^2^ = 48.9%). A comparison analysis of the seven studies involved revealed that four studies used antiemetic drugs during treatment, while three studies did not mention the use of antiemetic drugs. Therefore, we speculated that the use of antiemetic drugs during treatment may be the main source of heterogeneity. Subgroup analysis and meta-regression analysis were conducted based on whether antiemetic drugs were used, and the results are presented in **Supplement S5**. Heterogeneity between the group that used antiemetic drugs and the group that did not use antiemetic drugs was significantly reduced after subgroup analysis, and the results of the meta-regression analysis showed that P < 0.05. Therefore, we believe that the use of antiemetic drugs may be the main cause of heterogeneity in the incidence of nausea and vomiting. We conducted leave-one-out sensitivity analyses for all studies and found that the results were robust and reliable (p< 0.05). The forest plot for sensitivity analysis can be seen in **Supplement S6**.

### 3.5. Network meta-analysis

**Figure 3:**
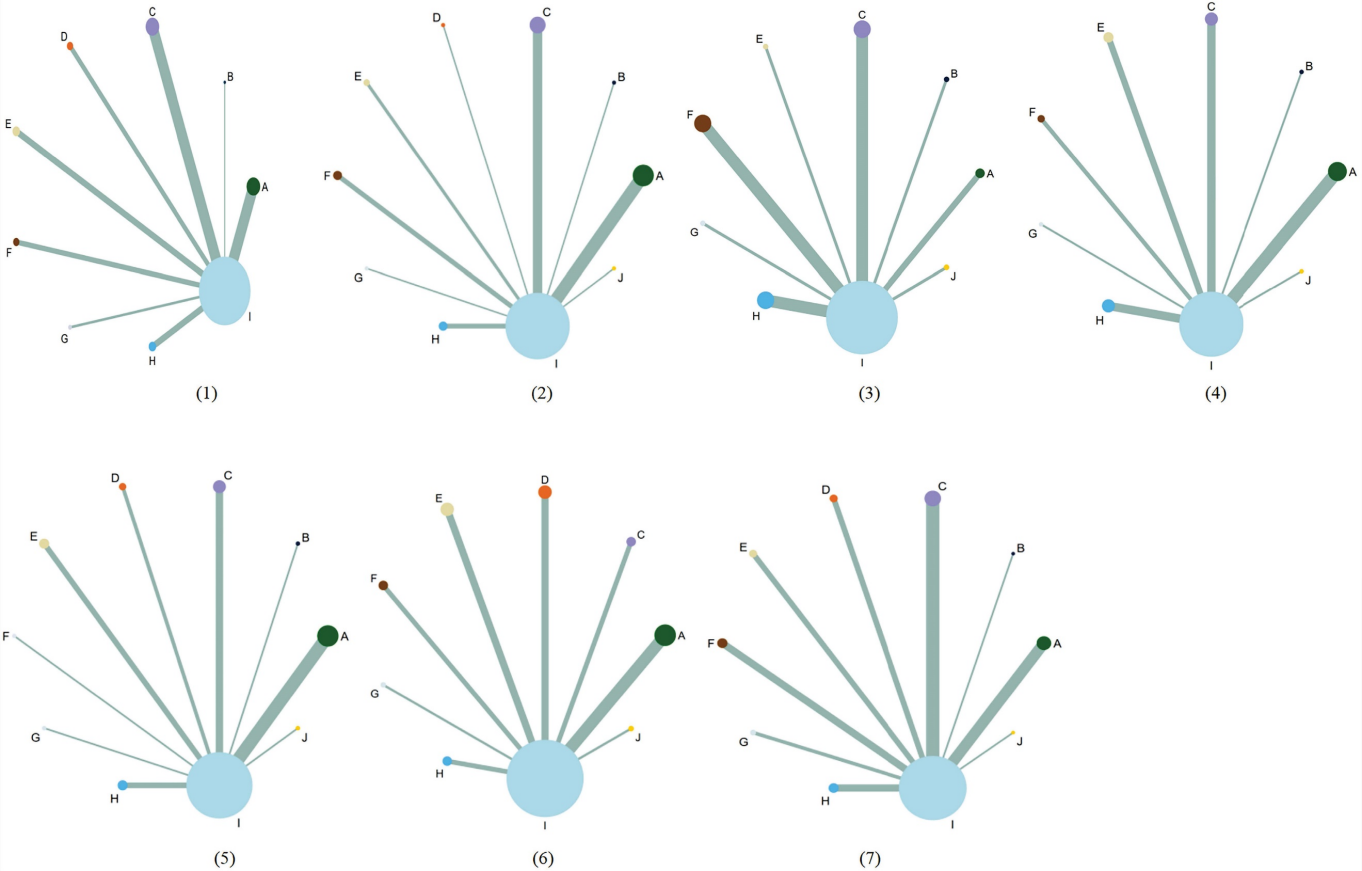
The network structure diagram for outcome measures. Each circle on the diagram represents a treatment, and its size reflects the number of studies evaluating that treatment. The lines connecting the circles indicate direct comparisons between treatments. The figure features seven subfigures, each representing a different outcome: (1) Clinical effectiveness; (2) Improvement rate of KPS score; (3) Incidence of leukopenia; (4) Incidence of thrombocytopenia; (5) Incidence of nausea and vomiting; (6) Incidence of liver function damage; (7) Incidence of peripheral neurotoxicity. Additionally, each specific Chinese herbal injection is identified as follows: ADI, Aidi injections. SFI, Shenfu injections. SQFZI, Shenqifuzheng injections. FFKSI, Fufangkushen injections. KAI, Kangai injections. KLTI, Kanglaitei injections. HCSI, Huachansu injections. XAPI, Xiaoaiping injections. SOX, SOX chemotherapy regimens, HQI, Huangqi injections.

**Figure 4:**
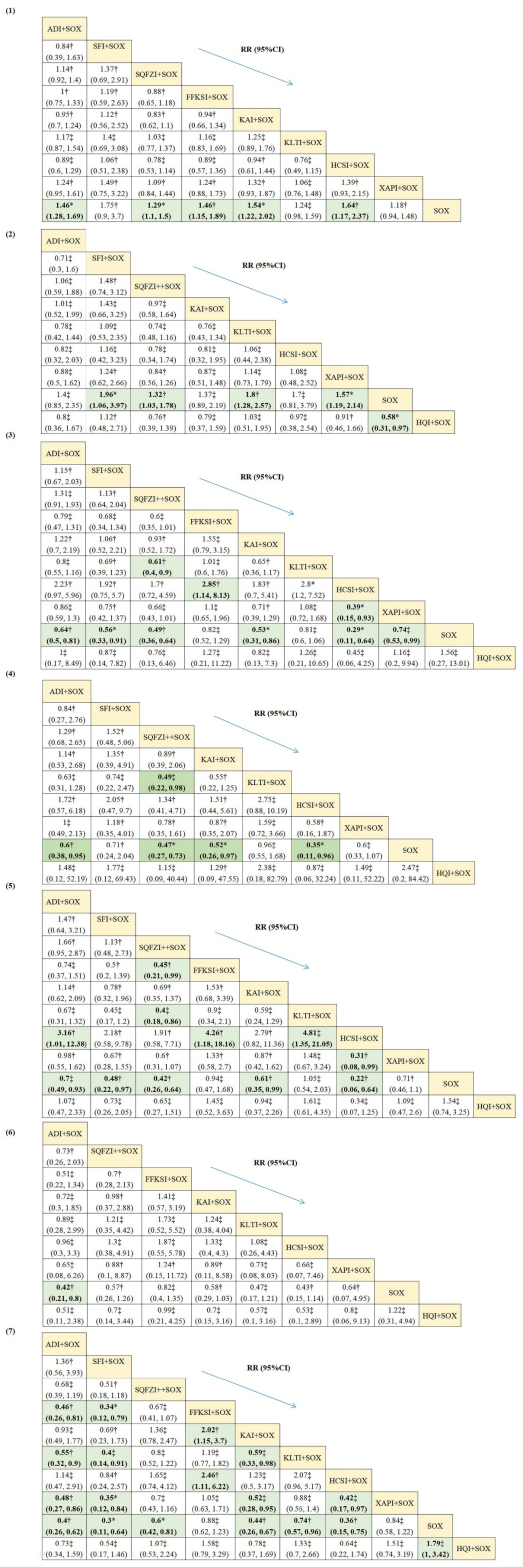
Relative effect analysis of outcomes. Cells filled with yellow in the table represent intervention measures, while cells filled with green indicate statistical significance. (1) Clinical effectiveness; (2) Improvement rate of KPS score; (3) Incidence of leukopenia; (4) Incidence of thrombocytopenia; (5) Incidence of nausea and vomiting; (6) Incidence of liver function damage; (7) Incidence of peripheral neurotoxicity. ADI, Aidi injections. SFI, Shenfu injections. SQFZI, Shenqifuzheng injections. FFKSI, Fufangkushen injections. KAI, Kangai injections. KLTI, Kanglaitei injections. HCSI, Huachansu injections. XAPI, Xiaoaiping injections. SOX, SOX chemotherapy regimens, HQI, Huangqi injections. Statistically significant results were in bold and highlighted. The certainty of the evidence (according to **Supplementary CINeMA results**) was incorporated in this figure. * Moderate quality of evidence. † Low quality of evidence. ‡ Very low quality of evidence.

**Figure 5:**
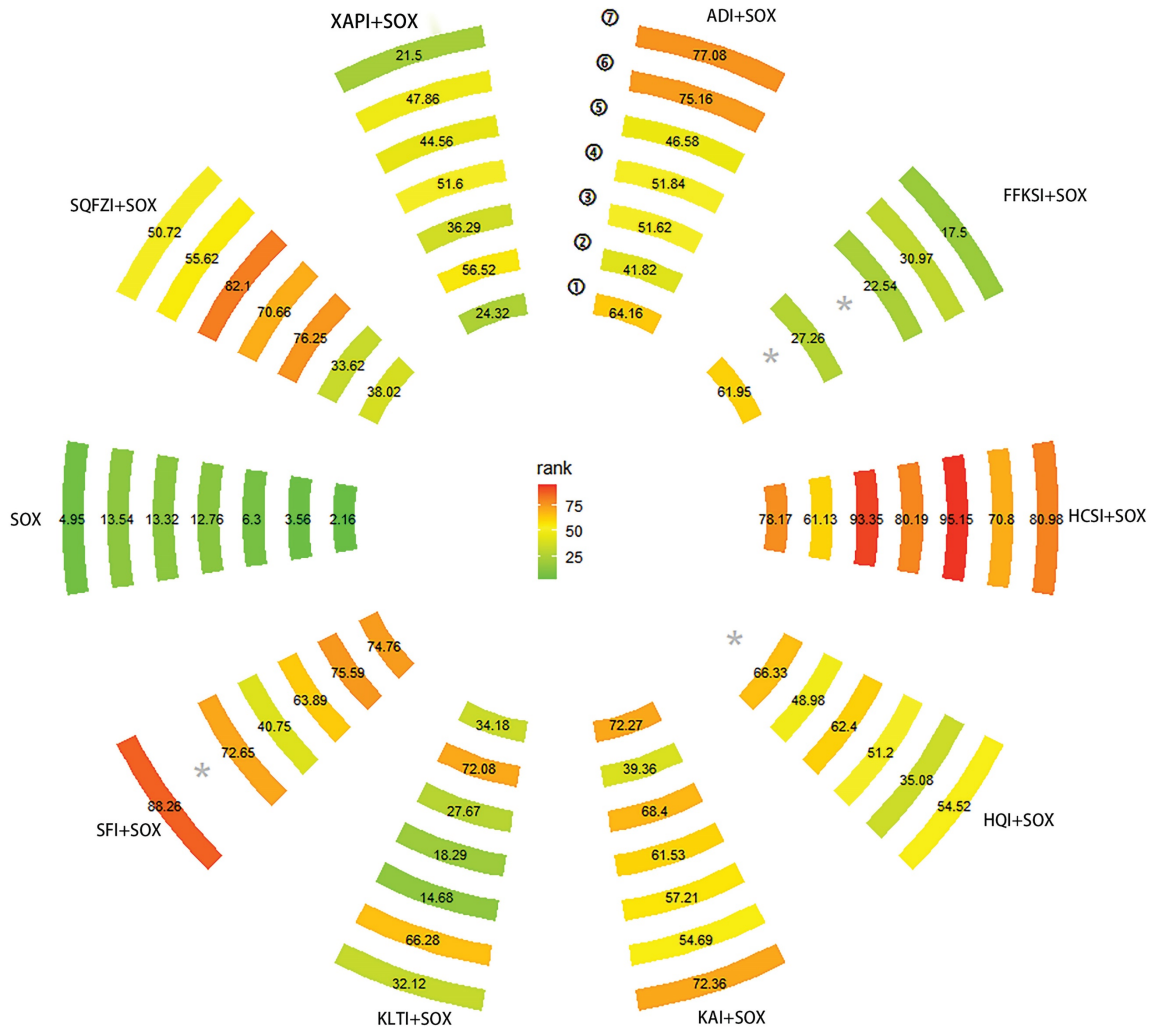
An Annular heat map of SUCRA values based on different outcome measures. It reflects the correlation of the SUCRA values for different interventions with their rankings, with larger numeric results, better rankings, and darker colors. “①”, Clinical effectiveness. “②”, The improvement rate of KPS score. “③”, Incidence of leukopenia. “④”, Incidence of thrombocytopenia. “⑤”, Incidence of nausea and vomiting. “⑥”, Incidence of liver function damage. “⑦”, Incidence of peripheral neurotoxicity. ADI, Aidi injections. SFI, Shenfu injections. SQFZI, Shenqifuzheng injections. FFKSI, Fufangkushen injections. KAI, Kangai injections. KLTI, Kanglaitei injections. HCSI, Huachansu injections. XAPI, Xiaoaiping injections. SOX, SOX chemotherapy regimens, HQI, Huangqi injections.

The network structure diagram of all outcomes is shown in **Fig. 3.** We tested the degree of model convergence for all outcomes. As can be seen from the trajectory plots and density plots in **Supplement S7**, all chains overlap with each other, and the iterative process of each chain cannot be visually identified. All curves are close to a normal distribution, and the bandwidth values are stable. As can be seen from the Brooks-Gelman-Rubin diagnostic plot in** Supplement S8**, the median and 97.5% values of the reduction factor all tend to be 1. The PSRF values are all 1. Thus, all outcome models have good convergence. The relative effect analysis of outcomes is displayed in** Fig. 4.** We calculated SUCRA values for each outcome for different interventions and used an annular heat map to reflect the ranking of different treatments, as presented in **Fig. 5**.

#### 3.5.1. Primary Outcomes

##### 3.5.1.1. Clinical effectiveness

There were 41 studies with clinical effectiveness as the primary outcome, including 10 with ADI, 1 with SFI, 10 with SQFZI, 4 with FFKSI, 5 with KAI, 4 with KLTI, 2 with HCSI, and 5 with XAPI. Compared with SOX only, ADI+SOX had a RR = 1.46 (95% CI 1.28 to 1.69), SQFZI+SOX a RR = 1.29 (95% CI 1.1 to 1.5), FFKSI+SOX a RR = 1.46 (95% CI 1.15 to 1.89), KAI+SOX a RR = 1.54 (95% CI 1.22 to 2.02), and HCSI+SOX a RR = 1.64 (95% CI 1.17 to 2.37). These CHIs in combination with the SOX chemotherapy regimens significantly improved clinical effectiveness in AGC patients, with statistically significant differences. From the numerical results of SUCRA, it appeared that the intervention order from highest to lowest clinical effectiveness improvement was: HCSI+SOX (SUCRA: 78.17%) > SFI+SOX (SUCRA: 74.76%) > KAI+SOX (SUCRA: 64.16%) > FFKSI+SOX (SUCRA: 61.95%) > SQFZI+SOX (SUCRA: 38.02%) > KLTI+SOX (SUCRA: 34.18%) > XAPI+SOX (SUCRA: 24.32%) > SOX (SUCRA: 2.16%).

##### 3.5.1.2. KPS score

A total of 18 studies reported KPS scores, including 2 with ADI, 1 with SFI, 4 with SQFZI, 1 with KAI, 4 with KLTI, 1 with HCSI, 4 with XAPI, and 1 with HQI. Compared to SOX exclusively, SFI+SOX had a RR = 1.96 (95%CI 1.06 to 3.97), SQFZI+SOX a RR = 1.32 (95%CI 1.03 to 1.78), KLTI+SOX a RR = 1.8 (95% CI 1.28 to 2.57), XAPI+SOX a RR = 1.57 (95% CI 1.19 to 2.14) and HQI+SOX a [RR = 0.58 (95% CI 0.31 to 0.97). Other CHIs did not show statistical significance. The use of SFI+SOX may have the best effect on the KPS score improvement rate (SUCRA: 75.59%) while the use of SOX exclusively may have the least effect (SUCRA: 3.56%).

#### 3.5.2. Secondary outcomes

##### 3.5.2.1. Incidence of leukopenia

A total of 26 studies reported the incidence of leukopenia, including 8 for ADI, 1 for SFI, 6 for SQFZI, 1 for FFKSI, 2 for KAI, 3 for KLTI, 1 for HCSI, 3 for XAPI, and 1 for HQI. The relative effects on the incidence of leukopenia were: Compared with SOX exclusively, ADI+SOX had a RR = 0.64 (95% CI 0.5 to 0.81), SFI+SOX a RR = 0.56 (95% CI 0.33 to 0.91), SQFZI+SOX a RR = 0.49 (95% CI 0.36 to 0.64), SQFZI+KLTI a RR = 0.61 (95% CI 0.4 to 0.9), FFKSI+HCSI a RR = 2.85 (95% CI 1.14 to 8.13), KAI+SOX a RR = 0.53 (95% CI 0.31 to 0.86), HCSI+SOX a RR = 0.29 (95% CI 0.11 to 0.64), HCSI+XAPI a RR = 0.39 (95% CI 0.15 to 0.93) and XAPI+SOX a RR = 0.74 (95% CI 0.53 to 0.99). The AGC patients who used HCSI+SOX had the highest probability of a reduced incidence of leukopenia (SUCRA: 93.35%) while the patients who used SOX exclusively had the lowest (SUCRA: 6.30%).

##### 3.5.2.2. Incidence of thrombocytopenia

A total of 22 studies reported the incidence of thrombocytopenia, including 6 with ADI, 1 with SFI, 4 with SQFZI, 3 with KAI, 2 with KLTI, 1 with HCSI, 4 with XAPI, and 1 with HQI. Compared with SOX exclusively, ADI+SOX had a RR = 0.6 (95% CI 0.38 to 0.95), SQFZI+SOX a RR = 0.47 (95% CI 0.27 to 0.73), SQFZI+KLTI a RR = 0.49 (95% CI 0.22 to 0.98), KAI+SOX a RR = 0.52 (95% CI 0.26 to 0.97), and HCSI+SOX a RR = 0.35 (95% CI 0.11 to 0.96). The SUCRA of AGC patients using HCSI+SOX indicated the greatest probability of reducing the incidence of thrombocytopenia (SUCRA: 80.19%), and the lowest probability was associated with SOX exclusively (SUCRA: 12.76%).

##### 3.5.2.3. Incidence of nausea and vomiting

Overall, 23 studies reported the incidence of nausea and vomiting, including 7 with ADI, 1 with SFI, 4 with SQFZI, 2 with FFKSI, 3 with KAI, 1 with KLTI, 1 with HCSI, 3 with XAPI, and 1 with HQI. The relative effect analysis results for the incidence of nausea and vomiting suggested that compared with SOX chemotherapy alone, ADI+SOX had a RR = 0.7 (95% CI 0.49 to 0.93), SFI+SOX a RR = 0.48 (95% CI 0.22 to 0.97], SQFZI+SOX a RR = 0.42 (95% CI 0.26,0.64), KAI+SOX a RR = 0.61 (95% CI 0.35 to 0.99), and HCSI+SOX a RR = 0.22 (95% CI 0.06 to 0.64). Compared with FFKSI+SOX, the use of SQFZI+SOX had a RR = 0.45 (95% CI 0.21 to 0.99). Compared with KLTI+SOX, the combination SQFZI+SOX had a RR = 0.40 (95% CI 0.18 to 0.86). Compared with HCSI+SOX, ADI+SOX had a RR = 3.16 (95%CI 1.01 to 12.38), FFKSI+SOX a RR = 4.26 (95% CI 1.18 to 18.16), and KLTI+SOX a RR = 4.81 (95% CI 1.35 to 21.05). Compared with XAPI+SOX, the combination HCSI+SOX had a RR = 0.31 (95% CI 0.08 to 0.99). Based on the rank probabilities, the best-performing treatment for reducing the incidence of nausea and vomiting was HCSI+SOX (SUCRA: 95.15%), followed by SQFZI+SOX (SUCRA: 82.10%). KLTI+SOX had minimal effectiveness (SUCRA: 14.68%) but was still better than SOX exclusively (SUCRA: 13.32%).

##### 3.5.2.4. Incidence of liver function damage

A total of 19 studies presented the incidence of liver function damage, including 5 with ADI, 2 with SQFZI, 3 with FFKSI, 3 with KAI, 2 with KLTI, 1 with HCSI, 2 with XAPI, and 1 with HQI. Compared with SOX alone, ADI+SOX had a RR = 0.42 (95% CI 0.21 to 0.8). As the results of SUCRA show, ADI+SOX might be the best choice for reducing the incidence of liver function damage (SUCRA: 75.16%), while SOX alone performed worst (SUCRA: 13.54%).

##### 3.5.2.5. Incidence of peripheral neurotoxicity

31 studies reported the incidence of peripheral neurotoxicity, including 6 with ADI, 1 with SFI, 7 with SQFZI, 3 with FFKSI, 3 with KAI, 4 with KLTI, 2 with HCSI, 4 with XAPI, and 1 with HQI. Compared to SOX alone, ADI+SOX had a RR = 0.40 (95% CI 0.26 to 0.62), SFI+SOX a RR = 0.30 (95% CI 0.11 to 0.64), SQFZI+SOX a RR = 0.60 (95% CI 0.42 to 0.81), KAI+SOX a RR = 0.44 (95% CI 0.26 to 0.67), KLTI+SOX a RR = 0.74 (95% CI 0.57 to 0.96), HCSI+SOX RR = 0.36 (95% CI 0.15 to 0.75), and HQI+SOX a RR = 0.56 (95% CI 0.29 to 1). Compared with FFKSI+SOX, the combination ADI+SOX had a RR = 0.46 (95% CI 0.26 to 0.81), and SFI+SOX a RR = 0.34 (95% CI 0.12 to 0.79). Compared with KAI+SOX, the alternative FFKSI+SOX had a RR = 2.02 (95% CI 1.15 to 3.7). Compared with KLTI+SOX, the combination ADI+SOX had a RR = 0.55 (95% CI 0.32 to 0.90), SFI+SOX a RR = 0.40 (95% CI 0.14 to 0.91), and KAI+SOX a RR = 0.59 (95% CI 0.33 to 0.98). Compared with HCSI+SOX, the combination FFKSI+SOX had a RR = 2.46 (95% CI 1.11 to 6.22). Compared to XAPI+SOX, the alternative ADI+SOX had a RR = 0.48 (95% CI 0.27 to 0.86), SFI+SOX a RR = 0.35 (95% CI 0.12 to 0.84), KAI+SOX a RR = 0.52 (95% CI 0.28 to 0.95), and HCSI+SOX a RR = 0.42 (95% CI 0.17 to 0.97). From the numerical results of SUCRA, SFI+SOX achieved the best reduction in the incidence of peripheral neurotoxicity (SUCRA: 88.26%), and SOX exclusively performed worst (SUCRA: 4.95%).

##### 3.5.2.6. Survival data

We identified survival data in a total of 15 studies. However, due to the lack of standard deviation reporting in some of these studies, we were unable to conduct NMA. Instead, we present the average values of median survival time (MST), median progression-free survival (mPFS), and time to progression (TTP) in **Supplement S9**. Our results demonstrate that the addition of CHIs to the SOX chemotherapy regimen significantly improved MST, mPFS, and TTP compared to the SOX chemotherapy regimen alone. These findings suggest that combining CHIs and chemotherapy may have a positive impact on improving patient survival outcomes.

### 3.6. Publication bias

**Figure 6:**
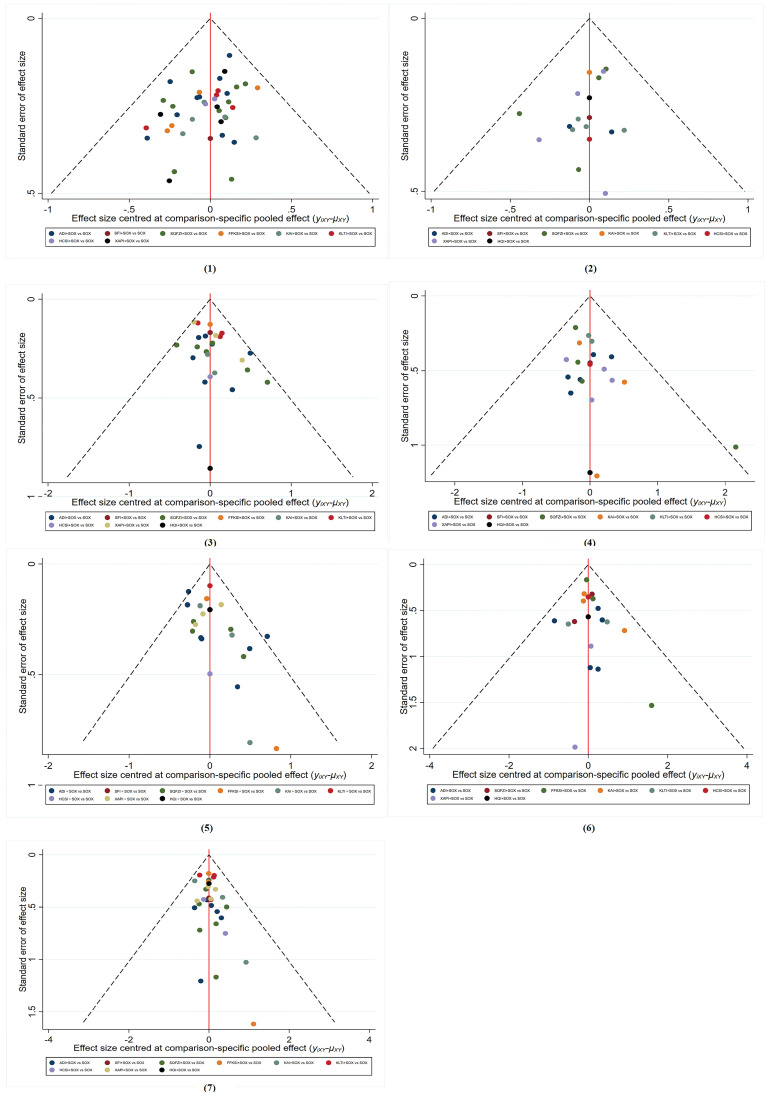
The funnel plot for all outcomes. (1) Clinical effectiveness; (2) Improvement rate of KPS score; (3) Incidence of leukopenia; (4) Incidence of thrombocytopenia; (5) Incidence of nausea and vomiting; (6) Incidence of liver function damage; (7) Incidence of peripheral neurotoxicity. ADI, Aidi injections. SFI, Shenfu injections. SQFZI, Shenqifuzheng injections. FFKSI, Fufangkushen injections. KAI, Kangai injections. KLTI, Kanglaitei injections. HCSI, Huachansu injections. XAPI, Xiaoaiping injections. SOX, SOX chemotherapy regimens. HQI, Huangqi injections.

The funnel plot for all outcomes is presented in **Fig. 6**. The dispersion points in all funnel diagrams were distributed symmetrically. The clinical effectiveness reached P = 0.7286 in the Peters test, the improvement rate of KPS score P = 0.0909, incidence of leukopenia P = 0.5246, incidence of thrombocytopenia P = 0.5574, incidence of nausea and vomiting P = 0.0508, incidence of liver function damage P = 0.1304, and incidence of peripheral neurotoxicity P = 0.3807. The results of the Peters test indicated no publication bias for all outcomes (P > 0.05).

### 3.7. Confidence in evidence

According to CINEMA, most confidence ratings results were “low”, and a few confidence ratings results were “moderate”. Since the network has no closed-loop evidence, the inconsistency cannot be evaluated. Therefore, levels of "incoherence" were all evaluated as "Some concerns". The specific evaluation results are included in the **Supplement CINeMA results.xls**.

## 4. Discussion

The SOX regimen is frequently utilized as the primary chemotherapy regimen for AGC in clinical practice. Yamada et al. have demonstrated that the SOX regimen displays significantly higher rates of progression-free survival and overall survival compared to S-1 plus cisplatin 22. A multicenter, randomized clinical trial conducted in 12 Chinese centers revealed that the SOX regimen is non-inferior to FOLFOX (Fluorouracil plus Oxaliplatin plus Leucovorin) as perioperative chemotherapy for patients with locally AGC. Therefore, SOX should be considered as a viable alternative treatment option for these patients in Asia. Additionally, the study identified a lower incidence of gastrointestinal toxicities (e.g., anorexia or nausea) in the SOX group compared to the FOLFOX group 23. Bando et al. reported that SOX is an effective and feasible treatment for both nonelderly and elderly patients with AGC. In elderly patients, SOX exhibited favorable efficacy and safety compared to S-1 plus cisplatin 24.

Nonetheless, the long-term use of chemotherapy drugs can induce side effects such as bone marrow suppression, gastrointestinal reactions, and peripheral neurotoxicity, thereby reducing patients' tolerance and potentially resulting in treatment discontinuation 25, 26. Some studies have shown that TCM combined with chemotherapy regimens can significantly improve clinical effectiveness and quality of life while reducing adverse reactions 27, 28. CHIs, as a modality of TCM, offer the advantages of convenience and rapid absorption through intravenous administration 29. Consequently, the combined utilization of CHIs and the SOX regimen is becoming increasingly popular in the treatment of AGC. To comprehensively evaluate the clinical effectiveness and safety of various combinations of CHIs with the SOX regimen, this study conducted NMA.

We included 51 studies with 3,703 AGC patients involving 9 types of CHIs in NMA. A random effects model was used for data analysis and there was low heterogeneity among the 51 included studies. According to the NMA results, compared to other CHIs combined with SOX chemotherapy, HCSI plus SOX had the highest rank in improving clinical effectiveness (SUCRA: 78.17%), reducing the incidence of leukopenia (SUCRA: 93.35%), thrombocytopenia (SUCRA: 80.19%), and nausea and vomiting (SUCRA: 95.15%). HCSI is a water-soluble preparation primarily extracted and refined from *Bufo gargarizans* [Bufonidae], containing various compounds such as bufadienolides, indole alkaloids, steroids, and bufotenine amides 30. Among them, bufadienolides are the main active anti-tumor ingredient 31. Bufadienolides can regulate the protein levels of cell cycle proteins and cyclin-dependent kinases (CDKs), leading to tumor cell arrest at the G2/ M phase. Additionally, bufadienolides can increase the expression of the pro-apoptotic gene *Bax* and decrease the expression of the anti-apoptotic gene *Bcl-2*, leading to up-regulation of the apoptotic-related protein *caspase* and promotion of tumor cell apoptosis 32-34. In vitro experiments by Wang and colleagues 35 on AGC cells confirmed that bufadienolides can effectively inhibit the proliferation, invasion, and metastasis of AGC cells. The use of HCSI controlled the progression of the disease, leading to the remission of symptoms and signs in advanced patients, thereby improving clinical effectiveness. Furthermore, HCSI exerts a positive impact on the cellular and humoral immune functions in patients with advanced tumors. It enhances the phagocytic activity of macrophages, improves the activity of natural killer cells and T cell subsets, and strengthens overall immune function. As a result, it reduces the incidence of leukopenia 36-39. Our NMA analysis demonstrated that HCSI significantly reduced the incidence of thrombocytopenia (SUCRA: 80.19%). This could be attributed to HCSI's ability to improve coagulation function. Specifically, an RCT validated our findings by demonstrating that HCSI treatment significantly enhances coagulation function and improves survival quality in patients with AGC 40. Furthermore, our research found the remarkable effectiveness of HCSI in reducing nausea and vomiting (SUCRA: 95.15%). However, the limited number of published studies on this topic indicates a potential area for future research.

According to our data analysis results, compared to other CHIs, SFI plus SOX was the best option for improving the KPS score (SUCRA: 75.59%) and reducing the incidence of peripheral neurotoxicity (SUCRA: 88.26%). SFI is a water-soluble preparation mainly composed of *Panax ginseng C.A. Mey.* [Araliaceae] and *Aconitum carmichaeli Debeaux* [Ranunculaceae] 41. Its chemical components mainly include ginsenosides, and aconite alkaloids 42. Ginsenosides have effects including enhancing T cell proliferation, inhibiting apoptosis, and indirectly inhibiting the growth of tumor cells 43. Ginsenosides can improve the immunity of mice, reduce the expression of PD-L1 induced by chemoresistance, and restore the cytotoxicity of T cells toward cancer cells 44. Aconite alkaloids can achieve anti-tumor effects by reducing the spreading ability of cancer cells. Its mechanism may be related to the inhibition of the activation of the P38MAPK signaling pathway 45. Animal experiments conducted by Liu et al. 46 have shown that aconite alkaloids can significantly inhibit the transformation function of mouse T lymphocytes, while significantly inhibiting the secretion of IL-l and TNF-α from peritoneal macrophages, and also have a significant inhibitory effect on the expression of CD91 and CD13 on macrophages, thereby regulating the immune function of the body. Clinical studies have found that Shenfu injections can improve the immunity of patients and significantly improve their quality of life 47. In addition, Wei et al. 48 have confirmed that SFI can reduce the peripheral neurotoxicity of oxaliplatin. Therefore, SFI has the potential to improve the quality of life of patients and peripheral neurotoxicity. ADI is composed of *Panax ginseng C.A. Mey.* [Araliaceae], *Eleutherococcus senticosus (Rupr. and Maxim.) Maxim* [Araliaceae], *Astragalus membranaceus (Fisch.) Bunge* [Fabaceae] and *Harmonia axyridis (Pallas)* [Coccinellidae] 49. ADI chemical components include ginsenosides, *Eleutherococcus senticosus* glycosides, Astragalus saponins, and *Buthus martensii* toxin. Ginsenosides and *Eleutherococcus senticosus* glycosides have good antioxidant effects 50. *Buthus martensii* extract has a dual effect of anti-tumor and immune regulation and can protect liver cells while having anti-tumor properties 51. Therefore, ADI plus SOX was the best-performing choice for reducing the incidence of liver function damage (SUCRA: 75.16%).

In addition to RCTs demonstrating the clinical effectiveness and reduced adverse events of CHIs in combination with the SOX for AGC, real-world studies have also been conducted. The analysis and processing of real-world clinical data are pivotal for transforming the individualized empirical laws of TCM into sophisticated medical evidence 52. For instance, an observational study by Ai et al., which involved 71 patients of AGC revealed that the combination of SOX chemotherapy regimen and ADI reduced patients' vascular endothelial growth factor levels, ameliorated cancer-related fatigue, and boosted immune function 53. Similarly, Gao Y administered SQFZI in combination with SOX to 40 patients with AGC. The study indicated that this treatment significantly improved the disease control rate, was safe, and considerably enhanced the quality of survival 54.

Nevertheless, it is important to recognize that extant real-world studies on the SOX combined with CHIs primarily rely on small samples. This approach may engender certain issues. Firstly, the results derived from small-sample studies may lack stability and be subject to randomness. Secondly, such studies may not adequately represent the treatment response and disease progression in large patient groups, thus limiting the generalizability and representativeness of their findings. Lastly, due to the restricted sample size, certain potential therapeutic impacts or adverse reactions may remain undetected, possibly influencing the refinement and enhancement of treatment strategies 55, 56. We anticipate more large-sample real-world studies in the future to augment the reliability and representativeness of the findings. We also aspire for the ongoing improvement and innovation of research methodologies, such as the incorporation of more advanced data analysis and processing tools, to better explore and utilize real-world clinical data. This would further bolster the capability of TCM in treating AGC and pave the way for personalized and precise medical services.

Our NMA has particular strengths. We employed the Bayesian model, which is the most applicable approach for conducting multiple-intervention NMA, to evaluate the clinical effectiveness of CHIs in combination with SOX for the treatment of AGC. This application of the Bayesian model addressed the lack of direct comparisons between CHIs and revealed a favorable intervention through ranking analysis of various outcomes. Moreover, a thorough search and a pre-defined inclusion criterion were implemented to minimize clinical heterogeneity to the greatest extent possible 57. In terms of clinical research, we closely followed current clinical treatment trends. Based on published RCTs, our NMA included 9 commonly used CHIs for the treatment of AGC. We conducted subgroup analysis, meta-regression analysis, and sensitivity analysis to discuss the sources of heterogeneity. We used funnel plots to detect publication bias and further validated results with the Peters test. We conducted SUCRA rankings for each treatment measure and performed statistical analyses to determine the statistical significance of the comparisons between different treatment measures. The implementation of NMA followed the PRISMA-NMA guidelines. Furthermore, we conducted a comprehensive evaluation of evidence for the comparisons between multiple treatment measures for each outcome.

However, our study has certain limitations. In terms of the methodological evaluation of the included 51 studies, we found that none of the included studies mentioned whether participants and outcome assessors were blinded, which may result in bias and lack of objectivity. In addition, 10 studies did not report clinical effectiveness and selective reporting cannot be ruled out. The small sample size of the different CHIs reduces the stability and accuracy of the results. Furthermore, none of the 51 studies included in our analysis had registered clinical trial protocols. However, registering clinical trial protocols is crucial not only for fulfilling ethical obligations towards participating subjects and researchers but also for providing reference information to patients and physicians. Additionally, it plays a pivotal role in mitigating publication bias in medical literature research. Moreover, registering clinical trial protocols aids medical editors in understanding trial results, promotes effective investment and allocation of research funds, and assists ethics practitioners in assessing the appropriateness of study 58. Moving forward, we anticipate that clinical trials investigating the combination of chinese herbal injections with the SOX chemotherapy regimen for the treatment of AGC will prioritize the registration of trial protocols before implementation. This step will ensure transparency throughout the design, execution, and completion of the trials, thereby guaranteeing the traceability of the studies. Since the network has no closed-loop evidence, inconsistency could not be assessed. The CINeMA shows that most confidence rating results were “low”. At the same time, the results cannot be extrapolated as the included studies are all from Chinese studies and the participants are all of Chinese heritage. Therefore, we propose in the future to generate larger and more methodologically rigorous RCTs for CHIs in combination with the SOX chemotherapy regimen, including in different countries. Furthermore, it is crucial to conduct more pharmacological studies to further verify the safety of CHIs in the treatment of patients with AGC. While none of the included studies discussed health economic aspects, we also encourage such studies to understand the price and treatment effects of CHIs, and tailor appropriate treatment plans for patients according to their conditions and taking into account economic considerations 59, thereby supporting the selection of the optimal solution for AGC patients according to the clinical effectiveness, safety and economy of CHIs.

## Conclusion

In conclusion, CHIs in combination with SOX have demonstrated a positive effect on the treatment of AGC patients compared to the use of SOX alone. HCSI and SFI injections potentially have the most pronounced integrated advantage of all CHIs. ADI can be considered the optimal choice for reducing the incidence of liver function damage. More methodologically rigorous RCTs with larger sample sizes and additional pharmacological studies are needed to support this evidence. Health economic studies of CHIs should also be conducted to select the optimal solution for AGC patients based on clinical effectiveness, safety, and economy.

## Supplementary Material

Supplementary tables.

Supplementary CINeMA results.

## Figures and Tables

**Table 1:** Characteristics of the included studies.

Study ID	Gender (male/female)	Sample size	Mean/ median Age(I/C)	Treatment (intervention vs control)	Intervention details	Control details	Outcome details
Xue TL 2023^60^	62/36	I, 49 C, 49	I, 54.81±8.93 C, 53.47±8.62	D versus.I	FFKSI, 5 ml, qd, course=21d*4, +Controls.	Oxaliplatin, 130 mg/m^2^, qd, d1, course=21d. Tegafur, 40 mg (BSA<1.25m^2^), 50 mg (1.25m^2^<BSA<1.5m^2^), 60 mg (BSA>1.5m^2^), bid, d1~d14, course=21d*4.	①③⑤ ⑥⑦
Han B 2022^61^	52/23	I, 40 C, 35	I, 62.13±6.85 C, 62.05±6.74	D versus.I	FFKSI, 5 ml, qd, d1~d21, course=21d*2, +Controls.	Oxaliplatin, 130 mg/m^2^, qd, d1, course=21d*2. Tegafur, 40 mg (BSA<1.25 m^2^), 50 mg (1.25m^2^<BSA<1.5m^2^), 60 mg (BSA>1.5 m^2^), bid, d1~d14, course=21d*2.	①⑤⑥ ⑦
Yang QW 2021^62^	33/38	I, 37 C, 34	I, 64.73±6.52 C, 62.60±5.91	F versus.I	KLTI, 200 ml, qd, d1~d7, course=21d*3, +Controls.	Oxaliplatin, 130 mg/m^2^, qd, d1, course=21d*3. Tegafur, 40 mg (BSA<1.25m^2^), 50 mg (1.25m^2^<BSA<1.5m^2^), 60 mg (BSA>1.5m^2^), bid, d1~d14, course=21*3.	①⑤⑥
Yang Q 2021^63^	35/25	I, 30 C, 30	I, 60±3 C, 60±3	A versus.I	ADI, 10 ml, qd, d1~d21, course=21d*3, +Controls.	Oxaliplatin, 130 mg/m^2^, qd, d1, course=21d*3. Tegafur, 40 mg (BSA<1.25m^2^), 100 mg (1.25m^2^<BSA<1.5m^2^), 120 mg (BSA>1.5m^2^), d1~d14, course=21d*3.	①③④
Si LL 2021^64^	47/31	I, 39 C, 39	I, 60.28±9.54 C, 56.85±9.15	E versus.I	KAI, 20 ml, qd, d1~d14, course=21d, +Controls.	Oxaliplatin, 130 mg/m^2^, qd, d1, course=21d*6. Tegafur, 40 mg/m^2^, bid, d1~d14, course=21d*6.	①③④ ⑤⑥⑦
Ruan XJ 2021^65^	46/38	I, 42 C, 42	NM	H versus.I	XAPI, 20 ml, d1~d14, course =14d, + Controls.	Oxaliplatin, 130 mg/m^2^, qd, d1. Tegafur, 40 mg (BSA<1.25m^2^), 50 mg (1.25m^2^<BSA<1.5m^2^), 60 mg (BSA>1.5m^2^), d1~d14, course=21d.	①②④ ⑤⑥⑦
Dou SS 2021^66^	40/32	I, 36 C, 36	I, 57.0±3.2 C, 56.3±2.4	G versus.I	HCSI, 5 ml, qd, d1~d14, course=14d, +Controls.	Oxaliplatin, 85 mg/m^2^, qd, d1. Tegafur, 40 mg (BSA<1.25m^2^), 50 mg (1.25m^2^<BSA<1.5m^2^), 60 mg (BSA>1.5m^2^), bid, d1~d14, course=21d*3.	①②⑤ ⑥⑦⑧
Chang ZG 2021^67^	62/44	I, 53 C, 53	I, 63.74±7.85 C, 64.63±8.25	D versus.I	FFKSI, 20 ml, qd, d1~d21, course=21d*2, +Controls.	Oxaliplatin, 130 mg/m^2^, qd, d1, course=21d*2. Tegafur, 80 mg/m^2^, bid, d1~d14, course=21d*2.	①⑤⑥ ⑦⑧
Zhao HB 2020 68	78/72	I, 75 C, 75	I, 43.14±6.79 C, 42.52±6.83	A versus.I	ADI,10 ml, course =21d*6. +Controls.	Oxaliplatin, 130 mg/m^2^, qd, d15~d21, course=21d*6. Tegafur, bid, d1~d14, course=21d*6.	①③④ ⑤⑥⑧
Zhang MM 2020^69^	49/41	I, 45 C, 45	I, 52.6±9.15 C, 52.8±8.43	H versus.I	HQI, 250 ml, +Controls.	NM	⑥⑦
Shi ZW 2020^70^	44/38	I, 41 C, 41	I, 50.01±3.22 C, 49.34±3.17	C versus.I	SQFZI,250 ml, qd, d1~d10, course=14d*2, +Controls.	Oxaliplatin,130 mg/m^2^, d1, course=21d. Tegafur, 40 mg/m^2^, bid, d1~4, course=18d*2.	①
Qian YM 2020^71^	58/46	I, 52 C, 52	I, 54.85±4.01 C, 53.44±3.99	A versus.I	ADI, 50 ml, qd, d1~d21, course=84d, +Controls.	Oxaliplatin, 130 mg/m^2^, bid. Tegafur, 40 mg (BSA<1.25m^2^), 50 mg (1.25m^2^<BSA<1.5m^2^), 60 mg (BSA>1.5m^2^), bid, d1~d14, course=21d*3.	
Li RQ 2020^72^	41/21	I, 32 C, 30	I, 67.20±6.92 C, 66.74±7.35	A versus.I	ADI, 40 ml, qd, d1~21, course=21d*4, +Controls.	Oxaliplatin, 130 mg/m^2^, d1. Tegafur, 40 mg (BSA<1.25m^2^), 60 mg (BSA>1.25m^2^), bid, d1~d14, course=21d*4.	①⑧
Chen YX 2020^73^	46/44	I, 45 C, 45	I, 46.93±6.9 C, 46.77±6.83	A versus.I	ADI, 50 ml, qd, d1~d15, course=18*10d, +Controls.	Oxaliplatin, 130 mg/m^2^, d1, course=18d*3. Tegafur, 40 mg (BSA<1.25m^2^), 50 mg (1.25m^2^<BSA<1.5m^2^), 60 mg (BSA>1.5m^2^), bid, d1~d28, course=42*4d.	①⑦
Chen XT 2020^74^	94/26	I, 60 C, 60	I, 58.5±2.3 C, 58.6±2.5	C versus.I	SQFZI, 250 ml, qd, d1~d10, course=14d*3, +Controls.	Oxaliplatin, 130 mg/m^2^, qd, d1~d4. Tegafur, 40 mg/m^2^, bid, d1~d14, course=NM.	①③④⑧
Wang P 2019^75^	82/58	I, 70 C, 70	I, 57.25±2.85 C, 57.7±2.31	C versus.I	SQFZI, 250 ml, qd, d1~d21, course=21d, +Controls.	Oxaliplatin, 130 mg/m^2^, d1. Tegafur,40 mg (BSA<1.25m^2^), 50 mg (1.25m^2^<BSA<1.5m^2^), 60 mg (BSA>1.5m^2^), bid, d1~d14, course=21d.	①⑦
Xu RQ 2019^76^	54/32	I, 43 C, 43	I, 60.7±5.4 C, 63.2±4.9	F versus.I	KLTI, qd, d1~d10, course=10d, +Controls.	Oxaliplatin, 130 mg/m^2^, d1, course=21d*2~8. Tegafur, 40~60 mg, bid, d1~d14, course=21d*2~8.	②③④ ⑦
Song B 2019^77^	28/38	I, 33 C, 33	I, 66.27±7.73 C, 66.51±7.62	C versus.I	SQFZI, 250 ml, qd, d1~d2, course=21d*4, +Controls.	Oxaliplatin, 130 mg/(m^2^·d), d1, course=21d*4. Tegafur, 80 mg/(m^2^·d), bid, d1~d14, course=21d*4.	①
Wu Y 2019^78^	37/23	I, 32 C, 28	NA	E versus.I	KAI, 60 ml, d1~d14, course=21d*2, +Controls.	Oxaliplatin, NA. Tegafur,40 mg/m^2^, bid, d1~d14, course=21d*2.	①②⑤ ⑥⑦
Gao CL 2019^79^	49/11	I, 30 C, 30	I, 69.53±6.04 C, 67.77±6.10	C versus. I	SQFZI, 250 ml, qd, d1~d14, course=14d*2, +Controls.	Oxaliplatin, 130 mg/m^2^, qd, d1, course=21d*2. Tegafur, 40 mg (BSA<1.25m^2^), 50 mg (1.25m^2^<BSA<1.5m^2^), 60 mg (BSA>1.5m^2^), bid, d1~d14, course=21d*2.	②③④ ⑤⑥⑦
Dong L 2019^80^	36/28	I, 32 C, 64	I, 60.04±4.35 C, 59.62±3.72	G versus.I	HCSI,20 ml, qd, course=21d, +Controls.	Oxaliplatin,80 mg/m^2^, qd, d1, course=21d*3. Tegafur, 110 mg, bid, d1~d15, course=21d*3.	①③④ ⑦
Wang R 2018^81^	43/35	I, 39 C, 39	I, 52.51±5.83 C, 51.46±7.18	A versus.I	ADI, 50~60 ml, qd, d10~d14, course=14d, +Controls.	Oxaliplatin,100~130 mg/m^2^, bid, d1~d14, course=21*7d. Tegafur, 40 mg (BSA<1.25m^2^), 50 mg (1.25m^2^<BSA<1.5m^2^), 60 mg (BSA>1.5m^2^), bid, d1~d14, course=21*7d.	①③⑦
Rao JJ 2018^82^	49/41	I, 45 C, 45	I, 58.12±3.25 C, 58.27±3.11	A versus.I	ADI, 50~60 ml, qd, d10~d14, course=14d, +Controls.	Oxaliplatin,100~130 mg/m^2^, bid, d1~d14, course=21*7d. Tegafur, 40 mg (BSA<1.25m^2^), 50 mg (1.25m^2^<BSA<1.5m^2^), 60 mg (BSA>1.5m^2^), bid, d1~d14, course=21*7d.	①
Liu HT 2018^83^	33/27	I, 30 C, 30	I, 62.0±4.3 C, 64.1±3.2	C versus.I	SQFZI, qd, +Controls.	Oxaliplatin, 130 mg/m^2^, d1, course=21d. Tegafur, 40~60 g, bid, d1~d14, course=21d.	⑦
Gao NN 2018^84^	31/17	I, 24 C, 24	I, 57.47±7.95 C, 57.73±8.52	F versus.I	KLTI, 200 ml, qd, d1~d14, course=21d*2, +Controls.	Oxaliplatin, 130 mg/m^2^, d1, course=21d. Tegafur, 80 mg (BSA<1.25 m^2^), 100 mg (1.25m^2^<BSA<1.5m^2^), 120 mg (BSA>1.5m^2^), bid, d1~d14, course=21d*2.	①②③ ⑦
Xu JL 2017^85^	55/39	I, 47 C, 47	I, 53.42±3.96 C, 54.29± 4.11	A versus.I	ADI, 50 ml, qd, d1~d21, course=21d*4, +Controls.	Oxaliplatin, 130 mg/m^2^, qd, d1. Tegafur, 40 mg (BSA<1.25m^2^), 50 mg (1.25m^2^<BSA<1.5m^2^), 60 mg (BSA>1.5m^2^), bid, d1~d14, course=21d.	①③④ ⑤⑥⑦ ⑧
Tang XF 2017^86^	34/27	I, 31 C, 30	I, 60.69±3.13 C, 59.16±3.15	C versus.I	SQFZI, 250 ml, qd, course=21d*2, +Controls.	Oxaliplatin, 130 mg/m^2^. Tegafur, 40 mg/m^2^, bid, d1~d14, course=21d*2.	①④⑤ ⑥⑦⑧
Shen G 2017^87^	69/35	I, 54 C, 50	NR	F versus.I	KLTI, qd, d1~d10, course=10d, +Controls.	Oxaliplatin, 130 mg/m^2^, d1. Tegafur, 40~60 mg/m^2^, bid, d1~d14, course=21d.	①②③ ④⑦
Pang YP 2017^88^	43/41	I, 42 C, 42	I, 70.08±5.49 C, 70.01±5.14	E versus.I	KAI,60 ml, course=20d*2, +Controls.	Oxaliplatin,100 mg/m^2^, d1, course=20d*2. Tegafur, NM.	①④
Liu W 2017^89^	63/33	I, 48 C, 48	I, 54.25±8.56 C, 55.33±9.98	Hversus.I	XAPI, 20 ml, d1~d14, course=14d, +Controls.	Oxaliplatin, 130 mg/m^2^, d1, course=21d. Tegafur, 80 mg/m^2^, bid, d1~d14, course=21d.	①②③ ④⑦
Li CH 2017^90^	24/11	I, 18 C, 17	42~72	C versus.I	SQFZI, 250 ml, qd, d1~d14, course=21d*4, +Controls.	Oxaliplatin, 130 mg/m^2^, qd, d1. Tegafur, 40 mg (BSA<1.25m^2^), 50 mg (1.25m^2^<BSA<1.5m^2^), 60 mg (BSA>1.5m^2^), bid, d1~d14, course=21d*4.	③
Hu Q 2017^91^	22/14	I, 18 C, 18	I, 69.33±9.61 C, 71.60±6.72	E versus.I	KAI, 50 ml, qd, d1~d14, course=21d*6, +Controls.	Oxaliplatin, 130 mg/m^2^, qd, d1. Tegafur, 60 mg, bid, d1~d14, course=21d*6.	①③④ ⑤⑥⑦ ⑧
Yan LF 2016^92^	22/22	I, 22 C, 22	I, 55.6±6.8 C, 56.8±6.7	H versus.I	HQI, 3 ml, qd, d1~d10, course=10d*2, +Controls.	Oxaliplatin, 130 mg/m^2^, qd, d1. Tegafur, 40 mg/m^2^, bid, d1~d14, course=21d.	②
Xu SG 2016^93^	25/15	I, 20 C, 20	I, 65.11±6.56 C, 65.12±6.21	H versus.I	HQI, 2 ml, qd, d1~d8, course=10d, +Controls.	Oxaliplatin, 85 mg/m^2^, qd, d1. Tegafur, 40 mg (BSA<1.25m^2^), 50 mg (1.25m^2^<BSA<1.5m^2^), 60 mg (BSA>1.5m^2^), bid, course=21d.	③④
Xie JF 2016^94^	46/35	I, 41 C, 40	I, 63.86±8.57 C, 62.74±8.62	E versus.I	KAI, 30 ml, qd, d1~d30, course=30d, +Controls.	Oxaliplatin, 130 mg/m^2^, qd, d1. Tegafur, 80 mg/m^2^, d1~d14, course=28d*2.	①
Ma YK 2017^95^	33/27	I, 30 C, 30	I, 70.14±6.51 C, 70.11±4.51	D versus.I	FFKSI, 20 ml, qd, course=21d*3, +Controls.	Oxaliplatin, 130 mg/m^2^, d1. Tegafu, 40 mg (BSA<1.25m^2^), 50 mg (1.25m^2^<BSA<1.5m^2^), 60 mg (BSA>1.5m^2^), bid, d1~d4, course=21d*3.	①
Gao M 2017^96^	35/31	I, 30 C, 30	NA	H versus.I	XAPI, 80 ml, qd, d1~d14, course=14d*4, +Controls.	Oxaliplatin, 80~100 mg/m^2^, d1, course=21d*4. Tegafur, 40 mg/m^2^, bid, d1~d14, course=21d*4.	①②③ ④⑤⑥ ⑦
Yin Q 2015^97^	25/18	I, 22 C, 21	I, 54.6±13.5 C, 53.8±12.8	C versus.I	SQFZI, 250 ml, qd, d1~d7, course=7d, +Controls.	Oxaliplatin, 130 mg/m^2^, qd, d1. Tegafur, 40 mg (BSA<1.25m^2^), 50 mg (1.25m^2^<BSA<1.5m^2^), 60 mg (BSA>1.5m^2^), bid, d1~d14, course=21d*4.	⑧
Yao XJ 2015^98^	28/21	I, 27 C, 22	I, 71~89 C, 71~87	F versus.I	KLTI, qd, d1~d14, course=21d*2, +Controls.	Oxaliplatin, 130 mg/m^2^, course=21d*2. Tegafur, 80~120 mg, bid, d1~d14, course=21d*2.	①②⑤ ⑥⑦
Xiong L 2015^99^	38/26	I, 32 C, 32	I, 51.32±10.18 C, 51.32±10.18	H versus.I	XAPI, 80 ml, qd, d1~d21, course=21d*4, +Controls.	Oxaliplatin, 130 mg/m^2^, qd, d1. Tegafur, 80 mg/m^2^, d1~d14, course=21d*4.	①⑦⑧
Ma YJ 2015^100^	26/20	I, 23 C, 23	I, 61.5 C, 63.1	H versus.I	XAPI, course=7d*2, +Controls.	Oxaliplatin, 85 mg/m^2^, d1, course=21d*2. Tegafur, 40 mg, bid, d1~d14, course=21d*2.	①②③ ④
Jiang J 2015^101^	19/11	I, 15 C, 15	I, 53.6±2.6 C, 54.3±2.7	C versus.I	SQFZI,qd, course=21d*4, +Controls.	Oxaliplatin, 130 mg/m^2^, d1, course=21d*4. Tegafur, 80 mg/m^2^, bid, d1~d14, course=21d*4.	①⑦⑧
Yang ZY 2014^102^	52/37	I, 46 C, 40	I, 70±5.2 C, 69±4.5	C versus.I	SQFZI, 250 ml, qd, course=21d*2, +Controls.	Oxaliplatin, 130 mg/m^2^, qd, d1. Tegafur, 40~60mg/m^2^, bid, d1~d14, course=21d*2.	①③④ ⑦
Xie YG 2014^103^	46/31	I, 40 C, 37	I, 57.44±10.08 C, 55.79±9.31	B versus.I	SFI, 100 ml, qd, d1~d14, course=14d, +Controls.	Oxaliplatin, 135 mg/m^2^, qd, d1. Tegafur, 40 mg (BSA<1.5m^2^), 60 mg (BSA>1.5m^2^), bid, d1~d14, course=21d.	①②③ ④⑦
Wang J 2013^104^	68/32	I, 50 C, 50	I, 57.4±5.9 C, 59.1±4.6	A versus.I	ADI, qd, d1~d14, course=21d*4, +Controls.	Oxaliplatin, 130 mg/m^2^, qd, d1, course=21d*4. Tegafur, 80 mg/m^2^, qd, d1~d14, course=21d*4.	①③④ ⑤⑥⑦
Sun GZ 2013^105^	22/16	I, 20 C, 18	53.6	C versus.I	SQFZI, qd, d1~d14, course=14d*2, +Controls.	Oxaliplatin, 100 mg/m^2^, d1, course=21d*2. Tegafur, 40 mg/m^2^, bid, d1~d21, course=21d*2.	①②③
Li YQ 2013^106^	28/30	I, 30 C, 28	NR	A versus.I	ADI, 60~100 ml, qd, d1~d4, course=4d*6, +Controls.	Oxaliplatin, 130 mg/m^2^, qd, d1. Tegafur, 80 mg/m^2^, bid, d1~d14, course=21d*2.	①②③ ⑤⑦⑧
Zhang WH 2012^107^	20/10	I, 20 C, 10	NR	C versus.I	SQFZI, 250 ml, qd, d1~d14, course=36d*4, +Controls.	Oxaliplatin, 100 mg/m^2^, qd, d8. Tegafur, 40 mg (BSA<1.25m^2^), 50 mg(1.25m^2^<BSA<1.5m^2^), 60 mg (BSA>1.5m^2^), bid, d1~d21, course=36d*4.	①②③ ⑧
Ruan XJ 2012^108^	28/26	I, 27 C, 27	NR	C versus.I	SQFZI, 250 ml, qd, d1~d14, course=21d*2, +Controls.	Oxaliplatin, 100 mg/m^2^, qd, d1. Tegafur, 40 mg/m^2^, bid, d1~d21, course=21d*2.	①②⑦
Liu HZ 2012^109^	38/18	I, 28 C, 28	NR	A versus.I	ADI, 60 ml, qd, d1~d14, course=21d*4, +Controls.	Oxaliplatin, 130 mg/m^2^, qd, d1. Tegafur, 80 mg/m^2^, bid, d1~d14, course=21d*4.	④③⑤ ⑥⑦⑧
Fan CM 2011^110^	33/18	I, 23 C, 28	NR	A versus.I	ADI, 50 ml, qd, d1~d10, course=10d*4, +Controls.	Oxaliplatin, 85mg/m^2^, qd, d1. Tegafur, 80 mg/m^2^, bid, d1~d14, course=21d.	①②③ ④⑤⑥ ⑧

Abbreviations in Table 1, I, intervention group, C, control group, NM, not mentioned, qd, one time a day, bid, two times a day, tid, three times a day. The specific meaning of treatment column, A, AiDi injections (ADI), B, Shenfu injections (SFI), C, Shenqifuzheng injections (SQFZI), D, Fufangkushen injections (FFKSI), E, Kangai injections (KAI), F, Kanglaitei injections (KLTI), G, Huachansu injections (HCSI), H, Xiaoaiping injections (XAPI), I, SOX chemotherapy regimens, J, Huangqi injections (HQI). BSA, body surface area. The meaning of the number represented in the Outcome details, ①, Clinical effectiveness. ②, Quality of life improvement rate. ③, Incidence of leukopenia. ④, Incidence of thrombocytopenia. ⑤, Incidence of nausea and vomiting. ⑥, Incidence of liver function damage. ⑦, Incidence of peripheral neurotoxicity. ⑧, Survival data
