# Supplementary material for: Effectiveness and Safety of Chinese Herbal Injections Combined with SOX Chemotherapy Regimens for Advanced Gastric Cancer: a Bayesian Network Meta-Analysis
Source: J Cancer. 2024 Jan 1;15(4):889–907. doi: 10.7150/jca.91301 (PMC10788720; doi:10.7150/jca.91301)
Supplement: Supplementary file 1 — Supplementary tables. [file jcav15p0889s1.pdf]

Supplement

Effectiveness and safety of Chinese herbal injections combined with SOX chemotherapy regimens for advanced gastric cancer: A Bayesian network meta-analysis

Zhi-jun Bu, Shu-run Wan, Peter Steinmann, Ze-tao Yin, Jin-ping Tan, Wen-xin Li, Zhen-yan Tang, Shuo Jiang, Meng-meng Ye, Jin-yang Xu, You-you Zheng, Xue-hui Wang, Jian-ping Liu, Zhao-lan Liu

Corresponding author at: Centre for Evidence-Based Chinese Medicine, Beijing University of Chinese Medicine, Sunshine South Street, Fangshan District, Beijing 102400, China.

E-mail address: 20210941076@bucm.edu.cn (Z.-L. Liu).

Supplement S1

Table S1, PRISMA checklist for our NMA.

Section/Topic	Item	Checklist Item	Reported on Page
**TITLE**<br>Title	1	Identify the report as a systematic review incorporating a network meta-analysis (or related form of meta-analysis).	Page 1.
**ABSTRACT**<br>Structured summary	2	Provide a structured summary including, as applicable:<br>**Background:** main objectives<br>**Methods:** data sources; study eligibility criteria, participants, and interventions; study appraisal; and synthesis methods, such as network meta-analysis.<br>**Results:** number of studies and participants identified; summary estimates with corresponding confidence/credible intervals; treatment rankings may also be discussed. Authors may choose to summarize pairwise comparisons against a chosen treatment included in their analyses for brevity.<br>**Discussion/Conclusions:** limitations; conclusions and implications of findings.<br>**Other:** primary source of funding; systematic review registration number with registry name.	Page 1.
**INTRODUCTION**<br>Rationale	3	Describe the rationale for the review in the context of what is already known, including mention of why a network meta- analysis has been conducted.	Page 2.<br>In the **1. Introduction** section
Objectives	4	Provide an explicit statement of questions being addressed, with reference to participants, interventions, comparisons, outcomes, and study design (PICOS).	Page 4.<br>In the **1. Introduction** section
**METHODS**	5	Indicate whether a review protocol exists and if	Page 4.

Protocol and registration		and where it can be accessed (e.g., Web address); and, if available, provide registration information, including registration number.	In the **2. Methods** section
Information sources	6	Describe all information sources (e.g., databases with dates of coverage, contact with study authors to identify additional studies) in the search and date last searched.	Page 4. In the 2.1. **Search strategy** of the **2. Methods** section
Search	7	Present full electronic search strategy for at least one database,including any limits used, such that it could be repeated.	Page 4. In the 2.1. **Search strategy** of the **2. Methods** section
Eligibility criteria	8	Specify study characteristics (e.g., PICOS, length of follow-up) and report characteristics (e.g., years considered, language, publication status) used as criteria for eligibility, giving rationale. Clearly describe eligible treatments included in the treatment network, and note whether any have been clustered or merged into the same node (with justification).	Page 4. In the 2.2. **Eligibility criteria** of the 2. **Methods** section
Study selection	9	State the process for selecting studies (i.e., screening, eligibility, included in systematic review, and, if applicable,included in the meta-analysis).	Page 5. In the 2.3. **Study selection** of the **2.Methods** section
Data collection process	10	Describe method of data extraction from reports (e.g., piloted forms, independently, in duplicate) and any processes for obtaining and confirming data from investigators.	Page 5. In the 2.3. **Study selection** of the **2.Methods** section
Data items	11	List and define all variables for which data were sought (e.g.,PICOS, funding sources) and any assumptions and simplifications made.	Page 5. In the 2.3. **Study selection** of the **2.Methods** section
**Geometry of the network**	**S1**	Describe methods used to explore the geometry of the treatment network under study and potential biases related to it.This should include how the evidence base has been graphically summarized for presentation, and what characteristics were compiled and used to describe the evidence base to readers.	Page 6. In the 2.5. **Statistical analysis** of the **2. Methods** section
Risk of bias within individual studies	12	Describe methods used for assessing risk of bias of individual studies (including specification of whether this was done at the study or outcome level), and how this information is to be used in any data synthesis.	Page 5. In the 2.4. **Risk of bias and evidence quality assessment** of the **2. Methods** section
Summary measures	13	State the principal summary measures (e.g., risk ratio, difference in means). Also describe the use of additional summary measures assessed, such as treatment rankings and surface under the cumulative ranking curve (SUCRA) values,as well as modified approaches used to present summary findings from meta-analyses.	Page 6. In the 2.5. **Statistical analysis** of the **2. Methods** section
Planned methods of analysis	**14**	Describe the methods of handling data and combining results of studies for each network meta-analysis. This should include,but not be limited to:<br>Handling of multi-arm trials;<br>Selection of variance structure;	Page 6. In the 2.5. **Statistical analysis** of the **2. Methods** section

		Selection of prior distributions in Bayesian analyses; And Assessment of model fit.	
**Assessment of Inconsistency**	**S2**	Describe the statistical methods used to evaluate the agreement of direct and indirect evidence in the treatment network(s) studied. Describe efforts taken to address its presence when found.	Page 6. In the 2.5. **Statistical analysis** of the **2. Methods** section
Risk of bias across studies	**15**	Specify any assessment of risk of bias that may affect the cumulative evidence (e.g., publication bias, selective reporting within studies).	Page 5. In the 2.4. **Risk of bias and evidence quality assessment** of the **2. Methods** section
Additional analyses	**16**	Describe methods of additional analyses if done, indicating which were pre-specified. This may include, but not be limited to, the following: Sensitivity or subgroup analyses; Meta-regression analyses; Alternative formulations of the treatment network; and Use of alternative prior distributions for Bayesian analyses (if applicable).	Page 6. In the 2.5. **Statistical analysis** of the **2. Methods** section
**RESULTS†** Study selection	**17**	Give numbers of studies screened, assessed for eligibility, and included in the review, with reasons for exclusions at each stage, ideally with a flow diagram.	Page 6. In the 3.1. **Study selection** of the **3.Results** section
**Presentation of network structure**	**S3**	Provide a network graph of the included studies to enable visualization of the geometry of the treatment network.	Page 8. In the 3.5. **Network meta-analysis** of the **3.Results** section
**Summary of network geometry**	**S4**	Provide a brief overview of characteristics of the treatment network. This may include commentary on the abundance of trials and randomized patients for the different interventions and pairwise comparisons in the network, gaps of evidence in the treatment network, and potential biases reflected by the network structure.	Page 8. In the 3.5. **Network meta-analysis** of the **3.Results** section
Study characteristics	**18**	For each study, present characteristics for which data were extracted (e.g., study size, PICOS, follow-up period) and provide the citations.	Page 6. In the 3.2. **Study characteristics** of the **3.Results** section
Risk of bias within studies	**19**	Present data on risk of bias of each study and, if available, any outcome level assessment.	Page 7. In the 3.3. **Risk of bias of included studies** of the **3.Results** section
Results of individual studies	**20**	For all outcomes considered (benefits or harms), present, for each study: 1) simple summary data for each intervention group, and 2) effect estimates and confidence intervals. Modified approaches may be needed to deal with information from larger networks.	Page 9. In the 3.5.1. **Primary Outcomes** of the **3.Results** section

Synthesis of results	**21**	Present results of each meta-analysis done, including confidence/credible intervals. In larger networks, authors may focus on comparisons versus a particular comparator (e.g. placebo or standard care), with full findings presented in an appendix. League tables and forest plots may be considered to summarize pairwise comparisons. If additional summary measures were explored (such as treatment rankings), these should also be presented.	Page9-10. In the 3.5.1. **Primary Outcomes and 3.5.2. Secondary outcomes** of the **3.Results** section
**Exploration for inconsistency**	**S5**	Describe results from investigations of inconsistency. This may include such information as measures of model fit to compare consistency and inconsistency models, P values from statistical tests, or summary of inconsistency estimates from different parts of the treatment network	Page 7-8. In the 3.4. **Pairwise meta-analysis** of the **3.Results** section
Risk of bias across studies	**22**	Present results of any assessment of risk of bias across studies for the evidence base being studied.	Page 7. In the 3.3. **Risk of bias of included studies** of the **3.Results** section
Results of additional analyses	**23**	Give results of additional analyses, if done (e.g., sensitivity or subgroup analyses, meta-regression analyses, alternative network geometries studied, alternative choice of prior distributions for Bayesian analyses, and so forth).	Page 12-16. In the 3.4. **Pairwise meta-analysis** of the **3.Results** section
**DISCUSSION** Summary of evidence	**24**	Give results of additional analyses, if done (e.g., sensitivity or subgroup analyses, meta-regression analyses, alternative network geometries studied, alternative choice of prior distributions for Bayesian analyses, and so forth).	Page 13. In the **4.Discussion** section
Strengths and limitation	**25**	Discuss limitations at study and outcome level (e.g., risk of bias), and at review level (e.g., incomplete retrieval of identified research, reporting bias). Comment on the validity of the assumptions, such as transitivity and consistency. Comment on any concerns regarding network geometry (e.g., avoidance of certain comparisons).	Page 15. In the **4.Discussion** section
Conclusions	**26**	Provide a general interpretation of the results in the context of other evidence, and implications for future research.	Page 16. In the **5.Conclusions** section
**FUNDING** Funding	**27**	Describe sources of funding for the systematic review and other support (e.g., supply of data); role of funders for the systematic review. This should also include information regarding whether funding has been received from manufacturers of treatments in the network and/or whether some of the authors are content experts with professional conflicts of interest that could affect use of treatments in the network.	Page 17. In the **Funding** section

Supplement S2

Table S2, The search strategy for the respective database.

Database name	Search strategies
CNKI	TKA=('胃癌' +'胃肿瘤'+'胃恶性肿瘤'+'胃部恶性肿瘤'+'贲门癌'+'残胃癌'+'残胃肿瘤'+'残胃再发癌'+'胃腺癌') AND SU=('注射液'+'注射剂') AND TKA=('SOX' +'奥沙利铂 替吉奥'+'奥沙利铂 爱斯万'+'奥沙利铂 维康达'+'奥沙利铂 s-1' +'化疗') AND FT='随机'
Wanfang	主题:("胃癌" or "胃肿瘤" or "胃恶性肿瘤" or "胃部恶性肿瘤" or"贲门癌"or "残胃癌"or "残胃肿瘤"or "残胃再发癌"or "胃腺癌") and 主题:("注射液" or "注射剂") and 主题:("SOX" or "奥沙利铂 替吉奥" or "奥沙利铂 爱斯万" or "奥沙利铂 维康达" or "奥沙利铂 s-1" or "化疗") and 全部:(随机)
VIP	(M=(胃癌 or 胃肿瘤 or 胃恶性肿瘤 or 胃部恶性肿瘤 or 贲门癌 or 残胃癌 or 残胃肿瘤 or 残胃再发癌 or 胃腺癌) or K=(胃癌 or 胃肿瘤 or 胃恶性肿瘤 or 胃部恶性肿瘤 or 贲门癌 or 残胃癌 or 残胃肿瘤 or 残胃再发癌 or 胃腺癌)) and (M=(注射液 or 注射剂) or K=(注射液 or 注射剂)) and (M=(SOX or 奥沙利铂 替吉奥 or 奥沙利铂 爱斯万 or 奥沙利铂 维康达 or 奥沙利铂 s-1 or 化疗) or K=(SOX or 奥沙利铂 替吉奥 or 奥沙利铂 爱斯万 or 奥沙利铂 维康达 or 奥沙利铂 s-1 or 化疗)) and U=随机
SinoMed	#1 "注射剂"[不加权:扩展]<br>#2 "胃肿瘤"[不加权:扩展]<br>#3 "奥沙利铂"不加权:扩展]<br>#4 "胃肿瘤"[常用字段:智能] OR "胃癌"[常用字段:智能] OR "胃恶性肿瘤"[常用字段:智能] OR "胃部恶性肿瘤"[常用字段:智能] OR "贲门癌"[常用字段:智能] OR "残胃癌"[常用字段:智能] OR "残胃肿瘤"[常用字段:智能] OR "残胃再发癌"[常用字段:智能] OR "胃腺癌"[常用字段:智能]<br>#5 (#4) OR (#2)<br>#6 "注射液"[常用字段:智能] OR "注射剂"[常用字段:智能]<br>#7 (#6) OR (#1)<br>#8 "抗肿瘤联合化疗方案"[不加权:扩展]<br>#9 "替吉奥"[常用字段:智能] OR "爱斯万"[常用字段:智能] OR "维康达"[常用字段:智能] OR "s-1"[常用字段:智能]<br>#10 "奥沙利铂"[常用字段:智能]<br>#11 (#10) OR (#3)<br>#12 (#11) AND (#9)<br>#13 (#12) OR(#8)<br>#14 "SOX" [常用字段:智能]<br>#15 (#14) OR(#13)<br>#16 "随机"[全部字段:智能]
Pubmed	#1: ("Stomach Neoplasms"[Mesh]) OR (Neoplasm, Stomach[Title/Abstract]) OR (Stomach Neoplasm[Title/Abstract]) OR (Neoplasms, Stomach[Title/Abstract]) OR (Gastric Neoplasms[Title/Abstract]) OR (Gastric Neoplasm[Title/Abstract]) OR (Neoplasm,Gastric[Title/Abstract]) OR (Neoplasms, Gastric[Title/Abstract]) OR (Cancer of Stomach[Title/Abstract]) OR (Stomach Cancers[Title/Abstract]) OR (Gastric Cancer[Title/Abstract]) OR (Cancer, Gastric [Title/Abstract]) OR (Cancers, Gastric [Title/Abstract]) OR (Gastric Cancers[Title/Abstract]) OR (Stomach Cancer[Title/Abstract]) OR (Cancer, Stomach [Title/Abstract]) OR (Cancers, Stomach[Title/Abstract]) OR (Cancer of the Stomach[Title/Abstract]) OR (Gastric Cancer, Familial Diffuse[Title/Abstract])<br>#2: ("Injections"[Mesh]) OR (Injection[Title/Abstract]) OR (lnjectables[Title/Abstract]) OR

	(Injectable[Title/Abstract])<br>#3: ("Tegafur"[Mesh]) OR (1-(Tetrahydro-2-furanyl)-5-fluorouracil[Title/Abstract]) OR (N1-(2'-Tetrahydrofuryl)-5-fluorouracil[Title/Abstract]) OR (1-(2-Tetrahydrofury)-5-fluorouracil[Title/Abstract]) OR (5-Fluoro-1-(tetrahydro-2-furanyl)-2,4-pyrimidinedione[Title/Abstract]) OR (FT207[Title/Abstract]) OR (Utefos[Title/Abstract]) OR (Futraful[Title/Abstract]) OR (Sunfural S[Title/Abstract]) OR (Uftoral[Title/Abstract]) OR (Florafur[Title/Abstract]) OR (Fluorofur[Title/Abstract]) OR (Ftorafur[Title/Abstract])<br>#4: (Oxalato-(1,2-cyclohexanediamine)platinum II[Title/Abstract]) OR (Oxaliplatin, (SP-4-2-(1R-trans))-isomer[Title/Abstract]) OR (L-OHP Cpd[Title/Abstract]) OR (Platinum(II)-1,2-cyclohexanediamine Oxalate[Title/Abstract]) OR (1,2-Diaminocyclohexane Platinum Oxalate[Title/Abstract]) OR (1,2 Diaminocyclohexane Platinum Oxalate[Title/Abstract]) OR (1,2-Diamminocyclohexane(trans-1[Title/Abstract])oxolatoplatinum(II)) OR (Platinum(2+[Title/Abstract]) ethanedioate (1R,2R)-1,2-cyclohexanediamine (1:1:1)) OR (Oxaliplatine[Title/Abstract]) OR (Eloxatine[Title/Abstract]) OR (Eloxatin[Title/Abstract]) OR (Oxaliplatin, (SP-4-3-(cis))-isomer[Title/Abstract]) OR (ACT 078[Title/Abstract]) OR (ACT-078[Title/Abstract]) OR (ACT078[Title/Abstract]) OR (Cis-oxalato-(trans-l[Title/Abstract])-1,2-diaminocyclohexane-platinum(II)) OR (Oxaliplatin, (SP-4-2-(1S-trans))-isomer[Title/Abstract]) OR ("Oxaliplatin"[Mesh])<br>#5: ("S1(combination)"[Supplementary Concept]) OR (S1-tegafur-oxonate combination[Title/Abstract) OR (S1-fluoropyrimidine oxonate combination[Title/Abstract]) OR (TS-1 cpd[Title/Abstract]) OR (S-1(combination[Title/Abstract)) OR (S-1 cpd[Title/Abstract]) OR (BMS247616[Title/Abstract])<br>#6:(("Randomized Controlled Trials as Topic"[Mesh]) OR (Randomized Controlled Trial[Publication Type]))<br>#7: #3 AND #4<br>#8: #7 OR #5<br>#9: #8 AND #1 AND #2 AND #6
Cochrane Library	#1 MeSH descriptor: [Stomach Neoplasms] explode all trees<br>#2 MeSH descriptor: [Injections] explode all trees<br>#3 MeSH descriptor: [Tegafur] explode all trees<br>#4 Stomach Neoplasms OR Neoplasm, Stomach OR Stomach Neoplasm OR Neoplasms, Stomach OR Gastric Neoplasms OR Gastric Neoplasm OR Neoplasm,Gastric OR Neoplasms, Gastric OR Cancer of Stomach OR Stomach Cancers OR Gastric Cancer OR Cancer, Gastric OR Cancers, Gastric OR Gastric Cancers OR Stomach Cancer OR Cancer, Stomach OR Cancers, Stomach OR Cancer of the Stomach OR Gastric Cancer, Familial Diffuse<br>#5 Injections OR Injection OR lnjectables OR Injectable<br>#6 Tegafur OR Fluorofur OR Florafur OR Utefos OR Uftoral OR Futraful OR Sunfural S OR Ftorafur OR FT207 OR FT 207 OR FT-207<br>#7 MeSH descriptor: [Oxaliplatin] explode all trees<br>#8 Oxaliplatine OR L-OHP Cpd OR ACT 078 OR ACT-078 OR ACT078 OR Eloxatin OR Eloxatine OR S-1 OR S1 OR TS-1 cpd OR S-1 cpd OR BMS 247616<br>#9 #1 OR #4<br>#10 #2 OR #5<br>#11 #3 OR #6<br>#12 #7 OR # 8<br>#13 #9 AND #10 AND #11 AND #12<br>#14 random*

#15 #9 AND #10 AND #11 AND #12 AND #14

Embase	#1. 'stomach cancer'/exp<br>#2. 'stomach cancer'/exp AND [embase]/lim<br>#3. 'cancer of the cardia':ab,ti OR 'cancer of the gastric antrum':ab,ti OR 'cancer of the gastric body':ab,ti OR 'cancer of the gastric cardia':ab,ti OR 'cancer of the gastric fundus':ab,ti OR 'cancer, stomach':ab,ti OR 'cardia cancer':ab,ti OR 'gastric antral cancer':ab,ti OR 'gastric antrum cancer':ab,ti OR 'gastric body cancer':ab,ti OR 'gastric cancer':ab,ti OR 'gastric cardia cancer':ab,ti OR 'gastric cardiac cancer':ab,ti OR 'gastric malignancies':ab,ti OR 'gastric malignancy':ab,ti OR 'malignancies of the stomach':ab,ti OR 'malignancy of the stomach':ab,ti OR 'malignant gastric neoplasm':ab,ti OR 'malignant gastric tumor':ab,ti OR 'malignant neoplasm of the stomach':ab,ti OR 'malignant neoplasms of the stomach':ab,ti OR 'malignant tumor of the stomach':ab,ti OR 'malignant tumors of the stomach':ab,ti OR 'malignant tumour of the stomach':ab,ti OR 'malignant tumours of the stomach':ab,ti OR 'pyloric cancer':ab,ti OR 'stomach malignancies':ab,ti OR 'stomach malignancy':ab,ti OR 'stomach cancer':ab,ti<br>#4. #2 OR #3<br>#5. 'injection'/exp<br>#6. 'blood vessel injection':ab,ti OR 'gluteal injection':ab,ti OR 'injection solution':ab,ti OR 'injections':ab,ti OR 'percutaneous injection':ab,ti OR 'injection':ab,ti<br>#7. #5 OR #6<br>#8. 'oxaliplatin'/exp<br>#9. (('axiplatin':ab,ti OR 'bendaplatin':ab,ti OR'crisapla':ab,ti OR 'croloxat':ab,ti OR 'dacotin':ab,ti OR 'dacplat':ab,ti OR 'debio 0507':ab,ti OR 'debio0507':ab,ti OR 'ebeoxal':ab,ti OR 'elatofen':ab,ti OR 'eloxatin':ab,ti OR 'eloxatine':ab,ti OR 'elplat':ab,ti OR 'euroxaliplatin':ab,ti OR 'geneplatin':ab,ti OR 'gessedil':ab,ti OR 'heloxatin':ab,ti OR 'jm 83':ab,ti OR 'jm83':ab,ti OR 'liploxa':ab,ti OR 'lipoxal':ab,ti OR 'mbp 426':ab,ti OR 'mbp426':ab,ti OR 'medoxa':ab,ti OR 'nc 4016':ab,ti OR 'nc4016':ab,ti OR 'oksaliplatin':ab,ti OR 'oksaliplatina':ab,ti OR 'oplat':ab,ti OR 'oxalato 1, 2 cyclohexanediamine platinum':ab,ti OR 'oxalatoplatinum':ab,ti OR 'oxalatplatin':ab,ti OR 'oxali':ab,ti OR 'oxalip':ab,ti OR 'oxaliplan':ab,ti OR 'oxaliplatina':ab,ti OR 'oxaliplatine':ab,ti OR 'oxaliplatino':ab,ti OR 'oxaliplatinum':ab,ti OR 'oxaliprol':ab,ti OR 'oxaliquid':ab,ti OR 'oxalisan':ab,ti OR 'oxalisin':ab,ti OR 'oxalizor':ab,ti OR 'oxaltic':ab,ti OR 'oxaltina':ab,ti OR oxamed:ab,ti) AND oxaliplatin:ab,ti OR 'oxaplamyl':ab,ti OR 'oxaviatin':ab,ti OR 'platinum 1, 2 cyclohexanediamine oxalate':ab,ti OR 'platinum 1, 2 diaminocyclohexane oxalate':ab,ti OR 'platinum oxalate 1, 2 diaminocyclohexane':ab,ti OR 'platinum trans':ab,ti) AND oxalato:ab,ti AND '1, 2 diaminocyclohexane':ab,ti OR 'platox':ab,ti OR 'plaxitin':ab,ti OR 'rectoxal':ab,ti OR 'riboxatin':ab,ti OR 'rp 54780':ab,ti OR 'rp54780':ab,ti OR 'sinoxal':ab,ti OR 'sr 96669':ab,ti OR 'sr96669':ab,ti OR 'tio 217':ab,ti OR 'tio217':ab,ti OR 'transplastin':ab,ti OR 'velminox':ab,ti OR 'xaliplat':ab,ti OR 'xoplan':ab,ti OR 'oxaliplatin':ab,ti<br>#10. #8 OR #9<br>#11. 'tegafur'/exp<br>#12. (((((((((((((((((1:ab,ti AND '2 furanidyl':ab,ti AND '5 fluorouracil':ab,ti OR 1:ab,ti) AND '2 tetrahydrofuranyl':ab,ti AND '5 fluorouracil':ab,ti OR 1:ab,ti) AND '2 tetrahydrofuryl':ab,ti AND '5 fluorouracil':ab,ti OR 1:ab,ti) AND 'tetrahydro 2 furanyl':ab,ti AND '5 fluorouracil':ab,ti OR '1 tetrahydrofuryl 5 fluorouracil':ab,ti OR 2:ab,ti) AND '5 fluoro 2, 4 dihydroxypyrimid 1 yl':ab,ti AND tetrahydrofuran:ab,ti OR '5 fluoro 1':ab,ti) AND '2 furanidyl':ab,ti AND uracil:ab,ti OR '5 fluoro 1':ab,ti) AND '2 tetrahydrofuranyl':ab,ti AND uracil:ab,ti OR '5 fluoro 1':ab,ti) AND '2 tetrahydrofuryl':ab,ti AND uracil:ab,ti OR '5 fluoro 1':ab,ti) AND 'tetrahydro 2 furanyl':ab,ti AND '2, 4 pyrimidinedione':ab,ti OR '5 fluoro 1':ab,ti) AND 'tetrahydro 2 furanyl':ab,ti AND '2, 4':ab,ti AND '1h, 3h':ab,ti AND pyrimidinedione:ab,ti OR '5 fluoro 1':ab,ti) AND 'tetrahydro 2 furanyl':ab,ti AND uracil:ab,ti OR '5 fluoro 1':ab,ti) AND 'tetrahydro 2 furyl':ab,ti AND '2, 4 pyrimidinedione':ab,ti OR '5 fluoro 1':ab,ti) AND 'tetrahydro 2 furyl':ab,ti AND uracil:ab,ti OR '5 fluoro 1':ab,ti) AND 'tetrahydrofur 2 yl':ab,ti AND

	uracil:ab,ti OR '5 fluoro 1':ab,ti) AND 'tetrahydrofuran 2 yl':ab,ti AND uracil:ab,ti OR 'citofur':ab,ti OR 'florafur':ab,ti OR 'fluorafur':ab,ti OR 'fluorofur':ab,ti OR 'ft 207':ab,ti OR 'ft207':ab,ti OR 'ftorafur':ab,ti OR 'furflucil':ab,ti OR 'futraful':ab,ti OR 'lifril':ab,ti OR 'mjf 12264':ab,ti OR 'mjf12264':ab,ti OR n:ab,ti) AND '2 tetrahydrofuryl':ab,ti AND '5 fluorouracil':ab,ti OR 'n 2 furanidylfluorouracil':ab,ti OR n1:ab,ti) AND '2 furanidyl':ab,ti AND '5 fluorouracil':ab,ti OR n1:ab,ti) AND '2 tetrahydrofuryl':ab,ti AND '5 fluorouracil':ab,ti OR 'nsc 148958':ab,ti OR 'nsc148958':ab,ti OR 'sf sp':ab,ti OR 'sfsp':ab,ti OR 'sinoflurol':ab,ti OR 'sunfural':ab,ti OR 'tegaful':ab,ti OR 'uracil, 5 fluoro 1':ab,ti) AND 'tetrahydrofuran 2 yl':ab,ti OR 'tegafur':ab,ti<br>#13. #11 OR #12<br>#14. #10 AND #13<br>#15. #4 AND #7 AND #14<br>#16. 'randomized controlled trial'/exp<br>#17. 'controlled trial, randomized':ab,ti OR 'randomised controlled study':ab,ti OR 'randomised controlled trial':ab,ti OR 'randomized controlled study':ab,ti OR 'trial,randomized controlled':ab,ti OR 'randomized controlled trial':ab,ti<br>#18. #16 OR #17<br>#19. #15 AND #18
Web of Science	#1: TS=(Stomach Neoplasms OR Stomach Neoplasm OR Gastric Neoplasms OR Gastric Neoplasm OR Cancer of Stomach OR Stomach Cancers OR Gastric Cancer OR Gastric Cancers OR Stomach Cancer OR Cancer of the Stomach OR Gastric Cancer, Familial Diffuse)<br>#2: TS=(Injections OR Injection OR Injectables OR Injectable)<br>#3: TS=(Tegafur OR 1-(Tetrahydro-2-furanyl)-5-fluorouracil OR N1-(2'-Tetrahydrofuryl)-5-fluorouracil OR 1-(2-Tetrahydrofury)-5-fluorouracil OR 5-Fluoro-1-(tetrahydro-2-furanyl)-2,4-pyrimidinedione OR FT207 OR Utefos OR Futraful OR Sunfural S OR Uftoral OR Florafur OR Fluorofur OR Ftorafur)<br>#4: TS=(Oxalato-(1,2-cyclohexanediamine)platinum II OR Oxaliplatin, (SP-4-2-(1R-trans))-isomer OR L-OHP Cpd OR Platinum(II)-1,2-cyclohexanediamine Oxalate OR 1,2-Diaminocyclohexane Platinum Oxalate OR 1,2 Diaminocyclohexane Platinum Oxalate OR 1,2-Diamminocyclohexane(trans-1)oxolatoplatinum(II) OR Platinum(2+) ethanedioate (1R,2R)-1,2-cyclohexanediamine (1:1:1) OR Oxaliplatine OR Eloxatine OR Eloxatin OR Oxaliplatin, (SP-4-3-(cis))-isomer OR ACT 078 OR ACT-078 OR ACT078 OR Cis-oxalato-(trans-l)-1,2-diaminocyclohexane-platinum(II) OR Oxaliplatin, (SP-4-2-(1S-trans))-isomer OR Oxaliplatin)<br>#5: TS=(S1(combination) OR S1-tegafur-oxonate combination OR S1-fluoropyrimidine oxonate combination OR TS-1 cpd OR S-1(combination) OR S-1 cpd OR BMS247616)<br>#6: TS=(randomized controlled trial OR randomized OR placebo OR random OR randomised)<br>#7: #3 AND #4<br>#8: #7 OR #5<br>#9: #8 AND #1 AND #2 AND #6

CNKI: China National Knowledge Infrastructure; SinoMed: the Chinese Biomedical Literature Database; WanFang: the WanFang Database; VIP: the Chinese Scientific Journals Full-Text Database; Embase Database: Excerpta Medica Database; WOS Database: Web of Science Database.

Supplement S3

Table S3, Detailed information on included CHIs.

**Injection Name**	**Execution standards and approval numbers**	**Source**	**Composition**	**Indications**	**Adverse reactions**	**Chemical analysis Reported (Y/N)**
Aidi injections	National Food and Drug Administration National Drug Standard, WS3-B-3809-99-2002. National Pharmaceutical Standard, Z52020236.	Guizhou Yibai Pharmaceutical Co., Ltd.	*Panax ginseng C.A.Mey.*[Araliaceae] *Eleutherococcus senticosus (Rupr. & Maxim.) Maxim.* [Araliaceae] *Astragalus membranaceus (Fisch.) Bunge.*[Fabaceae] *Harmonia axyridis (Pallas)* [Coccinellidae]	Primary liver cancer, lung cancer, rectal cancer,malignant lymphoma, gynecological malignant tumor.	When using this product for the first time, patients may occasionally experience mild reactions such as flushing, hives, and fever, while only a very small number of patients may experience more severe reactions such as palpitations, chest tightness, and nausea.	N
Shenfu injections	National Food and Drug Administration National Drug Standard, WB3-B-3427-98-2013. National Pharmaceutical Standard, Z20043117.	Sichun Yaan Pharmaceutical Co., Ltd.	*Panax ginseng C.A.Mey.* [Araliaceae] *Aconitum carmichaeli Debeaux*[Ranunculaceae]	Infectious, hemorrhagic, and fluid loss shock may cause palpitations, asthma, coughing, stomach pain, diarrhea, and joint pain.	Occasionally, in clinical settings, tachycardia, anaphylaxis, rash, dizziness, headache, hiccup, tremor, dyspnea, nausea, visual abnormalities, liver dysfunction, and urinary retention have been reported as possible side effects.	N
Shenqifuzhen injections	National Food and Drug Administration National Drug Standard, WS3-387（Z-50）- 2003（Z）. National Pharmaceutical Standard, Z19990065.	Guangdong Lizhu Group Limin Pharmaceutical Factory.	*Codonopsis pilosula (Franch.) Nannf.* [Campanulaceae] *Astragalus membranaceus (Fisch.) Bunge* [Fabaceae] *Sodium Chloride* *Sodium bisulfite* *Sodium edetate*	Adjuvant treatment for lung cancer，gastric cancer with symptoms of mental fatigue caused by deficiency of lung and spleen, lack of qi and energy, lazy speech, spontaneous sweating, anddizziness	1. Patients without Qi deficiency syndrome may experience mild bleeding after taking the medication. 2. Some patients may experience low-grade fever, stomatitis, and fatigue after taking the medicine. 3. Occasional side effects may include rash, chills, high fever, vomiting, chest tightness, palpitations, and so on.	N

Fufang kushen injections	National Food and Drug Administration National Drug Standard,WS_3-B-2752-97-2014. National Pharmaceutical Standard, Z14021231.	Shanxi Zhendong Pharmaceutical Co., Ltd.	*Sophora flavescens Aiton*[Fabaceae]<br>*Smilax glabra Roxb.*[Smilacaceae]<br>*Polysorbate 80*<br>*Sodium hydroxide*<br>*Sodium hydroxide*<br>*Acetic acid*	For cancer pain and bleeding.	This product has minimal systemic toxicity and side effects and may cause mild local irritation but is well-absorbed.	N
Kangai injections	National Food and Drug Administration National Drug Standard: WS-11222 (ZD-1222) -2002-2012Z National Pharmaceutical Standard Z20026868;	Changbaishan Pharmaceutical Co., Ltd.	*Astragalus membranaceus (Fisch.) Bunge*[Fabaceae]<br>*Panax ginseng C.A.Mey.* [Araliaceae]<br>*Sophora flavescens Aiton*[Fabaceae]	Replenish qi and strengthen the body's immune function. Used for primary liver cancer, lung cancer, rectal cancer, malignant lymphoma, gynecological malignant tumor; Leukopenia and leukopenia caused by various reasons. Treatment of chronic hepatitis B.	Adverse reactions to this product are extremely rare, and during clinical use, allergic reactions are seldom reported.	N
Kanglaite injections	National Food and Drug Administration National Drug Standard, WS_3-301 (Z-038) -2006 (Z) -2013. National Pharmaceutical Standard, Z10970091.	Zhejiang Kanglaite Pharmaceutical Co., Ltd.	*Coix lacryma-jobi L.*[Poaceae]<br>*Glycerol*<br>*Glycine max (L.) Merr.* [Fabaceae]	Indicated for primary non-small cell lung cancer and primary liver cancer accompanied by deficiency of both Qi and Yin as well as deficiency of spleen and dampness.	Occasionally, clinics may observe lipid allergy with symptoms such as elevated body temperature, mild nausea, and chills. However, most of these symptoms usually disappear naturally after 3 to 5 days of use. Mild phlebitis may also occur occasionally.	N

Huachansu Injections	National Food and Drug Administration National Drug Standard, WS_3-B-3045-98. National Pharmaceutical Standard, Z3402027.	Anhui China Resources Jinchan Pharmaceutical Co., Ltd.	*Bufo gargarizans* [Bufonidae] *Mannitol* *Polyethylene glycol*	It is used for middle and late stage tumors, chronic hepatitis B and other diseases.	If the dosage administered to individual patients is too high or the interval between two doses is less than 6 to 8 hours, cold and fever may occur about 30 minutes after drug administration. A few patients may experience local irritation or phlebitis after a long intravenous drip resulting in a slow drip rate. In very rare cases, patients may also experience urticaria, dermatitis, and other allergic reactions.	N
Xiaoaiping injections	National Pharmaceutical Standard, Z34020273.	Nanjing Shenghe Pharmaceutical Co., Ltd	*Aristolochia debilis Siebold & Zucc.*[Aristolochiaceae]	This medication can be used to treat various types of cancer, including esophageal cancer, gastric cancer, lung cancer, and liver cancer. It can also be used in combination with radiotherapy and chemotherapy for a more comprehensive treatment approach.	Not clear yet.	N
Huangqi injections	National Pharmaceutical Standard, Z32021257.	Shanghai Xinya Pharmaceutical Gaoyou Co., Ltd	*Astragalus membranaceus (Fisch.) Bunge*[Fabaceae] *Mannitol* *Polyethylene Glycol*	This medication is used to treat viral myocarditis with symptoms of heart qi deficiency, blood stasis, and heart insufficiency. It is also used to treat hepatitis accompanied by spleen deficiency and dampness.	According to literature reports, the adverse reactions of this product are as follows:<br>1. Allergic reactions: common drug fever, drug rash, and red and swollen injection site; serious adverse reactions such as acute allergic reaction and anaphylactic shock are rare.	N

2. Respiratory system: common laryngeal edema, dyspnea, asthma, and chest tightness.<br>3. Circulatory system: occasionally delayed-type venous inflammation with low blood pressure; rarely rapid atrial fibrillation.<br>4. Digestive system: occasional liver function damage, vomiting, and diarrhea.

Composition(from:http://mpns.kew.org/mpns-portal/, http://www.plantsoftheworldonline.org, and https://www.catalogueoflife.org/).

Supplement S4

The forest plot Pairwise Meta-Analysis of outcomes

Study	Experimental Events	Experimental Total	Control Events	Control Total	RR	95%-CI	Weight
subgroup = A vs.I							
Fan CM 2011	11	23	10	28	1.34	[0.70; 2.58]	1.2%
Li YQ 2013	12	30	9	28	1.24	[0.62; 2.49]	1.0%
Rao JJ 2018	23	45	13	45	1.77	[1.03; 3.04]	1.7%
Wang R 2018	25	39	16	39	1.56	[1.00; 2.43]	2.5%
Xu JI 2017	26	47	20	47	1.30	[0.85; 1.98]	2.8%
Yang Q 2021	17	30	8	30	2.12	[1.09; 4.16]	1.1%
Zhao HB 2020	48	75	26	75	1.85	[1.30; 2.63]	4.0%
Wang J 2013	34	50	25	50	1.36	[0.97; 1.90]	4.4%
Chen YX 2020	41	45	32	45	1.28	[1.04; 1.58]	11.6%
Li RQ 2020	23	32	14	30	1.54	[0.99; 2.39]	2.6%
Random effects model		416		417	1.44	[1.27; 1.63]	32.9%
Heterogeneity: $I^2 = 0\%$, $\tau^2 = 0$, $p = 0.766$							
subgroup = B vs.I							
Xie YG 2014	17	40	9	37	1.75	[0.89; 3.42]	1.1%
subgroup = C vs.I							
Chen XT 2020	43	60	30	60	1.43	[1.06; 1.93]	5.6%
Ruan XJ 2012	14	27	12	27	1.17	[0.67; 2.04]	1.6%
Tang Xf 2017	23	31	13	30	1.71	[1.08; 2.71]	2.4%
Wang P 2019	32	70	31	70	1.03	[0.72; 1.49]	3.7%
Yang ZY 2014	26	46	14	40	1.61	[0.99; 2.64]	2.1%
Zhang WH 2012	9	20	4	10	1.12	[0.46; 2.77]	0.6%
Jiang J 2015	8	15	5	15	1.60	[0.68; 3.77]	0.7%
Sun GZ 2013	14	20	11	18	1.15	[0.72; 1.83]	2.3%
Song P 2019	17	33	14	33	1.21	[0.72; 2.04]	1.9%
Shi ZW 2020	24	41	22	41	1.09	[0.74; 1.60]	3.4%
Random effects model		363		344	1.28	[1.11; 1.48]	24.1%
Heterogeneity: $I^2 = 0\%$, $\tau^2 = 0$, $p = 0.775$							
subgroup = D vs.I							
Chang ZG 2021	31	53	20	53	1.55	[1.03; 2.34]	2.9%
H B 2022	21	40	10	35	1.84	[1.01; 3.35]	1.4%
Xue TL 2023	26	49	24	49	1.08	[0.73; 1.60]	3.3%
Ma YK 2017	17	30	9	30	1.89	[1.01; 3.55]	1.3%
Random effects model		172		167	1.46	[1.11; 1.92]	8.9%
Heterogeneity: $I^2 = 14.9\%$, $\tau^2 = 0.02$, $p = 0.318$							
subgroup = E vs.I							
Hu Q 2017	13	18	7	18	1.86	[0.97; 3.54]	1.2%
Si LI 2021	13	39	11	39	1.18	[0.61; 2.31]	1.1%
Xie JF 2016	25	41	15	40	1.63	[1.02; 2.60]	2.3%
Pang YP 2017	21	42	12	42	1.75	[0.99; 3.08]	1.6%
Wu Y 2019	18	32	11	28	1.43	[0.82; 2.49]	1.6%
Random effects model		172		167	1.57	[1.22; 2.02]	7.8%
Heterogeneity: $I^2 = 0\%$, $\tau^2 = 0$, $p = 0.875$							
subgroup = F vs.I							
Gao NN 2018	14	24	13	24	1.08	[0.65; 1.78]	2.0%
Shen G 2017	28	54	22	50	1.18	[0.79; 1.77]	3.0%
Yang QW 2021	22	37	17	34	1.19	[0.77; 1.83]	2.7%
Yao XJ 2015	18	27	8	22	1.83	[0.99; 3.39]	1.3%
Random effects model		142		130	1.24	[0.98; 1.56]	9.1%
Heterogeneity: $I^2 = 0\%$, $\tau^2 = 0$, $p = 0.580$							
subgroup = G vs.I							
Dou SS 2021	24	36	15	36	1.60	[1.02; 2.51]	2.5%
Dong L 2019	22	32	13	32	1.69	[1.05; 2.73]	2.2%
Random effects model		68		68	1.64	[1.18; 2.28]	4.6%
Heterogeneity: $I^2 = 0\%$, $\tau^2 = 0$, $p = 0.867$							
subgroup = H vs.I							
Ruan XJ 2021	19	42	17	42	1.12	[0.68; 1.83]	2.0%
Liu W 2017	32	48	30	48	1.07	[0.79; 1.44]	5.7%
Xiong L 2015	9	32	6	32	1.50	[0.60; 3.72]	0.6%
Ma YJ 2015	12	23	11	23	1.09	[0.61; 1.95]	1.5%
Gao M 2017	19	33	12	33	1.58	[0.92; 2.71]	1.7%
Random effects model		178		178	1.16	[0.95; 1.43]	11.5%
Heterogeneity: $I^2 = 0\%$, $\tau^2 = 0$, $p = 0.743$							
Random effects model		1551		1508	1.37	[1.27; 1.47]	100.0%

Heterogeneity: $I^2 = 0.0\%$, $\tau^2 = 0$, $p = 0.933$
Test for overall effect: $z = 8.680$ ($p < 0.001$)
Test for subgroup differences: $\chi^2_7 = 7.496$, df = 7 ($p = 0.379$)

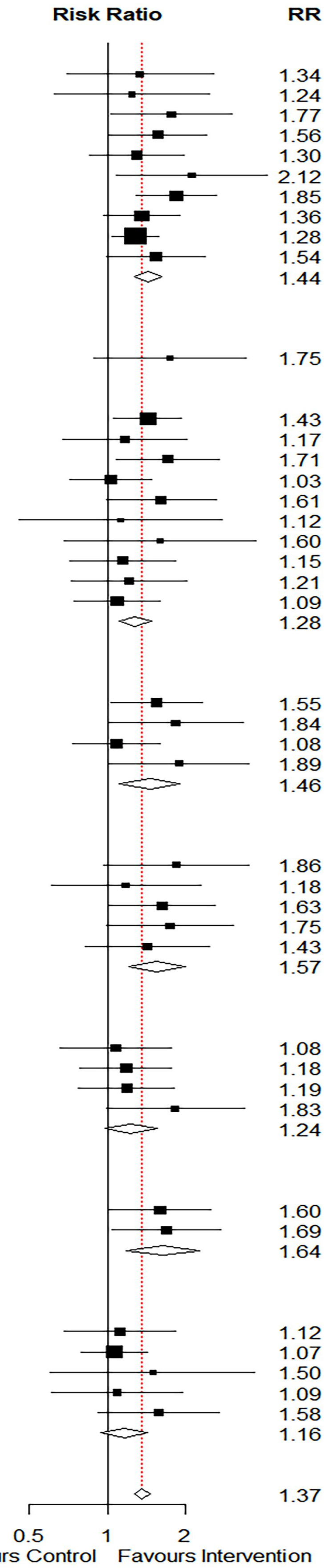

(1)

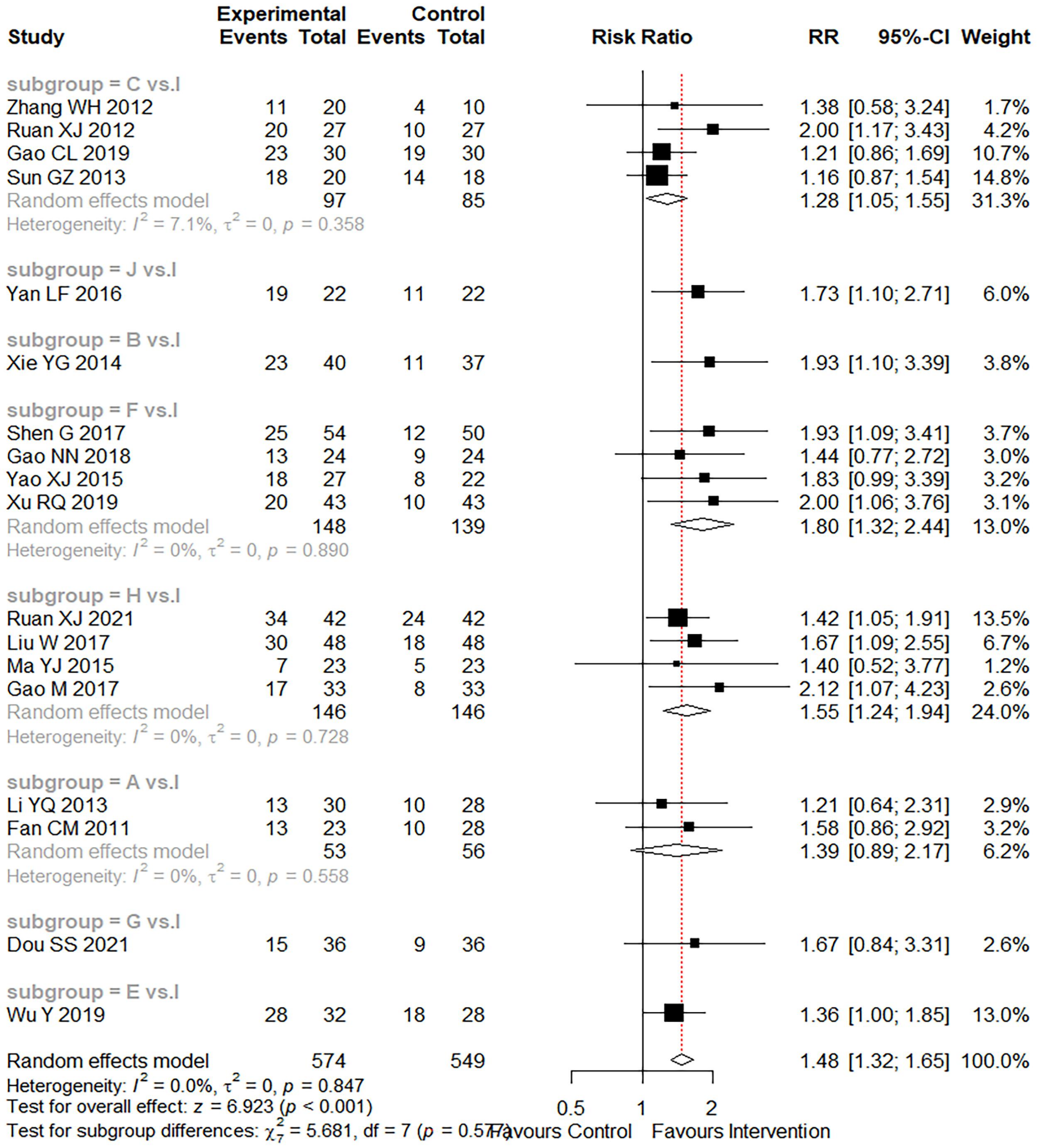
Study
Experimental Events Total
Control Events Total
Risk Ratio
RR 95%-CI Weight
subgroup = C vs.I
Zhang WH 2012 11 20 4 10 1.38 [0.58; 3.24] 1.7%
Ruan XJ 2012 20 27 10 27 2.00 [1.17; 3.43] 4.2%
Gao CL 2019 23 30 19 30 1.21 [0.86; 1.69] 10.7%
Sun GZ 2013 18 20 14 18 1.16 [0.87; 1.54] 14.8%
Random effects model 97 85 1.28 [1.05; 1.55] 31.3%
Heterogeneity: I^2 = 7.1%, τ^2 = 0, p = 0.358
subgroup = J vs.I
Yan LF 2016 19 22 11 22 1.73 [1.10; 2.71] 6.0%
subgroup = B vs.I
Xie YG 2014 23 40 11 37 1.93 [1.10; 3.39] 3.8%
subgroup = F vs.I
Shen G 2017 25 54 12 50 1.93 [1.09; 3.41] 3.7%
Gao NN 2018 13 24 9 24 1.44 [0.77; 2.72] 3.0%
Yao XJ 2015 18 27 8 22 1.83 [0.99; 3.39] 3.2%
Xu RQ 2019 20 43 10 43 2.00 [1.06; 3.76] 3.1%
Random effects model 148 139 1.80 [1.32; 2.44] 13.0%
Heterogeneity: I^2 = 0%, τ^2 = 0, p = 0.890
subgroup = H vs.I
Ruan XJ 2021 34 42 24 42 1.42 [1.05; 1.91] 13.5%
Liu W 2017 30 48 18 48 1.67 [1.09; 2.55] 6.7%
Ma YJ 2015 7 23 5 23 1.40 [0.52; 3.77] 1.2%
Gao M 2017 17 33 8 33 2.12 [1.07; 4.23] 2.6%
Random effects model 146 146 1.55 [1.24; 1.94] 24.0%
Heterogeneity: I^2 = 0%, τ^2 = 0, p = 0.728
subgroup = A vs.I
Li YQ 2013 13 30 10 28 1.21 [0.64; 2.31] 2.9%
Fan CM 2011 13 23 10 28 1.58 [0.86; 2.92] 3.2%
Random effects model 53 56 1.39 [0.89; 2.17] 6.2%
Heterogeneity: I^2 = 0%, τ^2 = 0, p = 0.558
subgroup = G vs.I
Dou SS 2021 15 36 9 36 1.67 [0.84; 3.31] 2.6%
subgroup = E vs.I
Wu Y 2019 28 32 18 28 1.36 [1.00; 1.85] 13.0%
Random effects model 574 549 1.48 [1.32; 1.65] 100.0%
Heterogeneity: I^2 = 0.0%, τ^2 = 0, p = 0.847
Test for overall effect: z = 6.923 (p < 0.001)
Test for subgroup differences: χ^2_7 = 5.681, df = 7 (p = 0.57)
0.5 1 2
Favours Control Favours Intervention

(2)

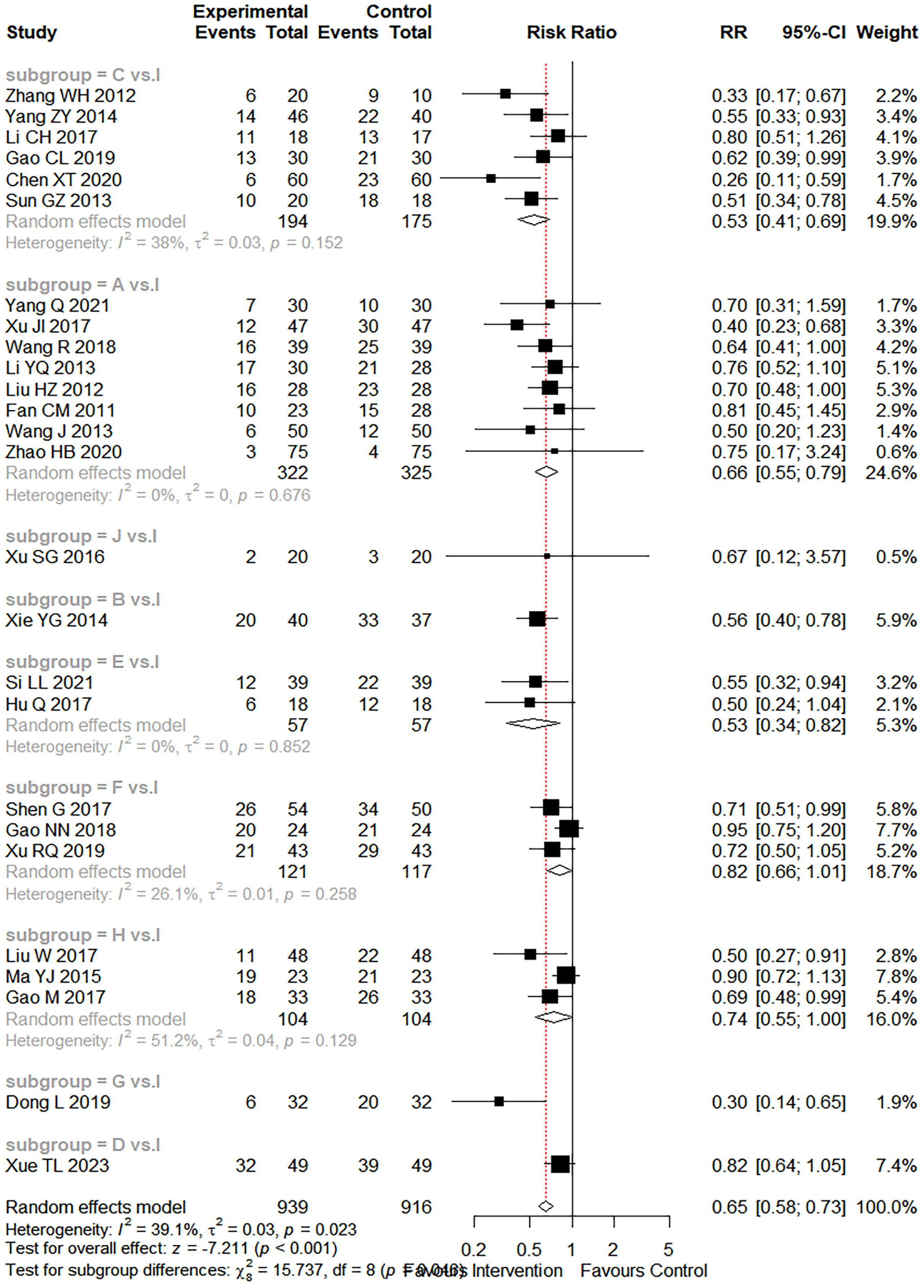

Experimental Control
Study Events Total Events Total Risk Ratio RR 95%-CI Weight
subgroup = C vs.I
Zhang WH 2012 6 20 9 10 0.33 [0.17; 0.67] 2.2%
Yang ZY 2014 14 46 22 40 0.55 [0.33; 0.93] 3.4%
Li CH 2017 11 18 13 17 0.80 [0.51; 1.26] 4.1%
Gao CL 2019 13 30 21 30 0.62 [0.39; 0.99] 3.9%
Chen XT 2020 6 60 23 60 0.26 [0.11; 0.59] 1.7%
Sun GZ 2013 10 20 18 18 0.51 [0.34; 0.78] 4.5%
Random effects model 194 175 0.53 [0.41; 0.69] 19.9%
Heterogeneity: I^2 = 38%, τ^2 = 0.03, p = 0.152
subgroup = A vs.I
Yang Q 2021 7 30 10 30 0.70 [0.31; 1.59] 1.7%
Xu JI 2017 12 47 30 47 0.40 [0.23; 0.68] 3.3%
Wang R 2018 16 39 25 39 0.64 [0.41; 1.00] 4.2%
Li YQ 2013 17 30 21 28 0.76 [0.52; 1.10] 5.1%
Liu HZ 2012 16 28 23 28 0.70 [0.48; 1.00] 5.3%
Fan CM 2011 10 23 15 28 0.81 [0.45; 1.45] 2.9%
Wang J 2013 6 50 12 50 0.50 [0.20; 1.23] 1.4%
Zhao HB 2020 3 75 4 75 0.75 [0.17; 3.24] 0.6%
Random effects model 322 325 0.66 [0.55; 0.79] 24.6%
Heterogeneity: I^2 = 0%, τ^2 = 0, p = 0.676
subgroup = J vs.I
Xu SG 2016 2 20 3 20 0.67 [0.12; 3.57] 0.5%
subgroup = B vs.I
Xie YG 2014 20 40 33 37 0.56 [0.40; 0.78] 5.9%
subgroup = E vs.I
Si LL 2021 12 39 22 39 0.55 [0.32; 0.94] 3.2%
Hu Q 2017 6 18 12 18 0.50 [0.24; 1.04] 2.1%
Random effects model 57 57 0.53 [0.34; 0.82] 5.3%
Heterogeneity: I^2 = 0%, τ^2 = 0, p = 0.852
subgroup = F vs.I
Shen G 2017 26 54 34 50 0.71 [0.51; 0.99] 5.8%
Gao NN 2018 20 24 21 24 0.95 [0.75; 1.20] 7.7%
Xu RQ 2019 21 43 29 43 0.72 [0.50; 1.05] 5.2%
Random effects model 121 117 0.82 [0.66; 1.01] 18.7%
Heterogeneity: I^2 = 26.1%, τ^2 = 0.01, p = 0.258
subgroup = H vs.I
Liu W 2017 11 48 22 48 0.50 [0.27; 0.91] 2.8%
Ma YJ 2015 19 23 21 23 0.90 [0.72; 1.13] 7.8%
Gao M 2017 18 33 26 33 0.69 [0.48; 0.99] 5.4%
Random effects model 104 104 0.74 [0.55; 1.00] 16.0%
Heterogeneity: I^2 = 51.2%, τ^2 = 0.04, p = 0.129
subgroup = G vs.I
Dong L 2019 6 32 20 32 0.30 [0.14; 0.65] 1.9%
subgroup = D vs.I
Xue TL 2023 32 49 39 49 0.82 [0.64; 1.05] 7.4%
Random effects model 939 916 0.65 [0.58; 0.73] 100.0%
Heterogeneity: I^2 = 39.1%, τ^2 = 0.03, p = 0.023
Test for overall effect: z = -7.211 (p < 0.001)
Test for subgroup differences: χ^2_8 = 15.737, df = 8 (p [illegible])
0.2 0.5 1 2 5
Favours Intervention Favours Control

(3)

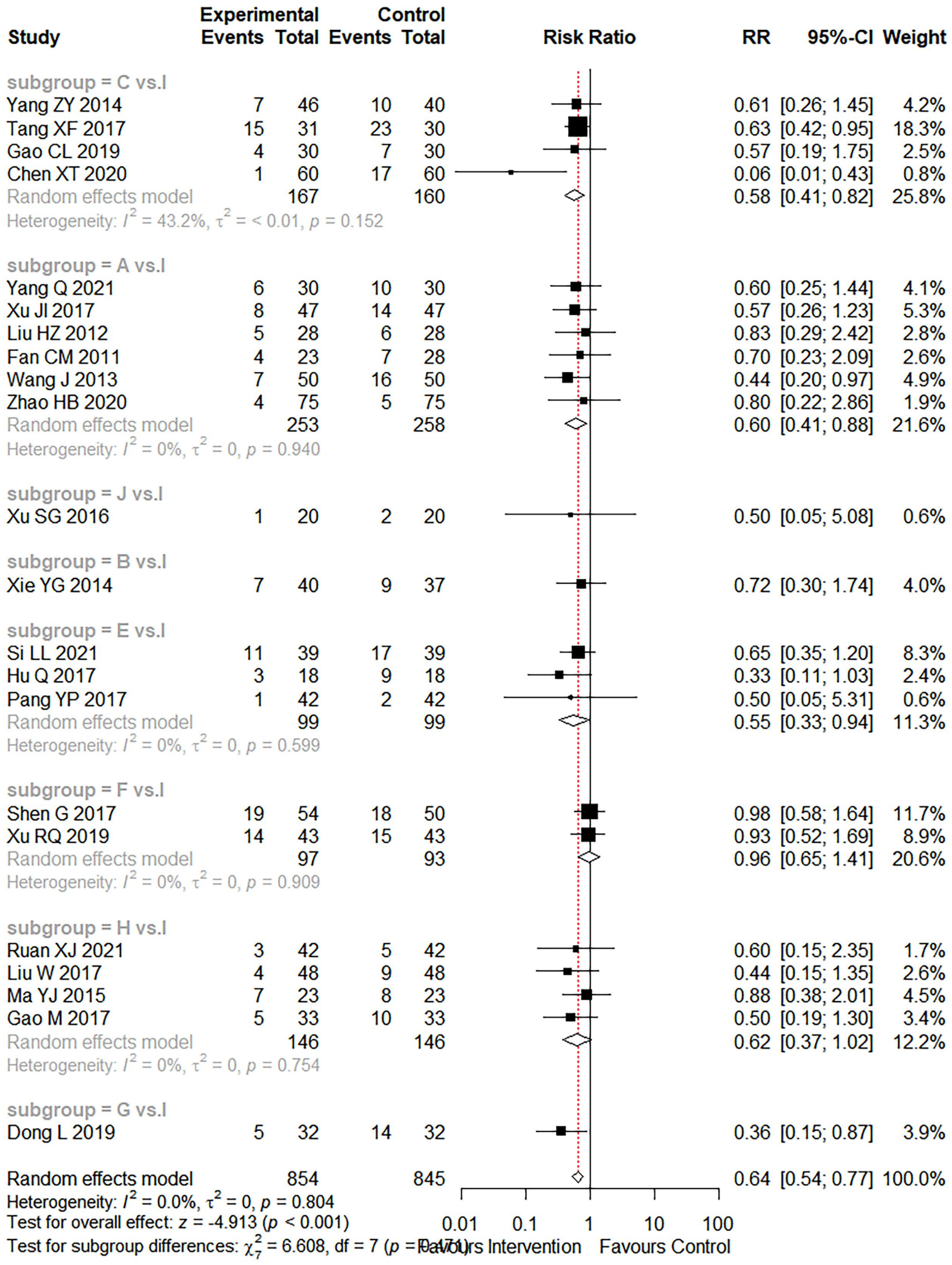

Experimental
Control
Study
Events Total Events Total
Risk Ratio
RR 95%-CI Weight
subgroup = C vs.I
Yang ZY 2014 7 46 10 40 0.61 [0.26; 1.45] 4.2%
Tang XF 2017 15 31 23 30 0.63 [0.42; 0.95] 18.3%
Gao CL 2019 4 30 7 30 0.57 [0.19; 1.75] 2.5%
Chen XT 2020 1 60 17 60 0.06 [0.01; 0.43] 0.8%
Random effects model 167 160 0.58 [0.41; 0.82] 25.8%
Heterogeneity: I^2 = 43.2%, τ^2 = < 0.01, p = 0.152
subgroup = A vs.I
Yang Q 2021 6 30 10 30 0.60 [0.25; 1.44] 4.1%
Xu JI 2017 8 47 14 47 0.57 [0.26; 1.23] 5.3%
Liu HZ 2012 5 28 6 28 0.83 [0.29; 2.42] 2.8%
Fan CM 2011 4 23 7 28 0.70 [0.23; 2.09] 2.6%
Wang J 2013 7 50 16 50 0.44 [0.20; 0.97] 4.9%
Zhao HB 2020 4 75 5 75 0.80 [0.22; 2.86] 1.9%
Random effects model 253 258 0.60 [0.41; 0.88] 21.6%
Heterogeneity: I^2 = 0%, τ^2 = 0, p = 0.940
subgroup = J vs.I
Xu SG 2016 1 20 2 20 0.50 [0.05; 5.08] 0.6%
subgroup = B vs.I
Xie YG 2014 7 40 9 37 0.72 [0.30; 1.74] 4.0%
subgroup = E vs.I
Si LL 2021 11 39 17 39 0.65 [0.35; 1.20] 8.3%
Hu Q 2017 3 18 9 18 0.33 [0.11; 1.03] 2.4%
Pang YP 2017 1 42 2 42 0.50 [0.05; 5.31] 0.6%
Random effects model 99 99 0.55 [0.33; 0.94] 11.3%
Heterogeneity: I^2 = 0%, τ^2 = 0, p = 0.599
subgroup = F vs.I
Shen G 2017 19 54 18 50 0.98 [0.58; 1.64] 11.7%
Xu RQ 2019 14 43 15 43 0.93 [0.52; 1.69] 8.9%
Random effects model 97 93 0.96 [0.65; 1.41] 20.6%
Heterogeneity: I^2 = 0%, τ^2 = 0, p = 0.909
subgroup = H vs.I
Ruan XJ 2021 3 42 5 42 0.60 [0.15; 2.35] 1.7%
Liu W 2017 4 48 9 48 0.44 [0.15; 1.35] 2.6%
Ma YJ 2015 7 23 8 23 0.88 [0.38; 2.01] 4.5%
Gao M 2017 5 33 10 33 0.50 [0.19; 1.30] 3.4%
Random effects model 146 146 0.62 [0.37; 1.02] 12.2%
Heterogeneity: I^2 = 0%, τ^2 = 0, p = 0.754
subgroup = G vs.I
Dong L 2019 5 32 14 32 0.36 [0.15; 0.87] 3.9%
Random effects model 854 845 0.64 [0.54; 0.77] 100.0%
Heterogeneity: I^2 = 0.0%, τ^2 = 0, p = 0.804
Test for overall effect: z = -4.913 (p < 0.001)
Test for subgroup differences: χ^2_7 = 6.608, df = 7 (p = 0.471)
0.01 0.1 1 10 100
Favours Intervention Favours Control

(4)

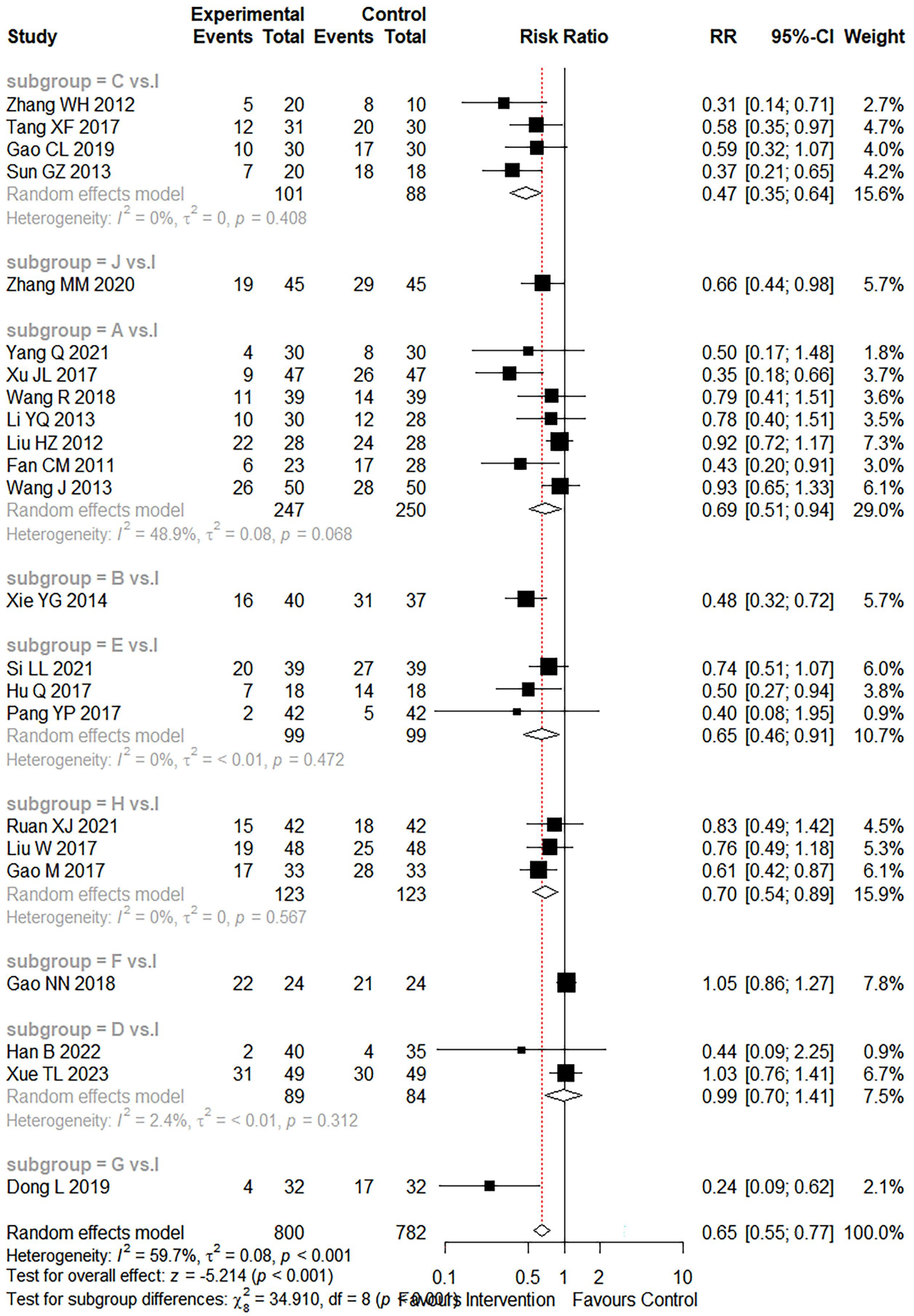

Study
Experimental Events Total
Control Events Total
Risk Ratio
RR 95%-CI Weight
subgroup = C vs.I
Zhang WH 2012 5 20 8 10 0.31 [0.14; 0.71] 2.7%
Tang XF 2017 12 31 20 30 0.58 [0.35; 0.97] 4.7%
Gao CL 2019 10 30 17 30 0.59 [0.32; 1.07] 4.0%
Sun GZ 2013 7 20 18 18 0.37 [0.21; 0.65] 4.2%
Random effects model 101 88 0.47 [0.35; 0.64] 15.6%
Heterogeneity: I^2 = 0%, τ^2 = 0, p = 0.408
subgroup = J vs.I
Zhang MM 2020 19 45 29 45 0.66 [0.44; 0.98] 5.7%
subgroup = A vs.I
Yang Q 2021 4 30 8 30 0.50 [0.17; 1.48] 1.8%
Xu JL 2017 9 47 26 47 0.35 [0.18; 0.66] 3.7%
Wang R 2018 11 39 14 39 0.79 [0.41; 1.51] 3.6%
Li YQ 2013 10 30 12 28 0.78 [0.40; 1.51] 3.5%
Liu HZ 2012 22 28 24 28 0.92 [0.72; 1.17] 7.3%
Fan CM 2011 6 23 17 28 0.43 [0.20; 0.91] 3.0%
Wang J 2013 26 50 28 50 0.93 [0.65; 1.33] 6.1%
Random effects model 247 250 0.69 [0.51; 0.94] 29.0%
Heterogeneity: I^2 = 48.9%, τ^2 = 0.08, p = 0.068
subgroup = B vs.I
Xie YG 2014 16 40 31 37 0.48 [0.32; 0.72] 5.7%
subgroup = E vs.I
Si LL 2021 20 39 27 39 0.74 [0.51; 1.07] 6.0%
Hu Q 2017 7 18 14 18 0.50 [0.27; 0.94] 3.8%
Pang YP 2017 2 42 5 42 0.40 [0.08; 1.95] 0.9%
Random effects model 99 99 0.65 [0.46; 0.91] 10.7%
Heterogeneity: I^2 = 0%, τ^2 = < 0.01, p = 0.472
subgroup = H vs.I
Ruan XJ 2021 15 42 18 42 0.83 [0.49; 1.42] 4.5%
Liu W 2017 19 48 25 48 0.76 [0.49; 1.18] 5.3%
Gao M 2017 17 33 28 33 0.61 [0.42; 0.87] 6.1%
Random effects model 123 123 0.70 [0.54; 0.89] 15.9%
Heterogeneity: I^2 = 0%, τ^2 = 0, p = 0.567
subgroup = F vs.I
Gao NN 2018 22 24 21 24 1.05 [0.86; 1.27] 7.8%
subgroup = D vs.I
Han B 2022 2 40 4 35 0.44 [0.09; 2.25] 0.9%
Xue TL 2023 31 49 30 49 1.03 [0.76; 1.41] 6.7%
Random effects model 89 84 0.99 [0.70; 1.41] 7.5%
Heterogeneity: I^2 = 2.4%, τ^2 = < 0.01, p = 0.312
subgroup = G vs.I
Dong L 2019 4 32 17 32 0.24 [0.09; 0.62] 2.1%
Random effects model 800 782 0.65 [0.55; 0.77] 100.0%
Heterogeneity: I^2 = 59.7%, τ^2 = 0.08, p < 0.001
Test for overall effect: z = -5.214 (p < 0.001)
Test for subgroup differences: χ^2_8 = 34.910, df = 8 (p < 0.001)
0.1 0.5 1 2 10
Favours Intervention Favours Control

(5)

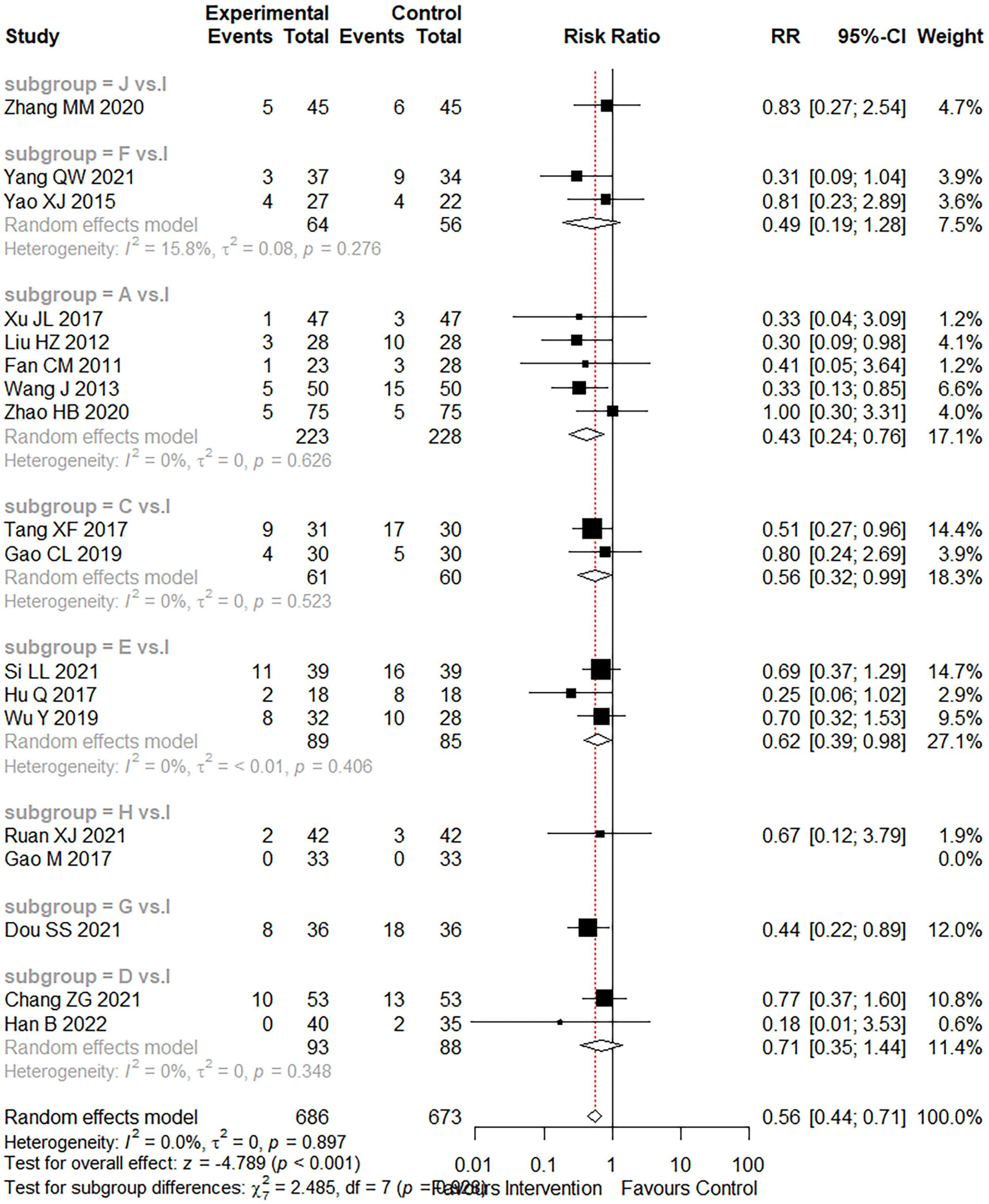
Study
Experimental Events Total
Control Events Total
Risk Ratio
RR 95%-CI Weight
subgroup = J vs.I
Zhang MM 2020 5 45 6 45 0.83 [0.27; 2.54] 4.7%
subgroup = F vs.I
Yang QW 2021 3 37 9 34 0.31 [0.09; 1.04] 3.9%
Yao XJ 2015 4 27 4 22 0.81 [0.23; 2.89] 3.6%
Random effects model 64 56 0.49 [0.19; 1.28] 7.5%
Heterogeneity: I^2 = 15.8%, τ^2 = 0.08, p = 0.276
subgroup = A vs.I
Xu JL 2017 1 47 3 47 0.33 [0.04; 3.09] 1.2%
Liu HZ 2012 3 28 10 28 0.30 [0.09; 0.98] 4.1%
Fan CM 2011 1 23 3 28 0.41 [0.05; 3.64] 1.2%
Wang J 2013 5 50 15 50 0.33 [0.13; 0.85] 6.6%
Zhao HB 2020 5 75 5 75 1.00 [0.30; 3.31] 4.0%
Random effects model 223 228 0.43 [0.24; 0.76] 17.1%
Heterogeneity: I^2 = 0%, τ^2 = 0, p = 0.626
subgroup = C vs.I
Tang XF 2017 9 31 17 30 0.51 [0.27; 0.96] 14.4%
Gao CL 2019 4 30 5 30 0.80 [0.24; 2.69] 3.9%
Random effects model 61 60 0.56 [0.32; 0.99] 18.3%
Heterogeneity: I^2 = 0%, τ^2 = 0, p = 0.523
subgroup = E vs.I
Si LL 2021 11 39 16 39 0.69 [0.37; 1.29] 14.7%
Hu Q 2017 2 18 8 18 0.25 [0.06; 1.02] 2.9%
Wu Y 2019 8 32 10 28 0.70 [0.32; 1.53] 9.5%
Random effects model 89 85 0.62 [0.39; 0.98] 27.1%
Heterogeneity: I^2 = 0%, τ^2 = < 0.01, p = 0.406
subgroup = H vs.I
Ruan XJ 2021 2 42 3 42 0.67 [0.12; 3.79] 1.9%
Gao M 2017 0 33 0 33 0.0%
subgroup = G vs.I
Dou SS 2021 8 36 18 36 0.44 [0.22; 0.89] 12.0%
subgroup = D vs.I
Chang ZG 2021 10 53 13 53 0.77 [0.37; 1.60] 10.8%
Han B 2022 0 40 2 35 0.18 [0.01; 3.53] 0.6%
Random effects model 93 88 0.71 [0.35; 1.44] 11.4%
Heterogeneity: I^2 = 0%, τ^2 = 0, p = 0.348
Random effects model 686 673 0.56 [0.44; 0.71] 100.0%
Heterogeneity: I^2 = 0.0%, τ^2 = 0, p = 0.897
Test for overall effect: z = -4.789 (p < 0.001)
Test for subgroup differences: χ^2_7 = 2.485, df = 7 (p = 0.928)
0.01 0.1 1 10 100
Favours Intervention Favours Control

(6)

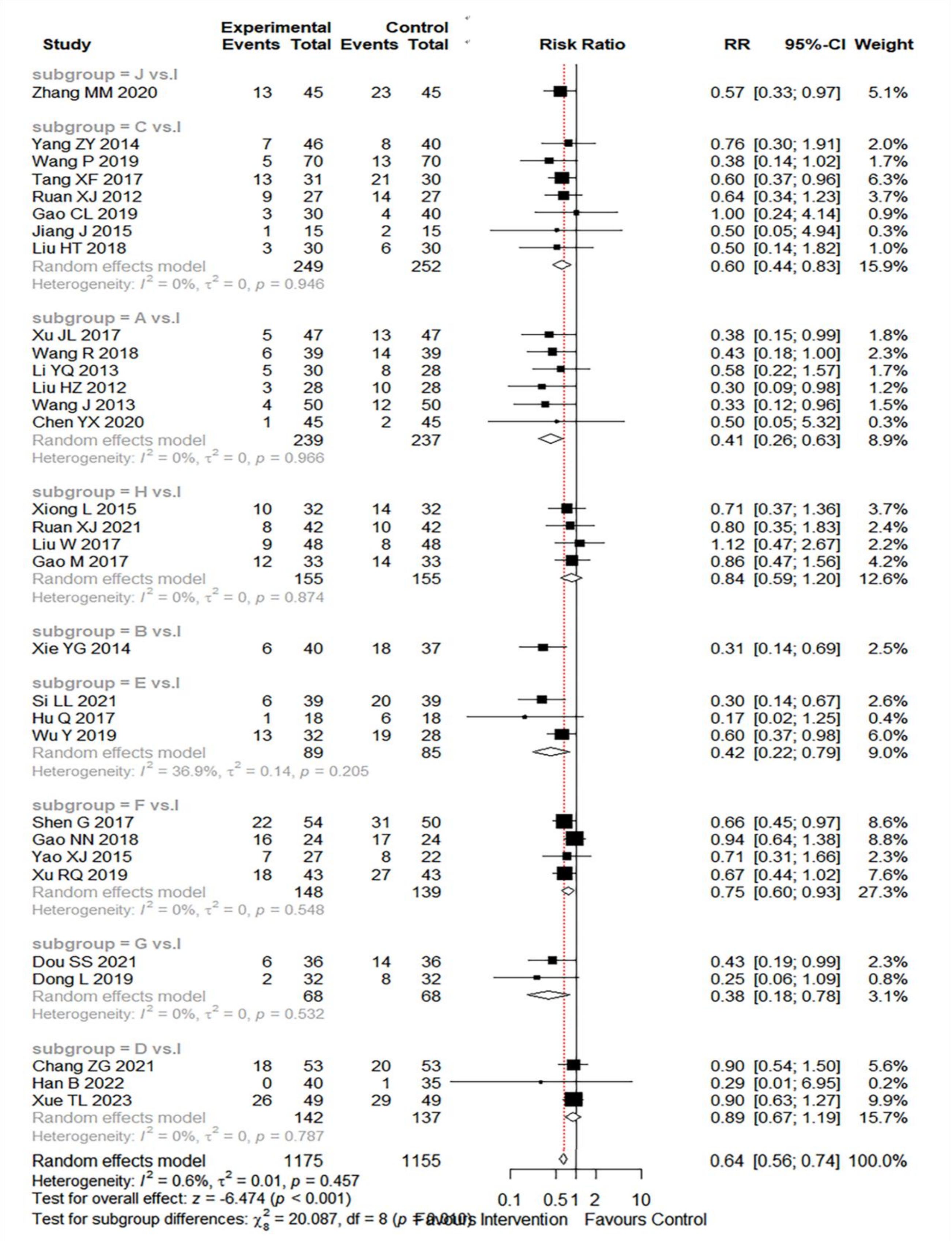

(7)

Figure S4, The forest plot Pairwise Meta-Analysis of outcomes.

(1) , Clinical effectiveness. (2), The improvement rate of KPS score. (3), Incidence of leukopenia rate. (4), Incidence of thrombocytopenia. (5),Incidence of nausea and vomiting. (6), Incidence of liver function damage. (7), Incidence of peripheral neurotoxicity.“A”, ADI+SOX. “B”, SFI+SOX. “C”, SQFZI+SOX. “E”, KAI+SOX. “F”, KLTI+SOX. “G”, HCSI+SOX. “H”, XAPI+SOX. “I”, SOX.“J”, HQI+SOX.ADI, AiDi injections. SFI, Shenfu injections. SQFZI, Shenqifuzheng injections. KAI, Kangai injections. KLTI, Kanglaitei injections. HCSI, Huachansu injections. XAPI,

Xiaoaiping injections. SOX, SOX chemotherapy regimens, HQI, Huangqi injections.

Supplement S5

Subgroup analysis and regression analysis were conducted for the seven studies involving ADI in the incidence of nausea and vomiting outcome.

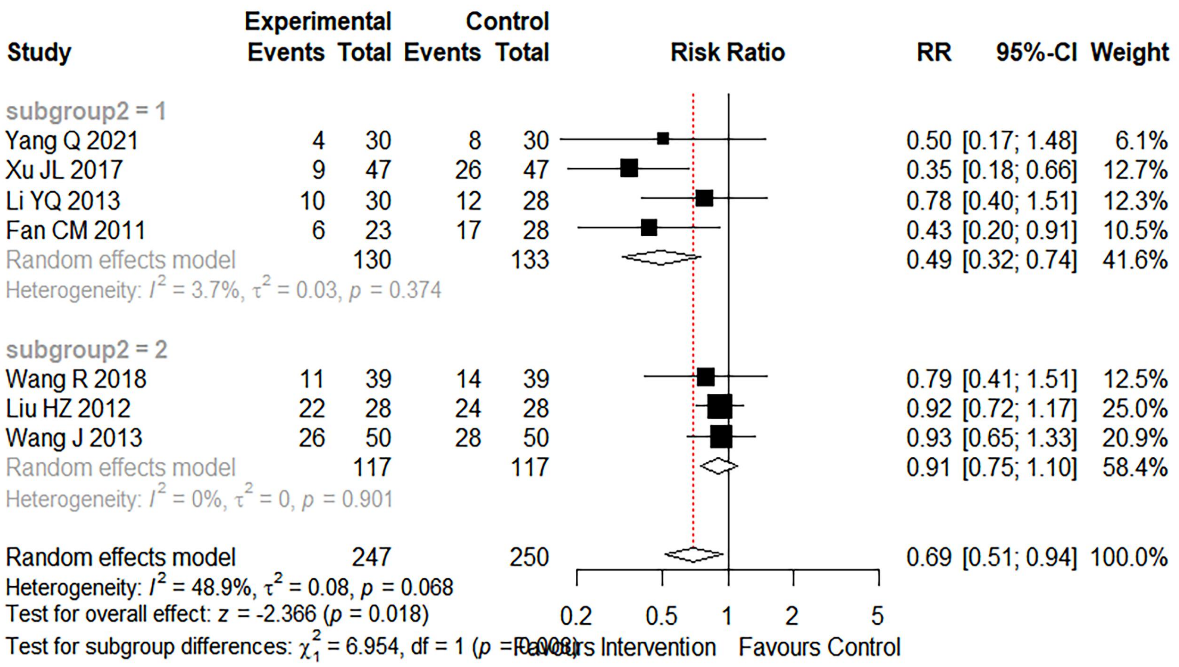

(1)

```
> metareg(IKDCS2,Antiemetic)

Mixed-Effects Model (k = 7; tau^2 estimator: REML)

tau^2 (estimated amount of residual heterogeneity):     0 (SE = 0.0318)
tau (square root of estimated tau^2 value):             0
I^2 (residual heterogeneity / unaccounted variability): 0.00%
H^2 (unaccounted variability / sampling variability):   1.00
R^2 (amount of heterogeneity accounted for):            100.00%

Test for Residual Heterogeneity:
QE(df = 5) = 3.3245, p-val = 0.6501

Test of Moderators (coefficient 2):
QM(df = 1) = 8.4240, p-val = 0.0037

Model Results:

            estimate      se     zval    pval    ci.lb    ci.ub
intrcpt      -1.3324  0.3896  -3.4200  0.0006  -2.0960  -0.5688  ***
Antiemetic    0.6177  0.2128   2.9024  0.0037   0.2006   1.0349   **

---
Signif. codes:  0 ‘***’ 0.001 ‘**’ 0.01 ‘*’ 0.05 ‘.’ 0.1 ‘ ’ 1
```

(2)

Figure S5, Subgroup analysis and regression analysis were conducted for the seven studies involving ADI in the incidence of nausea and vomiting outcome. **(1)** Subgroup analysis (2) regression analysis. The subgroup=1 indicated the use of antiemetic drugs during treatment, while the subgroup=2 indicated no use of antiemetic drugs.

Supplement S6

Sensitivity analysis.

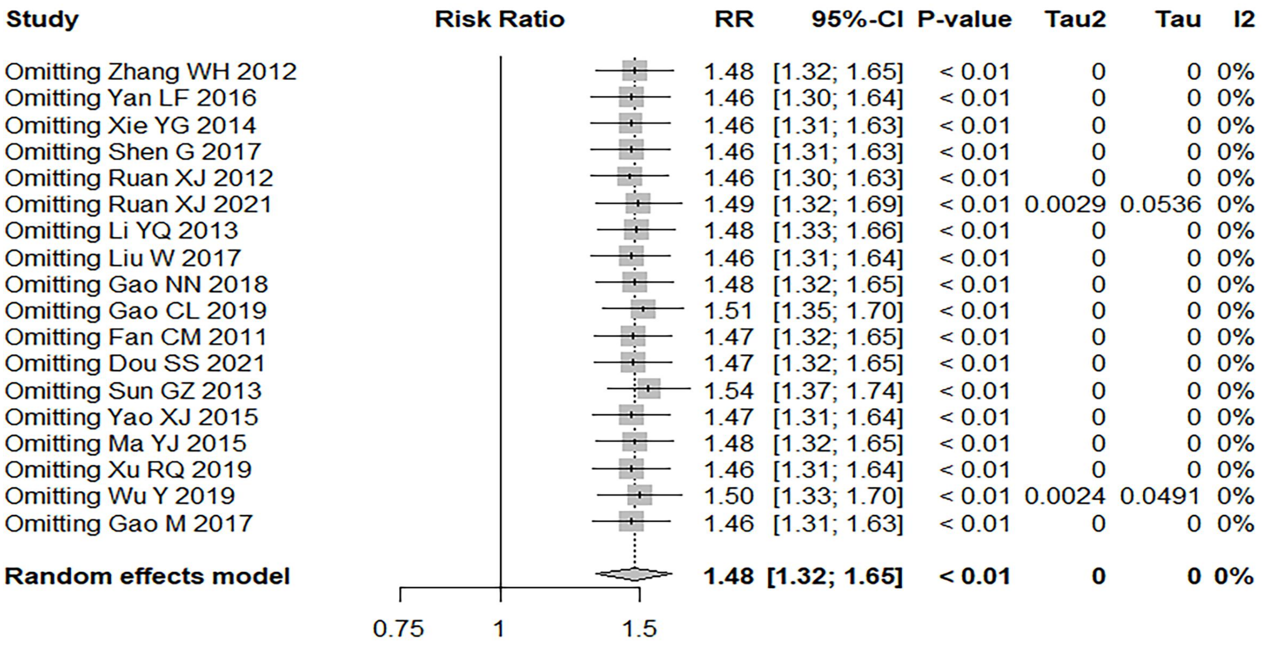

（1）

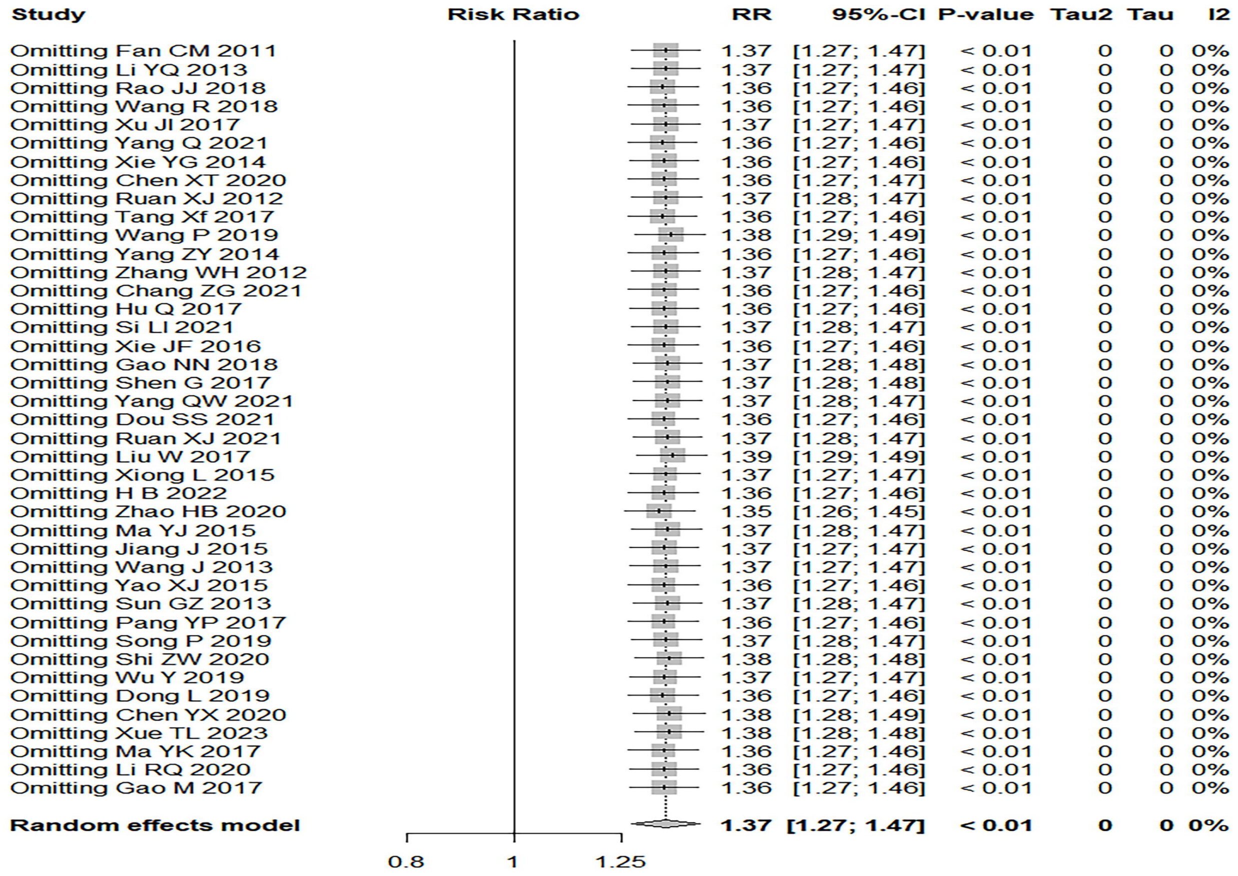
Study
Risk Ratio
RR 95%-CI P-value Tau2 Tau I2
Omitting Fan CM 2011 1.37 [1.27; 1.47] < 0.01 0 0 0%
Omitting Li YQ 2013 1.37 [1.27; 1.47] < 0.01 0 0 0%
Omitting Rao JJ 2018 1.36 [1.27; 1.46] < 0.01 0 0 0%
Omitting Wang R 2018 1.36 [1.27; 1.46] < 0.01 0 0 0%
Omitting Xu JI 2017 1.37 [1.27; 1.47] < 0.01 0 0 0%
Omitting Yang Q 2021 1.36 [1.27; 1.46] < 0.01 0 0 0%
Omitting Xie YG 2014 1.36 [1.27; 1.46] < 0.01 0 0 0%
Omitting Chen XT 2020 1.36 [1.27; 1.47] < 0.01 0 0 0%
Omitting Ruan XJ 2012 1.37 [1.28; 1.47] < 0.01 0 0 0%
Omitting Tang Xf 2017 1.36 [1.27; 1.46] < 0.01 0 0 0%
Omitting Wang P 2019 1.38 [1.29; 1.49] < 0.01 0 0 0%
Omitting Yang ZY 2014 1.36 [1.27; 1.46] < 0.01 0 0 0%
Omitting Zhang WH 2012 1.37 [1.28; 1.47] < 0.01 0 0 0%
Omitting Chang ZG 2021 1.36 [1.27; 1.46] < 0.01 0 0 0%
Omitting Hu Q 2017 1.36 [1.27; 1.46] < 0.01 0 0 0%
Omitting Si LI 2021 1.37 [1.28; 1.47] < 0.01 0 0 0%
Omitting Xie JF 2016 1.36 [1.27; 1.46] < 0.01 0 0 0%
Omitting Gao NN 2018 1.37 [1.28; 1.48] < 0.01 0 0 0%
Omitting Shen G 2017 1.37 [1.28; 1.48] < 0.01 0 0 0%
Omitting Yang QW 2021 1.37 [1.28; 1.47] < 0.01 0 0 0%
Omitting Dou SS 2021 1.36 [1.27; 1.46] < 0.01 0 0 0%
Omitting Ruan XJ 2021 1.37 [1.28; 1.47] < 0.01 0 0 0%
Omitting Liu W 2017 1.39 [1.29; 1.49] < 0.01 0 0 0%
Omitting Xiong L 2015 1.37 [1.27; 1.47] < 0.01 0 0 0%
Omitting H B 2022 1.36 [1.27; 1.46] < 0.01 0 0 0%
Omitting Zhao HB 2020 1.35 [1.26; 1.45] < 0.01 0 0 0%
Omitting Ma YJ 2015 1.37 [1.28; 1.47] < 0.01 0 0 0%
Omitting Jiang J 2015 1.37 [1.27; 1.47] < 0.01 0 0 0%
Omitting Wang J 2013 1.37 [1.27; 1.47] < 0.01 0 0 0%
Omitting Yao XJ 2015 1.36 [1.27; 1.46] < 0.01 0 0 0%
Omitting Sun GZ 2013 1.37 [1.28; 1.47] < 0.01 0 0 0%
Omitting Pang YP 2017 1.36 [1.27; 1.46] < 0.01 0 0 0%
Omitting Song P 2019 1.37 [1.28; 1.47] < 0.01 0 0 0%
Omitting Shi ZW 2020 1.38 [1.28; 1.48] < 0.01 0 0 0%
Omitting Wu Y 2019 1.37 [1.27; 1.47] < 0.01 0 0 0%
Omitting Dong L 2019 1.36 [1.27; 1.46] < 0.01 0 0 0%
Omitting Chen YX 2020 1.38 [1.28; 1.49] < 0.01 0 0 0%
Omitting Xue TL 2023 1.38 [1.28; 1.48] < 0.01 0 0 0%
Omitting Ma YK 2017 1.36 [1.27; 1.46] < 0.01 0 0 0%
Omitting Li RQ 2020 1.36 [1.27; 1.46] < 0.01 0 0 0%
Omitting Gao M 2017 1.36 [1.27; 1.46] < 0.01 0 0 0%
Random effects model 1.37 [1.27; 1.47] < 0.01 0 0 0%
0.8
1
1.25

(2)

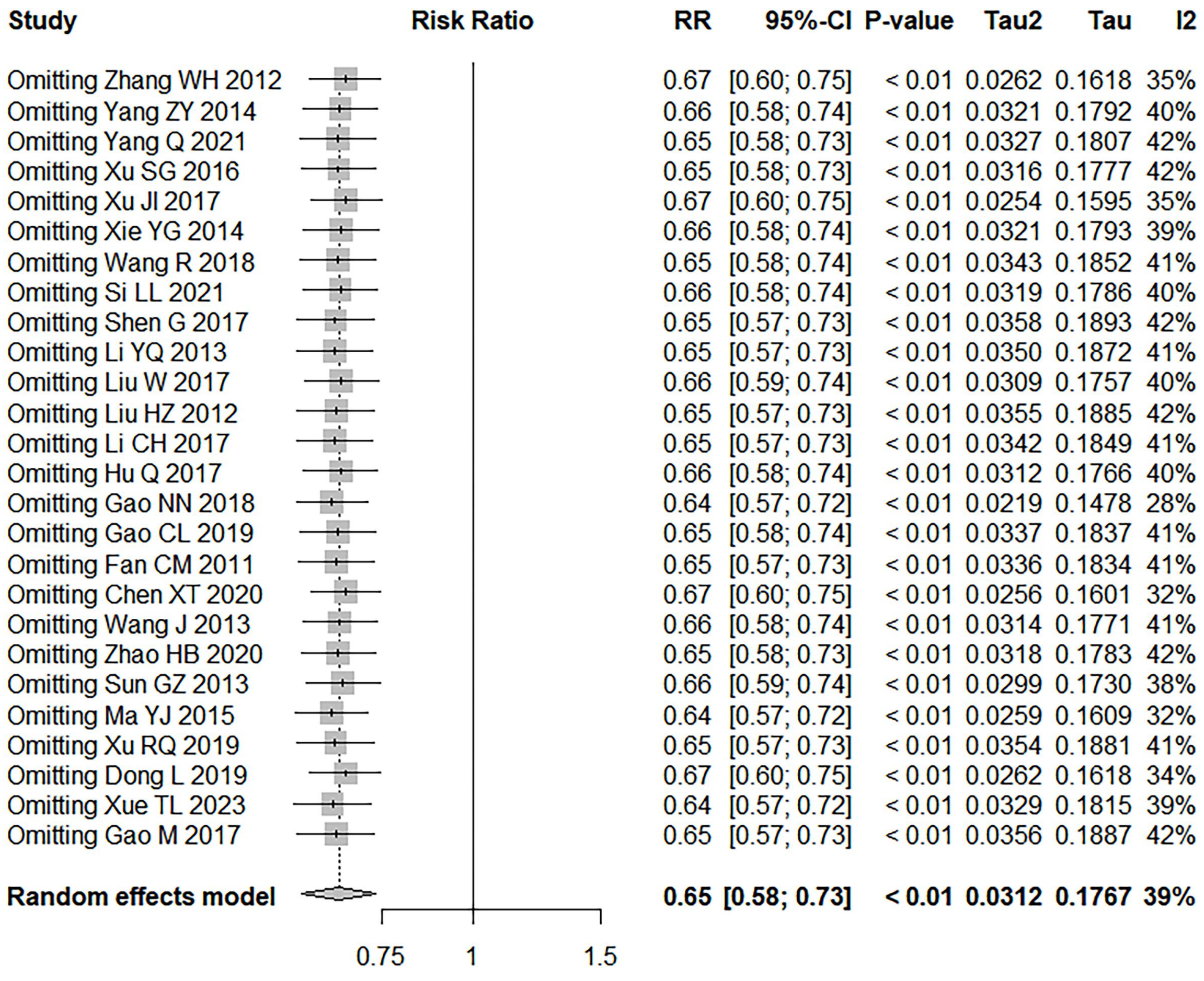
Study
Risk Ratio
RR 95%-CI P-value Tau2 Tau I2
Omitting Zhang WH 2012 0.67 [0.60; 0.75] < 0.01 0.0262 0.1618 35%
Omitting Yang ZY 2014 0.66 [0.58; 0.74] < 0.01 0.0321 0.1792 40%
Omitting Yang Q 2021 0.65 [0.58; 0.73] < 0.01 0.0327 0.1807 42%
Omitting Xu SG 2016 0.65 [0.58; 0.73] < 0.01 0.0316 0.1777 42%
Omitting Xu JI 2017 0.67 [0.60; 0.75] < 0.01 0.0254 0.1595 35%
Omitting Xie YG 2014 0.66 [0.58; 0.74] < 0.01 0.0321 0.1793 39%
Omitting Wang R 2018 0.65 [0.58; 0.74] < 0.01 0.0343 0.1852 41%
Omitting Si LL 2021 0.66 [0.58; 0.74] < 0.01 0.0319 0.1786 40%
Omitting Shen G 2017 0.65 [0.57; 0.73] < 0.01 0.0358 0.1893 42%
Omitting Li YQ 2013 0.65 [0.57; 0.73] < 0.01 0.0350 0.1872 41%
Omitting Liu W 2017 0.66 [0.59; 0.74] < 0.01 0.0309 0.1757 40%
Omitting Liu HZ 2012 0.65 [0.57; 0.73] < 0.01 0.0355 0.1885 42%
Omitting Li CH 2017 0.65 [0.57; 0.73] < 0.01 0.0342 0.1849 41%
Omitting Hu Q 2017 0.66 [0.58; 0.74] < 0.01 0.0312 0.1766 40%
Omitting Gao NN 2018 0.64 [0.57; 0.72] < 0.01 0.0219 0.1478 28%
Omitting Gao CL 2019 0.65 [0.58; 0.74] < 0.01 0.0337 0.1837 41%
Omitting Fan CM 2011 0.65 [0.57; 0.73] < 0.01 0.0336 0.1834 41%
Omitting Chen XT 2020 0.67 [0.60; 0.75] < 0.01 0.0256 0.1601 32%
Omitting Wang J 2013 0.66 [0.58; 0.74] < 0.01 0.0314 0.1771 41%
Omitting Zhao HB 2020 0.65 [0.58; 0.73] < 0.01 0.0318 0.1783 42%
Omitting Sun GZ 2013 0.66 [0.59; 0.74] < 0.01 0.0299 0.1730 38%
Omitting Ma YJ 2015 0.64 [0.57; 0.72] < 0.01 0.0259 0.1609 32%
Omitting Xu RQ 2019 0.65 [0.57; 0.73] < 0.01 0.0354 0.1881 41%
Omitting Dong L 2019 0.67 [0.60; 0.75] < 0.01 0.0262 0.1618 34%
Omitting Xue TL 2023 0.64 [0.57; 0.72] < 0.01 0.0329 0.1815 39%
Omitting Gao M 2017 0.65 [0.57; 0.73] < 0.01 0.0356 0.1887 42%
Random effects model 0.65 [0.58; 0.73] < 0.01 0.0312 0.1767 39%
0.75
1
1.5

(3)

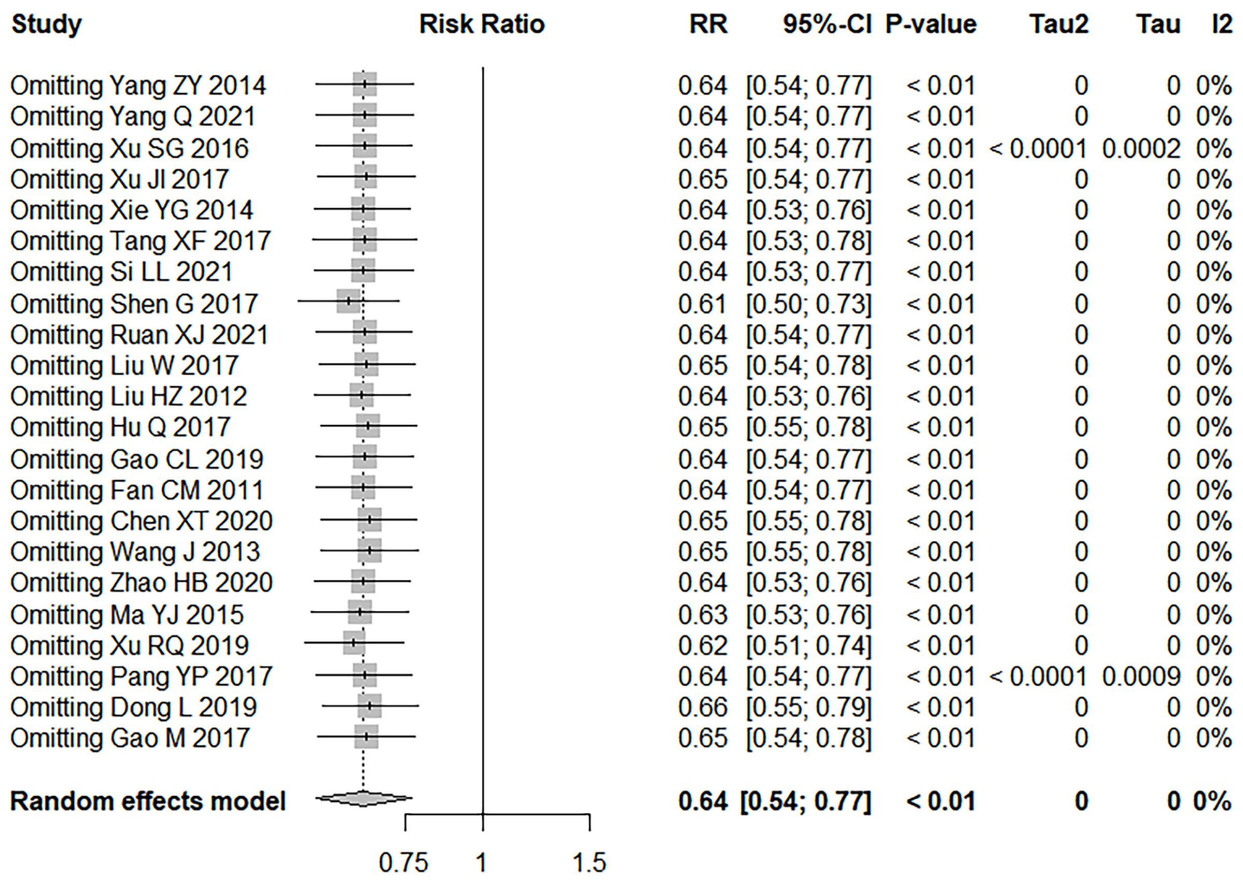
Study
Risk Ratio
RR
95%-CI
P-value
Tau2
Tau
I2
Omitting Yang ZY 2014 0.64 [0.54; 0.77] < 0.01 0 0 0%
Omitting Yang Q 2021 0.64 [0.54; 0.77] < 0.01 0 0 0%
Omitting Xu SG 2016 0.64 [0.54; 0.77] < 0.01 < 0.0001 0.0002 0%
Omitting Xu JI 2017 0.65 [0.54; 0.77] < 0.01 0 0 0%
Omitting Xie YG 2014 0.64 [0.53; 0.76] < 0.01 0 0 0%
Omitting Tang XF 2017 0.64 [0.53; 0.78] < 0.01 0 0 0%
Omitting Si LL 2021 0.64 [0.53; 0.77] < 0.01 0 0 0%
Omitting Shen G 2017 0.61 [0.50; 0.73] < 0.01 0 0 0%
Omitting Ruan XJ 2021 0.64 [0.54; 0.77] < 0.01 0 0 0%
Omitting Liu W 2017 0.65 [0.54; 0.78] < 0.01 0 0 0%
Omitting Liu HZ 2012 0.64 [0.53; 0.76] < 0.01 0 0 0%
Omitting Hu Q 2017 0.65 [0.55; 0.78] < 0.01 0 0 0%
Omitting Gao CL 2019 0.64 [0.54; 0.77] < 0.01 0 0 0%
Omitting Fan CM 2011 0.64 [0.54; 0.77] < 0.01 0 0 0%
Omitting Chen XT 2020 0.65 [0.55; 0.78] < 0.01 0 0 0%
Omitting Wang J 2013 0.65 [0.55; 0.78] < 0.01 0 0 0%
Omitting Zhao HB 2020 0.64 [0.53; 0.76] < 0.01 0 0 0%
Omitting Ma YJ 2015 0.63 [0.53; 0.76] < 0.01 0 0 0%
Omitting Xu RQ 2019 0.62 [0.51; 0.74] < 0.01 0 0 0%
Omitting Pang YP 2017 0.64 [0.54; 0.77] < 0.01 < 0.0001 0.0009 0%
Omitting Dong L 2019 0.66 [0.55; 0.79] < 0.01 0 0 0%
Omitting Gao M 2017 0.65 [0.54; 0.78] < 0.01 0 0 0%
Random effects model 0.64 [0.54; 0.77] < 0.01 0 0 0%
0.75
1
1.5

（4）

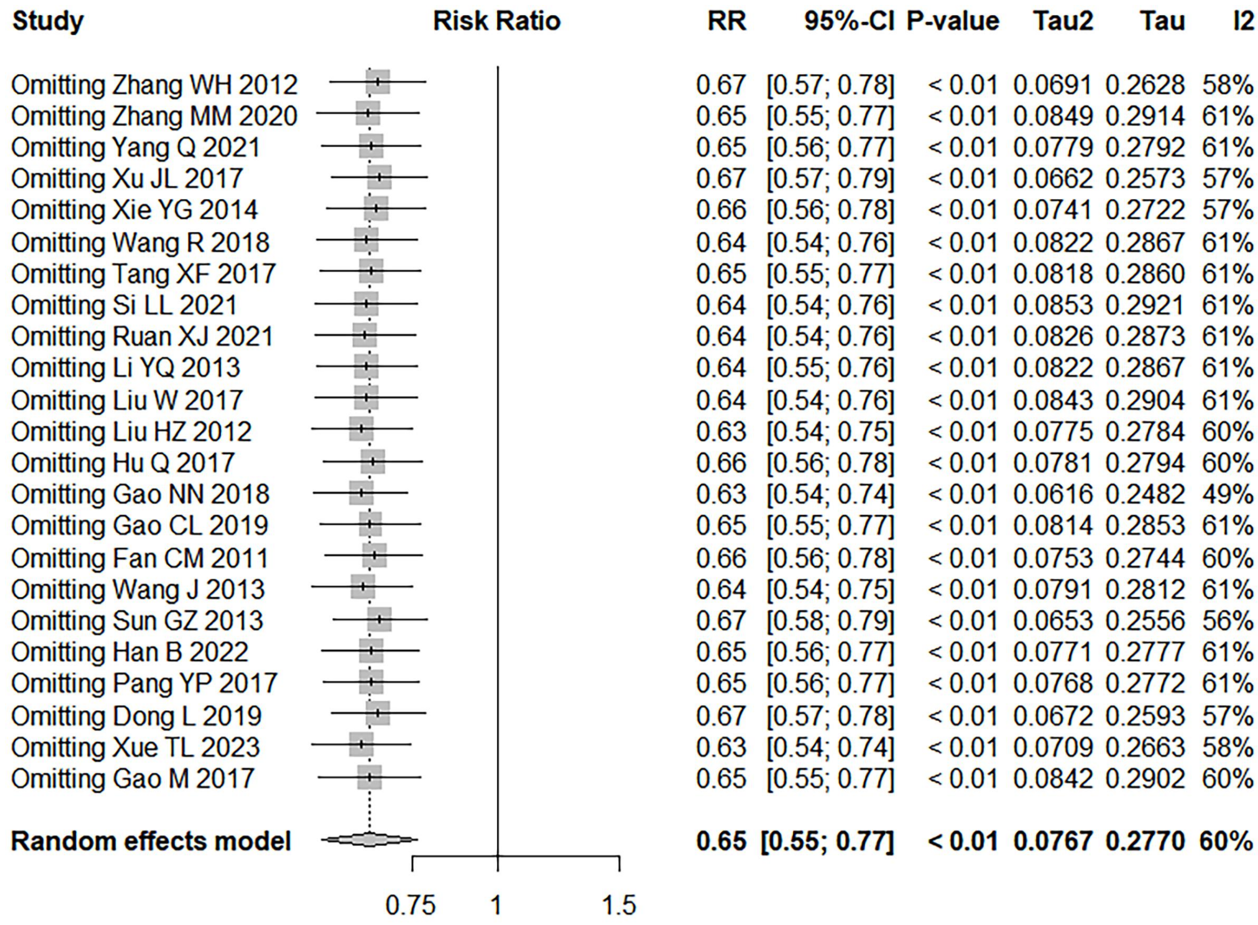
Study
Risk Ratio
RR
95%-CI
P-value
Tau2
Tau
I2
Omitting Zhang WH 2012 0.67 [0.57; 0.78] < 0.01 0.0691 0.2628 58%
Omitting Zhang MM 2020 0.65 [0.55; 0.77] < 0.01 0.0849 0.2914 61%
Omitting Yang Q 2021 0.65 [0.56; 0.77] < 0.01 0.0779 0.2792 61%
Omitting Xu JL 2017 0.67 [0.57; 0.79] < 0.01 0.0662 0.2573 57%
Omitting Xie YG 2014 0.66 [0.56; 0.78] < 0.01 0.0741 0.2722 57%
Omitting Wang R 2018 0.64 [0.54; 0.76] < 0.01 0.0822 0.2867 61%
Omitting Tang XF 2017 0.65 [0.55; 0.77] < 0.01 0.0818 0.2860 61%
Omitting Si LL 2021 0.64 [0.54; 0.76] < 0.01 0.0853 0.2921 61%
Omitting Ruan XJ 2021 0.64 [0.54; 0.76] < 0.01 0.0826 0.2873 61%
Omitting Li YQ 2013 0.64 [0.55; 0.76] < 0.01 0.0822 0.2867 61%
Omitting Liu W 2017 0.64 [0.54; 0.76] < 0.01 0.0843 0.2904 61%
Omitting Liu HZ 2012 0.63 [0.54; 0.75] < 0.01 0.0775 0.2784 60%
Omitting Hu Q 2017 0.66 [0.56; 0.78] < 0.01 0.0781 0.2794 60%
Omitting Gao NN 2018 0.63 [0.54; 0.74] < 0.01 0.0616 0.2482 49%
Omitting Gao CL 2019 0.65 [0.55; 0.77] < 0.01 0.0814 0.2853 61%
Omitting Fan CM 2011 0.66 [0.56; 0.78] < 0.01 0.0753 0.2744 60%
Omitting Wang J 2013 0.64 [0.54; 0.75] < 0.01 0.0791 0.2812 61%
Omitting Sun GZ 2013 0.67 [0.58; 0.79] < 0.01 0.0653 0.2556 56%
Omitting Han B 2022 0.65 [0.56; 0.77] < 0.01 0.0771 0.2777 61%
Omitting Pang YP 2017 0.65 [0.56; 0.77] < 0.01 0.0768 0.2772 61%
Omitting Dong L 2019 0.67 [0.57; 0.78] < 0.01 0.0672 0.2593 57%
Omitting Xue TL 2023 0.63 [0.54; 0.74] < 0.01 0.0709 0.2663 58%
Omitting Gao M 2017 0.65 [0.55; 0.77] < 0.01 0.0842 0.2902 60%
Random effects model 0.65 [0.55; 0.77] < 0.01 0.0767 0.2770 60%
0.75
1
1.5

(5)

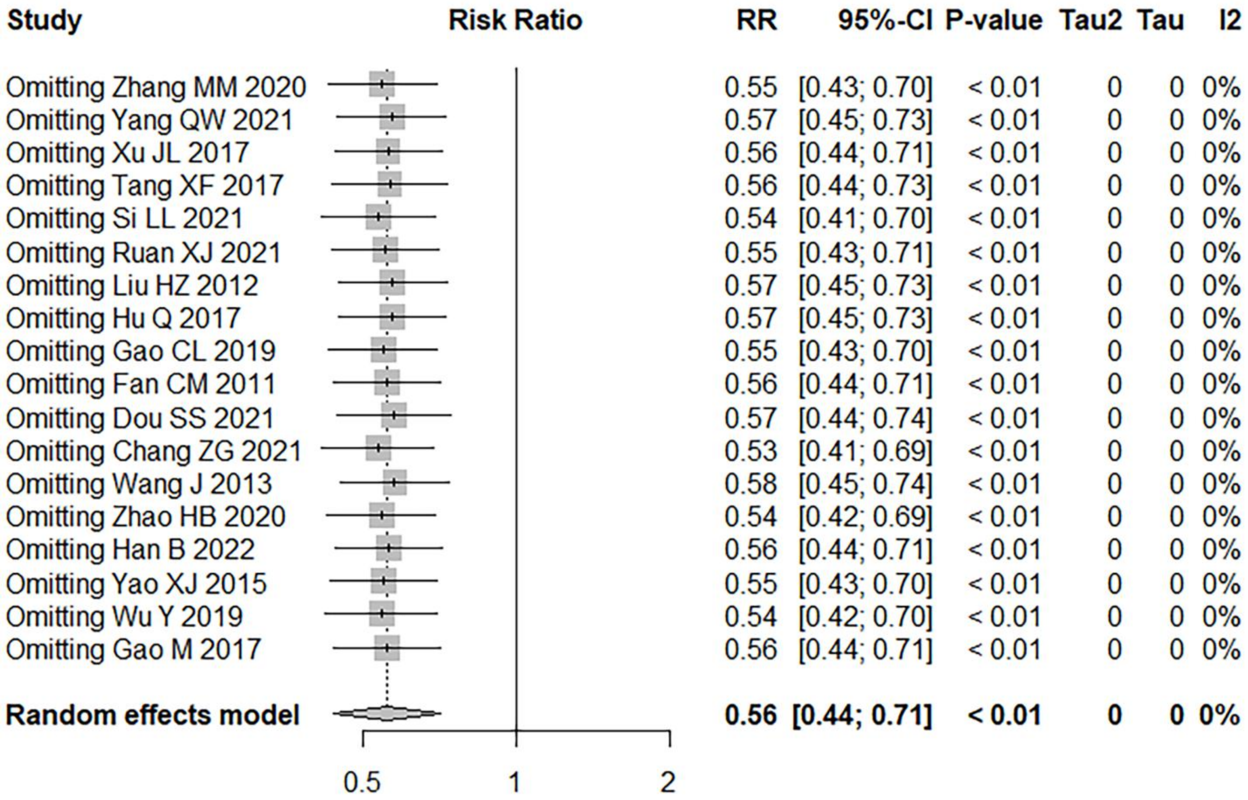
Study
Risk Ratio
RR 95%-CI P-value Tau2 Tau I2
Omitting Zhang MM 2020 0.55 [0.43; 0.70] < 0.01 0 0 0%
Omitting Yang QW 2021 0.57 [0.45; 0.73] < 0.01 0 0 0%
Omitting Xu JL 2017 0.56 [0.44; 0.71] < 0.01 0 0 0%
Omitting Tang XF 2017 0.56 [0.44; 0.73] < 0.01 0 0 0%
Omitting Si LL 2021 0.54 [0.41; 0.70] < 0.01 0 0 0%
Omitting Ruan XJ 2021 0.55 [0.43; 0.71] < 0.01 0 0 0%
Omitting Liu HZ 2012 0.57 [0.45; 0.73] < 0.01 0 0 0%
Omitting Hu Q 2017 0.57 [0.45; 0.73] < 0.01 0 0 0%
Omitting Gao CL 2019 0.55 [0.43; 0.70] < 0.01 0 0 0%
Omitting Fan CM 2011 0.56 [0.44; 0.71] < 0.01 0 0 0%
Omitting Dou SS 2021 0.57 [0.44; 0.74] < 0.01 0 0 0%
Omitting Chang ZG 2021 0.53 [0.41; 0.69] < 0.01 0 0 0%
Omitting Wang J 2013 0.58 [0.45; 0.74] < 0.01 0 0 0%
Omitting Zhao HB 2020 0.54 [0.42; 0.69] < 0.01 0 0 0%
Omitting Han B 2022 0.56 [0.44; 0.71] < 0.01 0 0 0%
Omitting Yao XJ 2015 0.55 [0.43; 0.70] < 0.01 0 0 0%
Omitting Wu Y 2019 0.54 [0.42; 0.70] < 0.01 0 0 0%
Omitting Gao M 2017 0.56 [0.44; 0.71] < 0.01 0 0 0%
Random effects model 0.56 [0.44; 0.71] < 0.01 0 0 0%
0.5
1
2

(6)

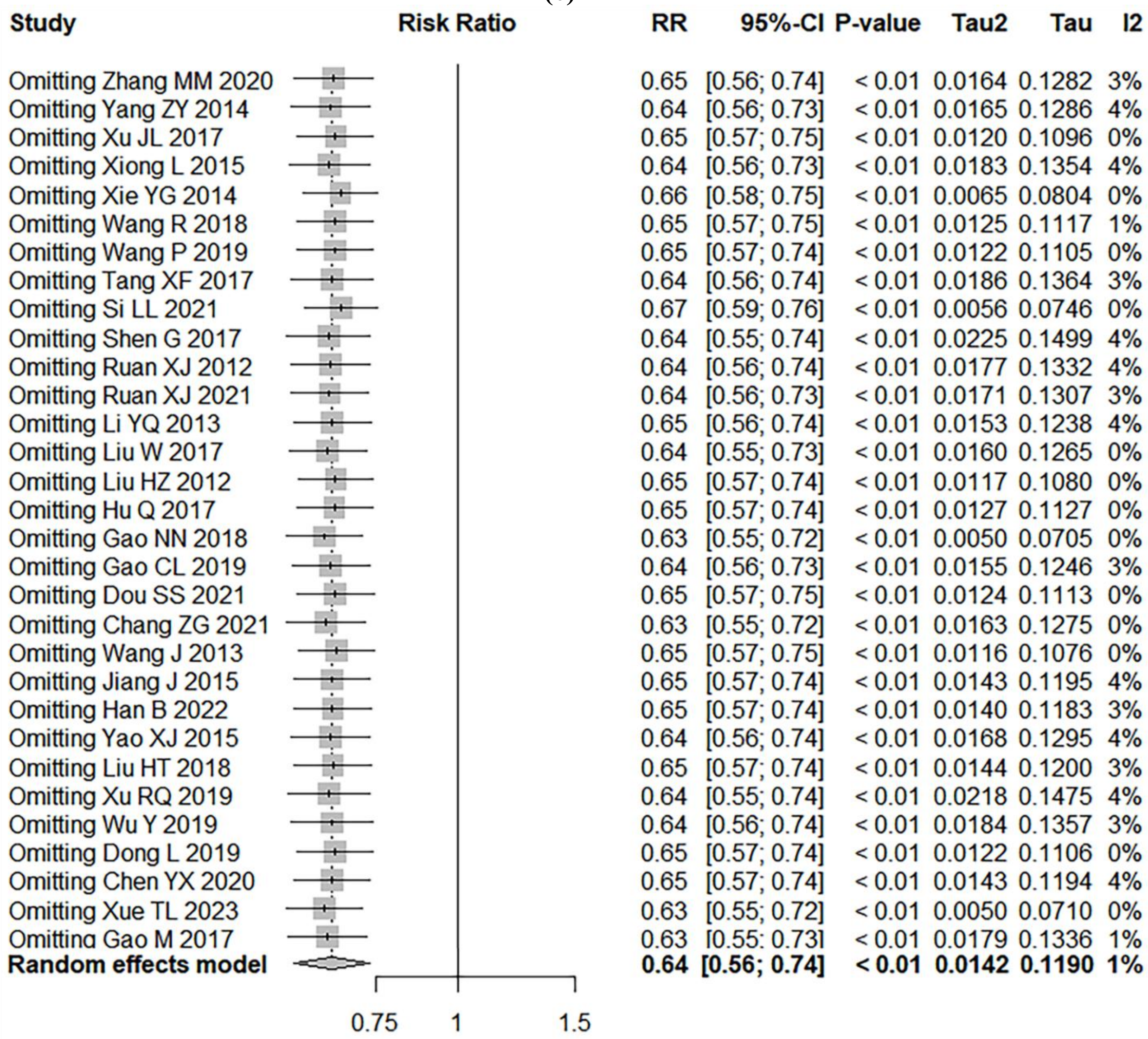
Study
Risk Ratio
RR 95%-CI P-value Tau2 Tau I2
Omitting Zhang MM 2020 0.65 [0.56; 0.74] < 0.01 0.0164 0.1282 3%
Omitting Yang ZY 2014 0.64 [0.56; 0.73] < 0.01 0.0165 0.1286 4%
Omitting Xu JL 2017 0.65 [0.57; 0.75] < 0.01 0.0120 0.1096 0%
Omitting Xiong L 2015 0.64 [0.56; 0.73] < 0.01 0.0183 0.1354 4%
Omitting Xie YG 2014 0.66 [0.58; 0.75] < 0.01 0.0065 0.0804 0%
Omitting Wang R 2018 0.65 [0.57; 0.75] < 0.01 0.0125 0.1117 1%
Omitting Wang P 2019 0.65 [0.57; 0.74] < 0.01 0.0122 0.1105 0%
Omitting Tang XF 2017 0.64 [0.56; 0.74] < 0.01 0.0186 0.1364 3%
Omitting Si LL 2021 0.67 [0.59; 0.76] < 0.01 0.0056 0.0746 0%
Omitting Shen G 2017 0.64 [0.55; 0.74] < 0.01 0.0225 0.1499 4%
Omitting Ruan XJ 2012 0.64 [0.56; 0.74] < 0.01 0.0177 0.1332 4%
Omitting Ruan XJ 2021 0.64 [0.56; 0.73] < 0.01 0.0171 0.1307 3%
Omitting Li YQ 2013 0.65 [0.56; 0.74] < 0.01 0.0153 0.1238 4%
Omitting Liu W 2017 0.64 [0.55; 0.73] < 0.01 0.0160 0.1265 0%
Omitting Liu HZ 2012 0.65 [0.57; 0.74] < 0.01 0.0117 0.1080 0%
Omitting Hu Q 2017 0.65 [0.57; 0.74] < 0.01 0.0127 0.1127 0%
Omitting Gao NN 2018 0.63 [0.55; 0.72] < 0.01 0.0050 0.0705 0%
Omitting Gao CL 2019 0.64 [0.56; 0.73] < 0.01 0.0155 0.1246 3%
Omitting Dou SS 2021 0.65 [0.57; 0.75] < 0.01 0.0124 0.1113 0%
Omitting Chang ZG 2021 0.63 [0.55; 0.72] < 0.01 0.0163 0.1275 0%
Omitting Wang J 2013 0.65 [0.57; 0.75] < 0.01 0.0116 0.1076 0%
Omitting Jiang J 2015 0.65 [0.57; 0.74] < 0.01 0.0143 0.1195 4%
Omitting Han B 2022 0.65 [0.57; 0.74] < 0.01 0.0140 0.1183 3%
Omitting Yao XJ 2015 0.64 [0.56; 0.74] < 0.01 0.0168 0.1295 4%
Omitting Liu HT 2018 0.65 [0.57; 0.74] < 0.01 0.0144 0.1200 3%
Omitting Xu RQ 2019 0.64 [0.55; 0.74] < 0.01 0.0218 0.1475 4%
Omitting Wu Y 2019 0.64 [0.56; 0.74] < 0.01 0.0184 0.1357 3%
Omitting Dong L 2019 0.65 [0.57; 0.74] < 0.01 0.0122 0.1106 0%
Omitting Chen YX 2020 0.65 [0.57; 0.74] < 0.01 0.0143 0.1194 4%
Omitting Xue TL 2023 0.63 [0.55; 0.72] < 0.01 0.0050 0.0710 0%
Omitting Gao M 2017 0.63 [0.55; 0.73] < 0.01 0.0179 0.1336 1%
Random effects model 0.64 [0.56; 0.74] < 0.01 0.0142 0.1190 1%
0.75
1
1.5

（7）

Figure S6，Sensitivity analysis of outcomes. (1), Clinical effectiveness. (2), The improvement rate of KPS score. (3), Incidence of leukopenia rate. (4), Incidence of thrombocytopenia. (5),Incidence of nausea and vomiting. (6), Incidence of liver function damage. (7), Incidence of peripheral neurotoxicity. “A”, ADI+SOX. “B”, SFI+SOX. “C”, SQFZI+SOX. “E”, KAI+SOX. “F”, KLTI+SOX. “G”, HCSI+SOX. “H”, XAPI+SOX. “I”, SOX.“J”, HQI+SOX.ADI, AiDi injections. SFI, Shenfu injections. SQFZI, Shenqifuzheng injections. KAI, Kangai injections. KLTI, Kanglaitei injections. HCSI, Huachansu injections. XAPI, Xiaoaiping injections. SOX, SOX chemotherapy regimens, HQI, Huangqi injections.

Supplement S7

The specific trajectory maps and density maps.

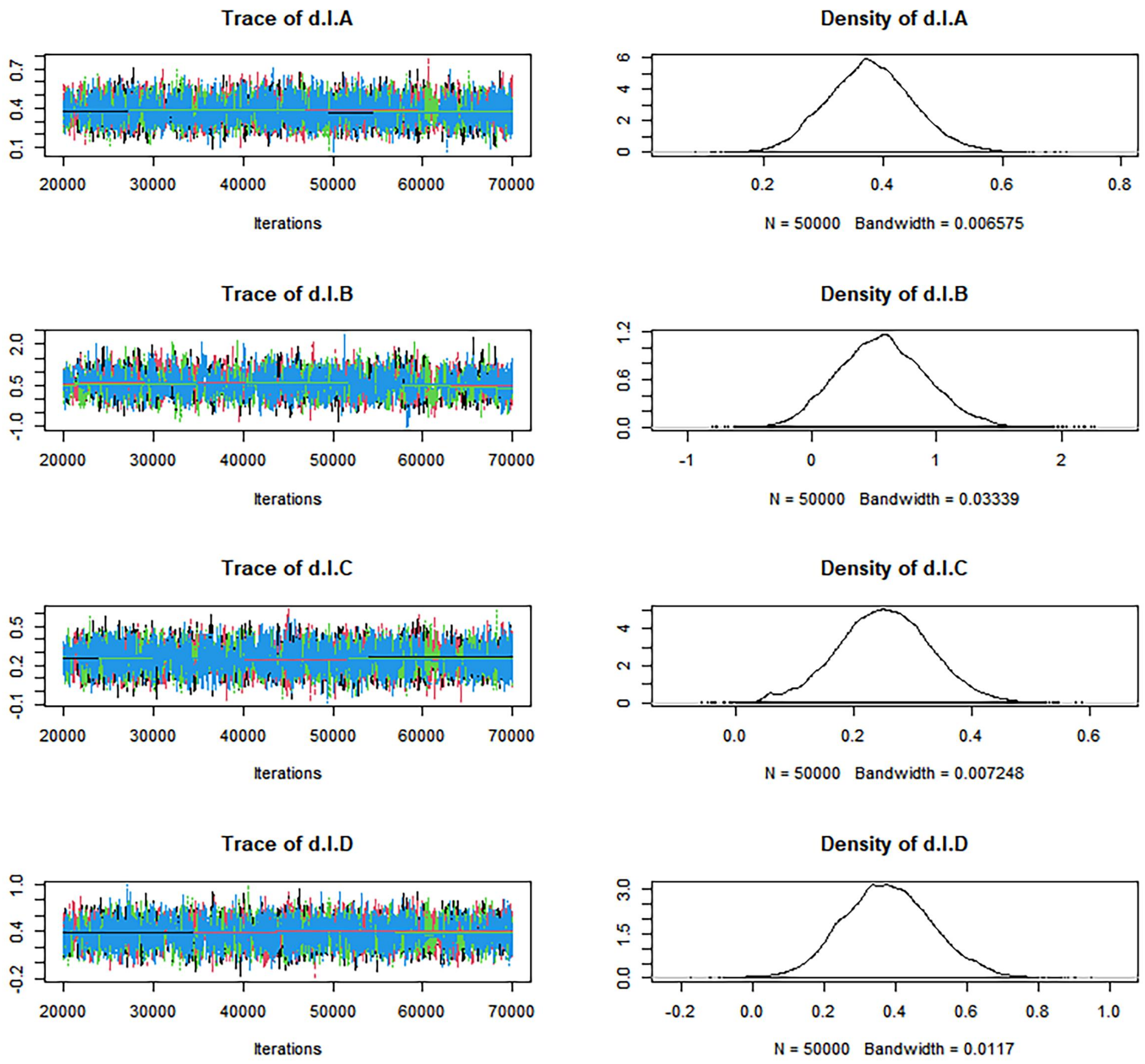

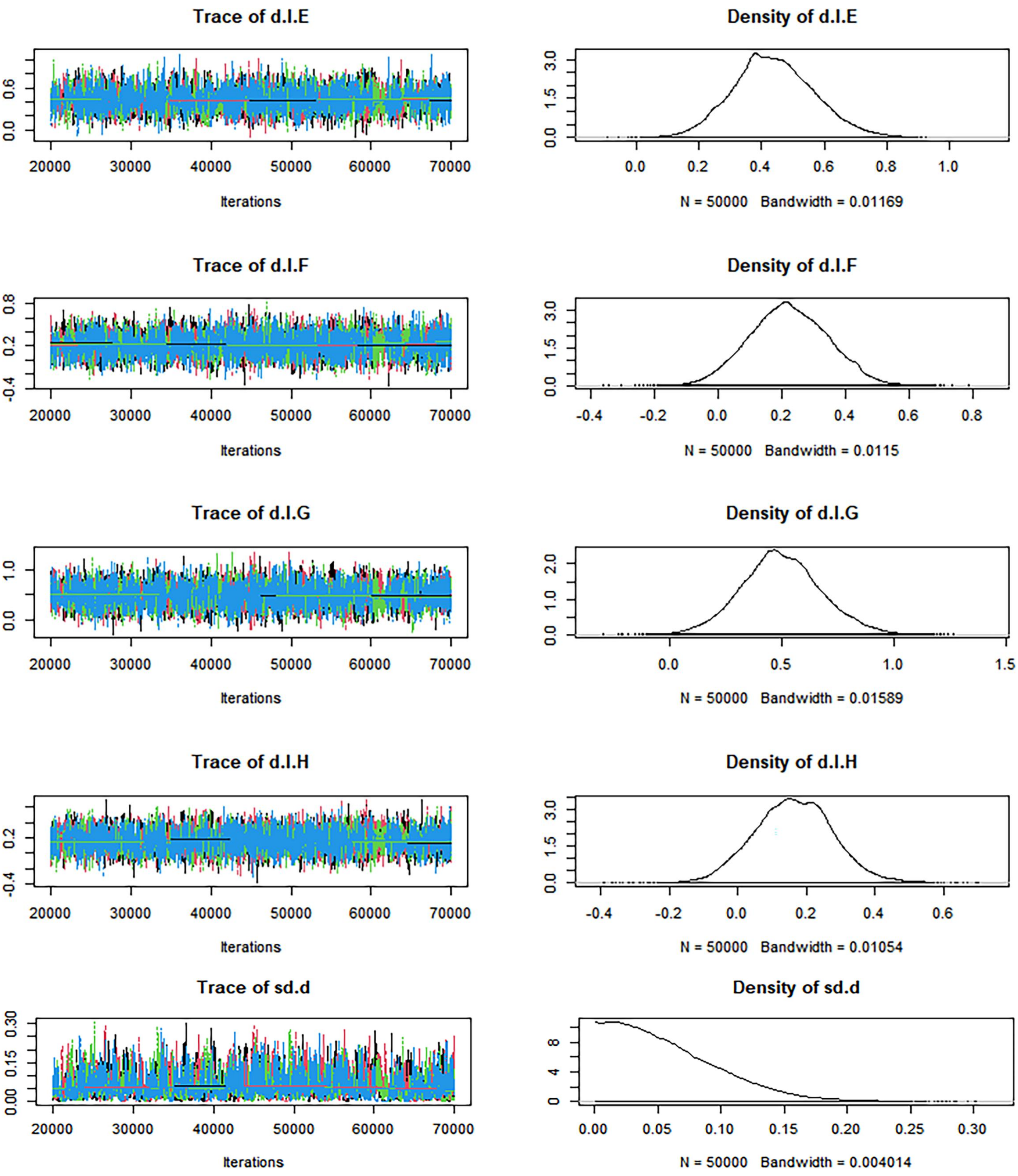
Trace of d.I.E
Density of d.I.E
Iterations
N = 50000 Bandwidth = 0.01169
Trace of d.I.F
Density of d.I.F
Iterations
N = 50000 Bandwidth = 0.0115
Trace of d.I.G
Density of d.I.G
Iterations
N = 50000 Bandwidth = 0.01589
Trace of d.I.H
Density of d.I.H
Iterations
N = 50000 Bandwidth = 0.01054
Trace of sd.d
Density of sd.d
Iterations
N = 50000 Bandwidth = 0.004014

(1)

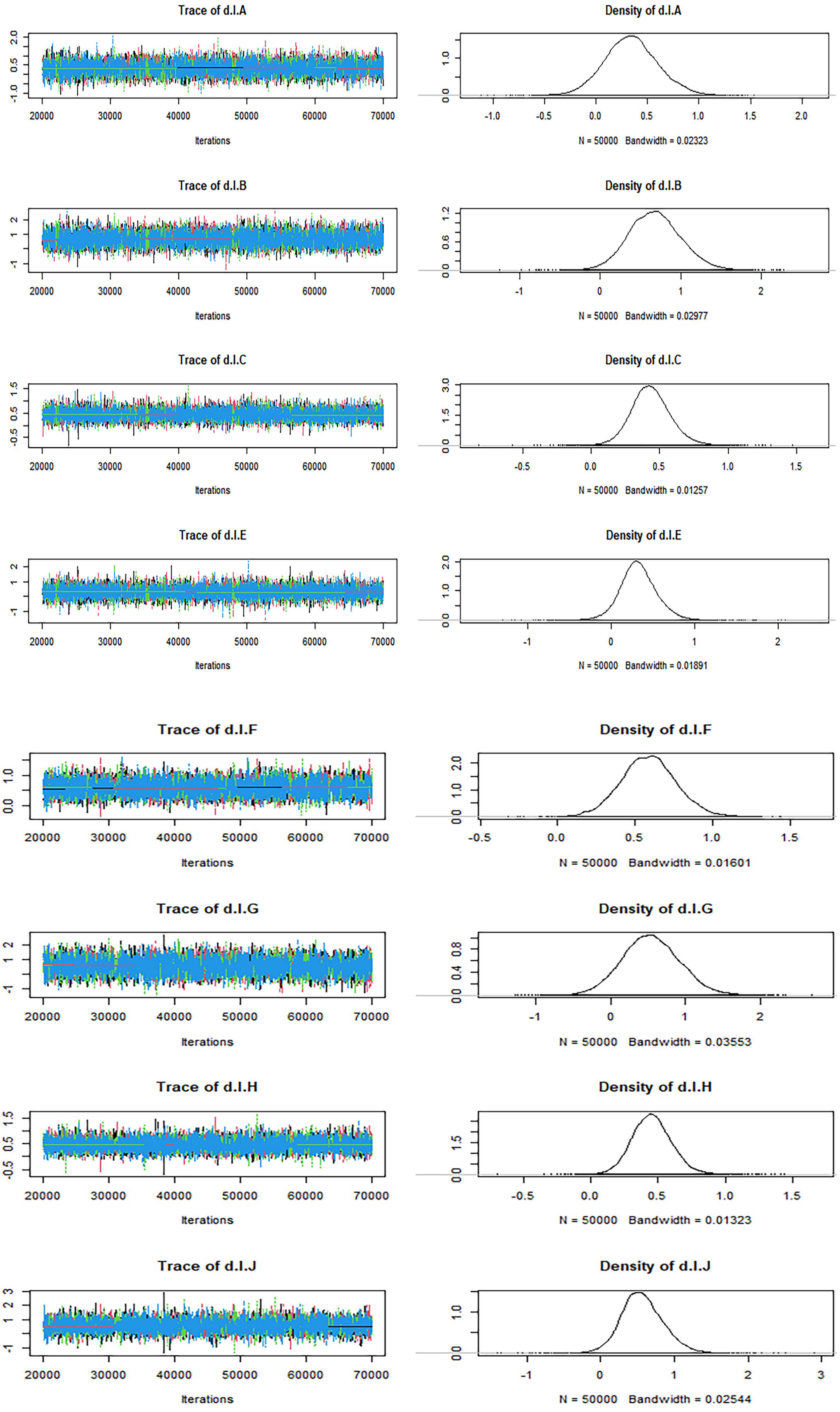

Trace of d.I.A
Iterations
Density of d.I.A
N = 50000 Bandwidth = 0.02323
Trace of d.I.B
Iterations
Density of d.I.B
N = 50000 Bandwidth = 0.02977
Trace of d.I.C
Iterations
Density of d.I.C
N = 50000 Bandwidth = 0.01257
Trace of d.I.E
Iterations
Density of d.I.E
N = 50000 Bandwidth = 0.01891
Trace of d.I.F
Iterations
Density of d.I.F
N = 50000 Bandwidth = 0.01601
Trace of d.I.G
Iterations
Density of d.I.G
N = 50000 Bandwidth = 0.03553
Trace of d.I.H
Iterations
Density of d.I.H
N = 50000 Bandwidth = 0.01323
Trace of d.I.J
Iterations
Density of d.I.J
N = 50000 Bandwidth = 0.02544

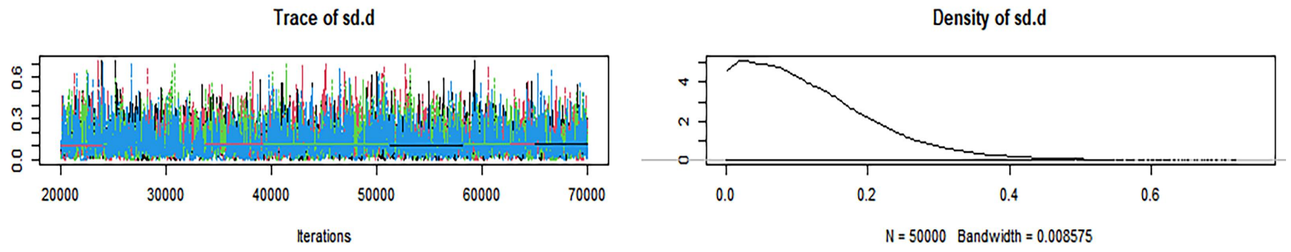
Trace of sd.d
Iterations
Density of sd.d
N = 50000 Bandwidth = 0.008575

(2)

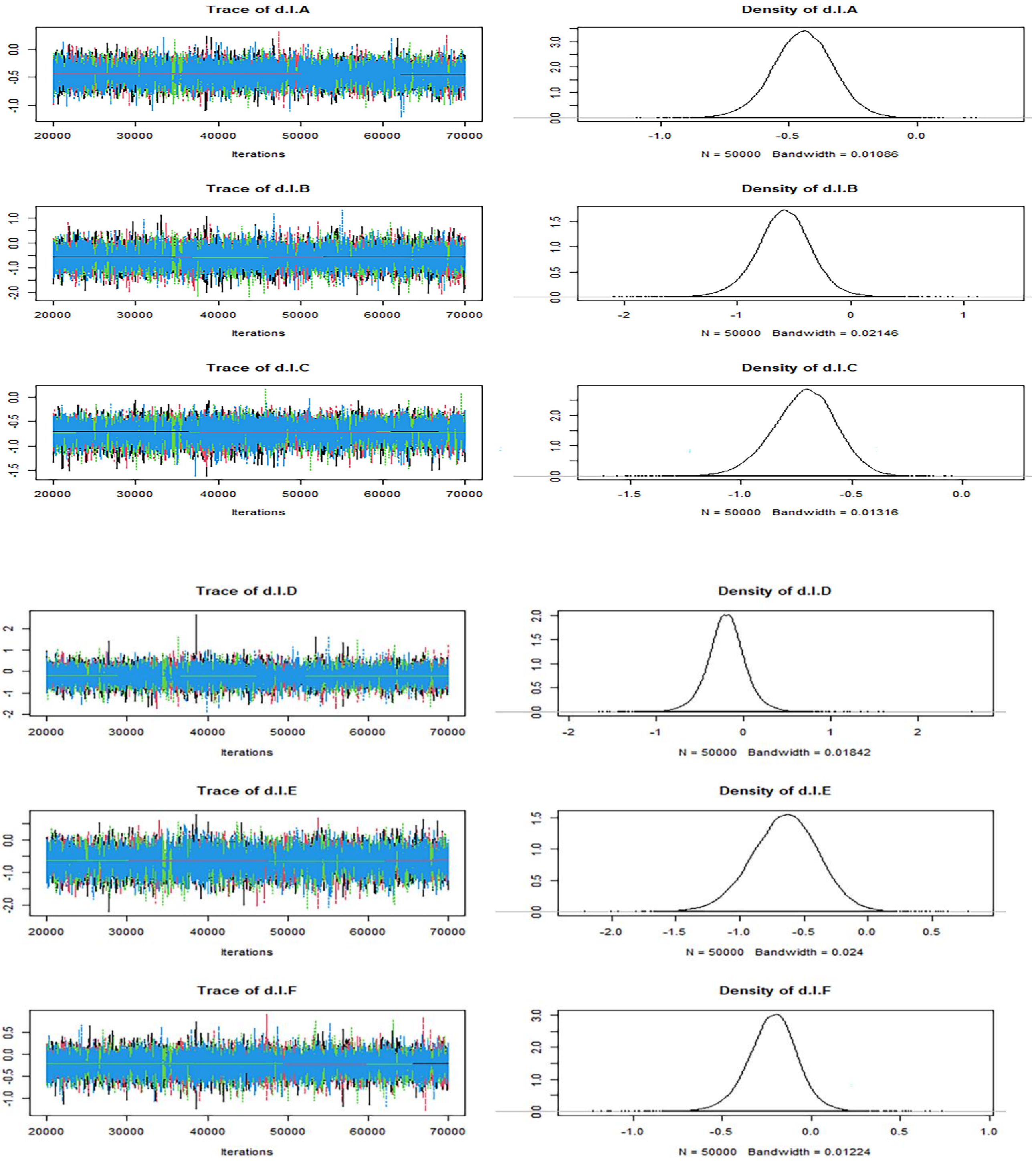
Trace of d.I.A
Iterations
Density of d.I.A
N = 50000 Bandwidth = 0.01086
Trace of d.I.B
Iterations
Density of d.I.B
N = 50000 Bandwidth = 0.02146
Trace of d.I.C
Iterations
Density of d.I.C
N = 50000 Bandwidth = 0.01316
Trace of d.I.D
Iterations
Density of d.I.D
N = 50000 Bandwidth = 0.01842
Trace of d.I.E
Iterations
Density of d.I.E
N = 50000 Bandwidth = 0.024
Trace of d.I.F
Iterations
Density of d.I.F
N = 50000 Bandwidth = 0.01224

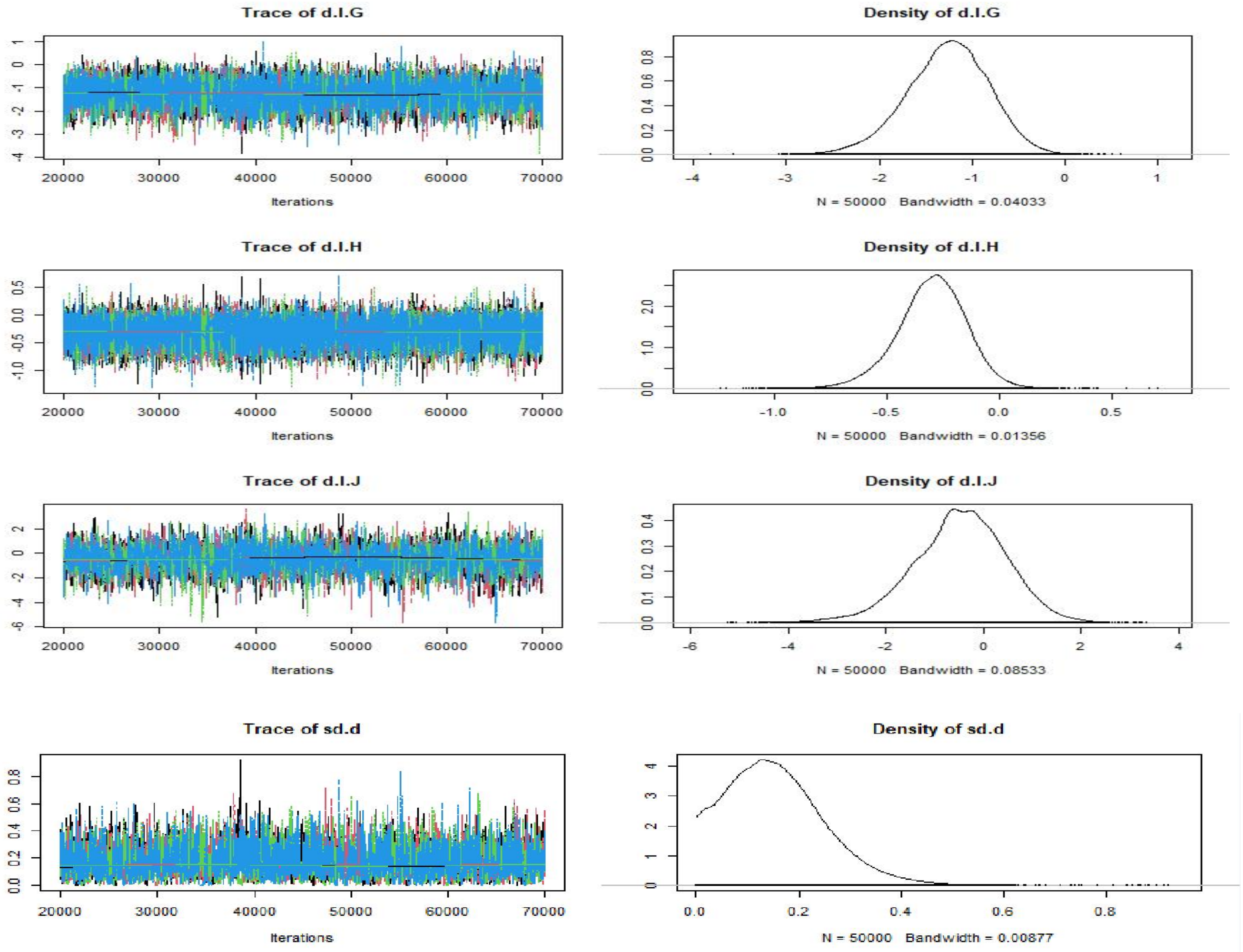
Trace of d.l.G
Iterations
Density of d.l.G
N = 50000 Bandwidth = 0.04033
Trace of d.l.H
Iterations
Density of d.l.H
N = 50000 Bandwidth = 0.01356
Trace of d.l.J
Iterations
Density of d.l.J
N = 50000 Bandwidth = 0.08533
Trace of sd.d
Iterations
Density of sd.d
N = 50000 Bandwidth = 0.00877

(3)

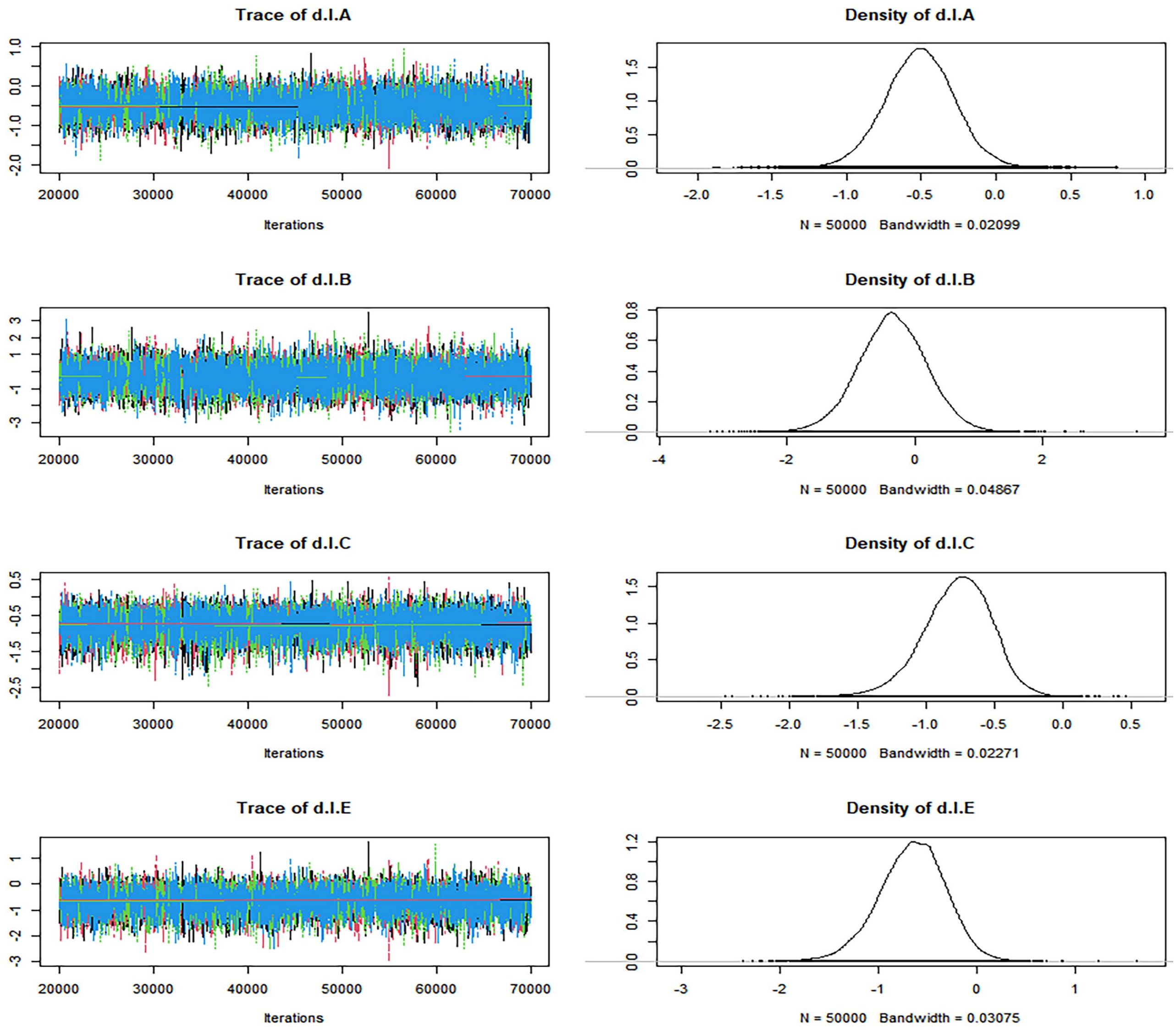
Trace of d.I.A
Iterations
Density of d.I.A
N = 50000 Bandwidth = 0.02099
Trace of d.I.B
Iterations
Density of d.I.B
N = 50000 Bandwidth = 0.04867
Trace of d.I.C
Iterations
Density of d.I.C
N = 50000 Bandwidth = 0.02271
Trace of d.I.E
Iterations
Density of d.I.E
N = 50000 Bandwidth = 0.03075

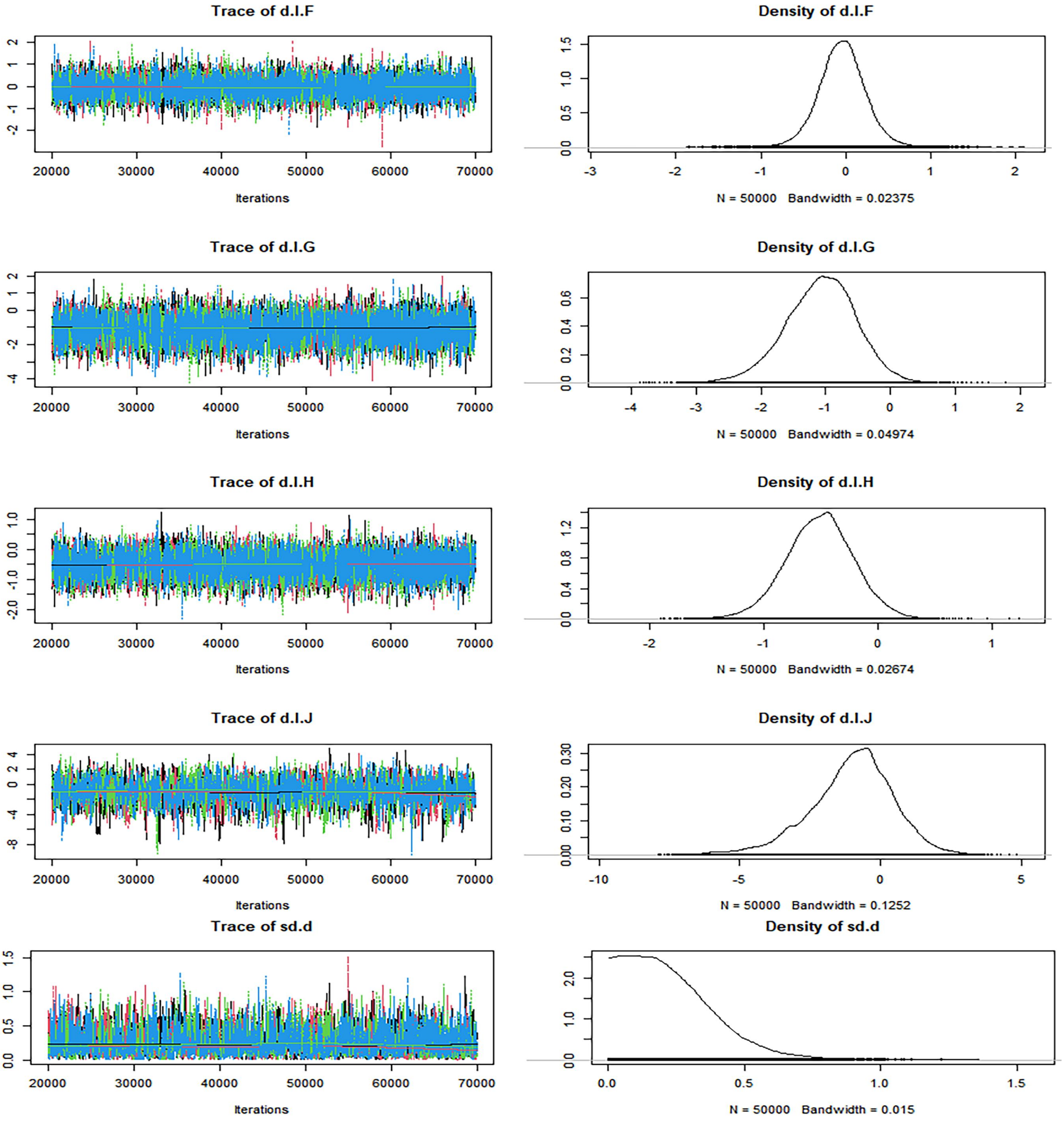
Trace of d.I.F
Iterations
Density of d.I.F
N = 50000 Bandwidth = 0.02375
Trace of d.I.G
Iterations
Density of d.I.G
N = 50000 Bandwidth = 0.04974
Trace of d.I.H
Iterations
Density of d.I.H
N = 50000 Bandwidth = 0.02674
Trace of d.I.J
Iterations
Density of d.I.J
N = 50000 Bandwidth = 0.1252
Trace of sd.d
Iterations
Density of sd.d
N = 50000 Bandwidth = 0.015

(4)

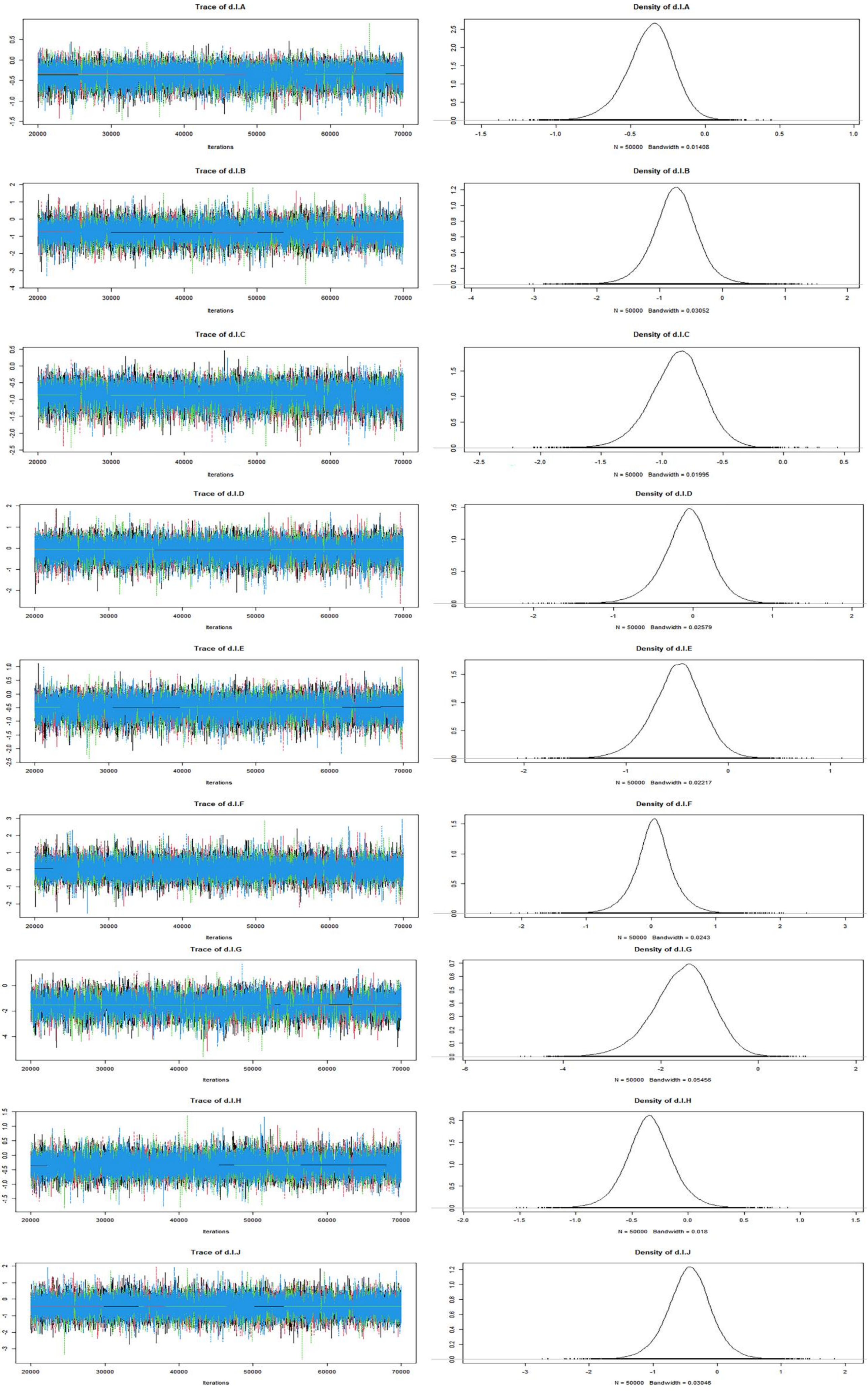
Trace of d.I.A
Iterations
Density of d.I.A
N = 50000 Bandwidth = 0.01408
Trace of d.I.B
Iterations
Density of d.I.B
N = 50000 Bandwidth = 0.03052
Trace of d.I.C
Iterations
Density of d.I.C
N = 50000 Bandwidth = 0.01995
Trace of d.I.D
Iterations
Density of d.I.D
N = 50000 Bandwidth = 0.02579
Trace of d.I.E
Iterations
Density of d.I.E
N = 50000 Bandwidth = 0.02217
Trace of d.I.F
Iterations
Density of d.I.F
N = 50000 Bandwidth = 0.0243
Trace of d.I.G
Iterations
Density of d.I.G
N = 50000 Bandwidth = 0.05456
Trace of d.I.H
Iterations
Density of d.I.H
N = 50000 Bandwidth = 0.018
Trace of d.I.J
Iterations
Density of d.I.J
N = 50000 Bandwidth = 0.03046

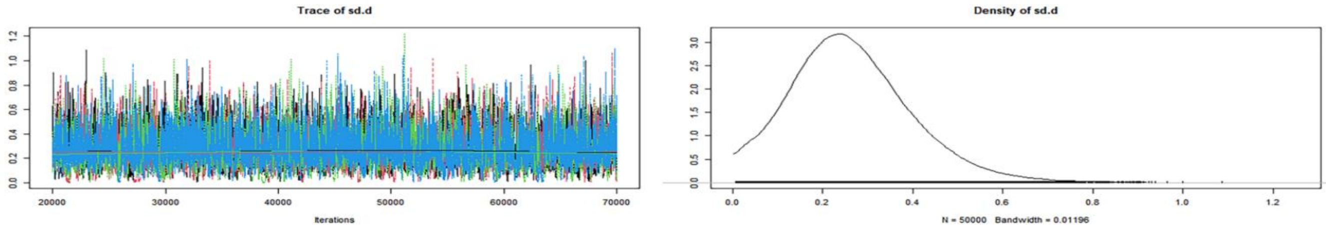
Trace of sd.d
Iterations
Density of sd.d
N = 50000 Bandwidth = 0.01196

(5)

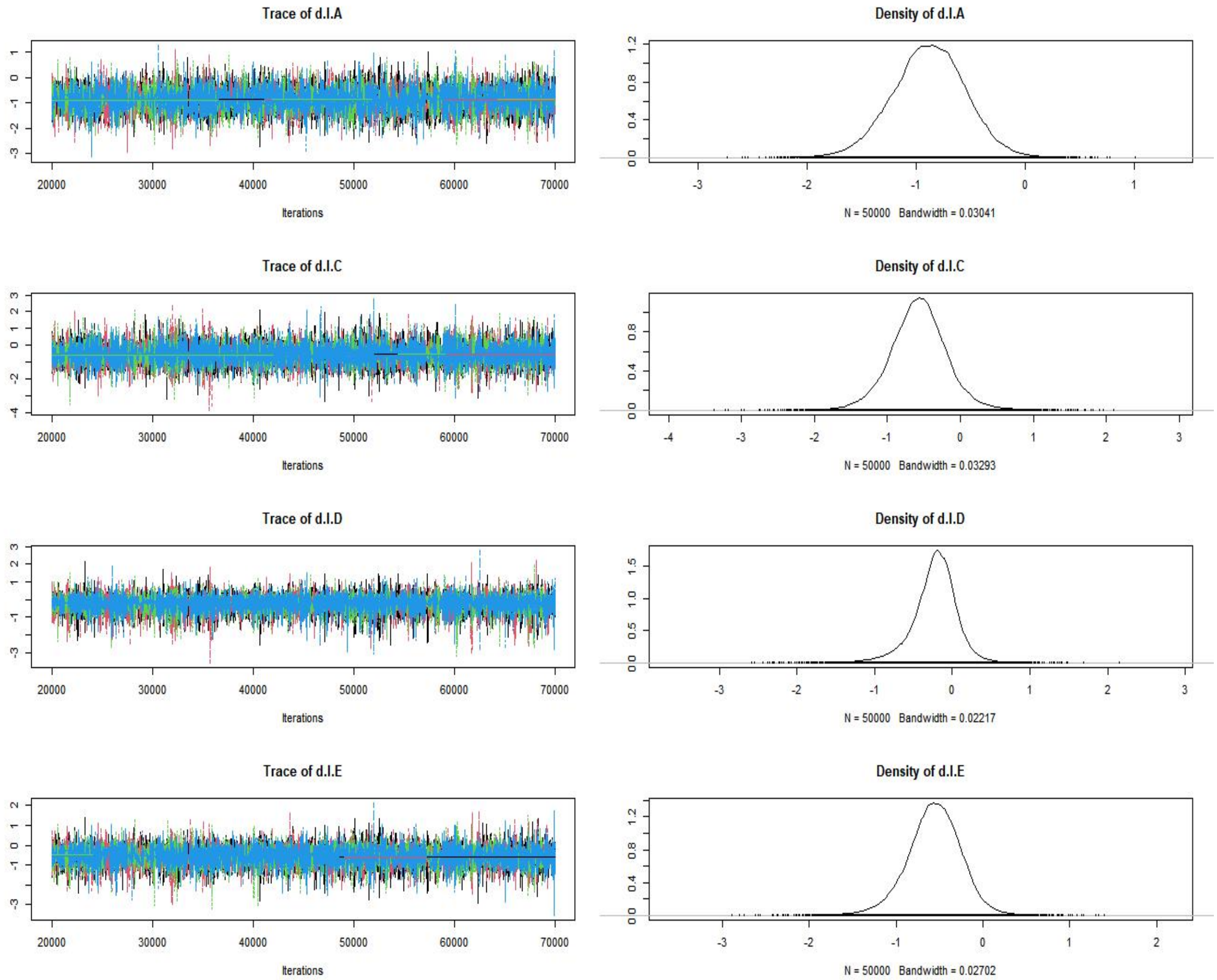
Trace of d.I.A
Iterations
Density of d.I.A
N = 50000 Bandwidth = 0.03041
Trace of d.I.C
Iterations
Density of d.I.C
N = 50000 Bandwidth = 0.03293
Trace of d.I.D
Iterations
Density of d.I.D
N = 50000 Bandwidth = 0.02217
Trace of d.I.E
Iterations
Density of d.I.E
N = 50000 Bandwidth = 0.02702

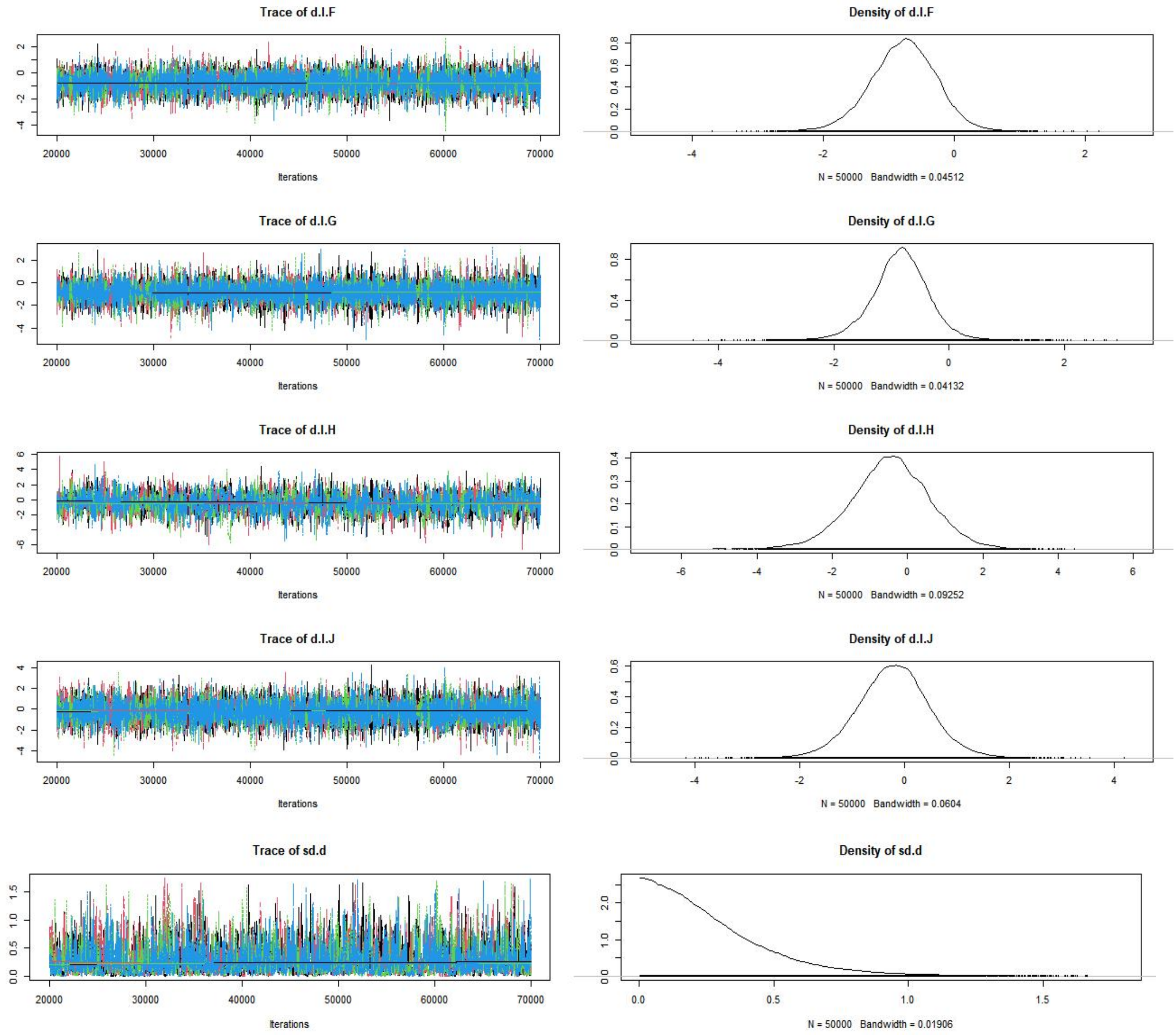
Trace of d.I.F
Iterations
Density of d.I.F
N = 50000 Bandwidth = 0.04512
Trace of d.I.G
Iterations
Density of d.I.G
N = 50000 Bandwidth = 0.04132
Trace of d.I.H
Iterations
Density of d.I.H
N = 50000 Bandwidth = 0.09252
Trace of d.I.J
Iterations
Density of d.I.J
N = 50000 Bandwidth = 0.0604
Trace of sd.d
Iterations
Density of sd.d
N = 50000 Bandwidth = 0.01906

(6)

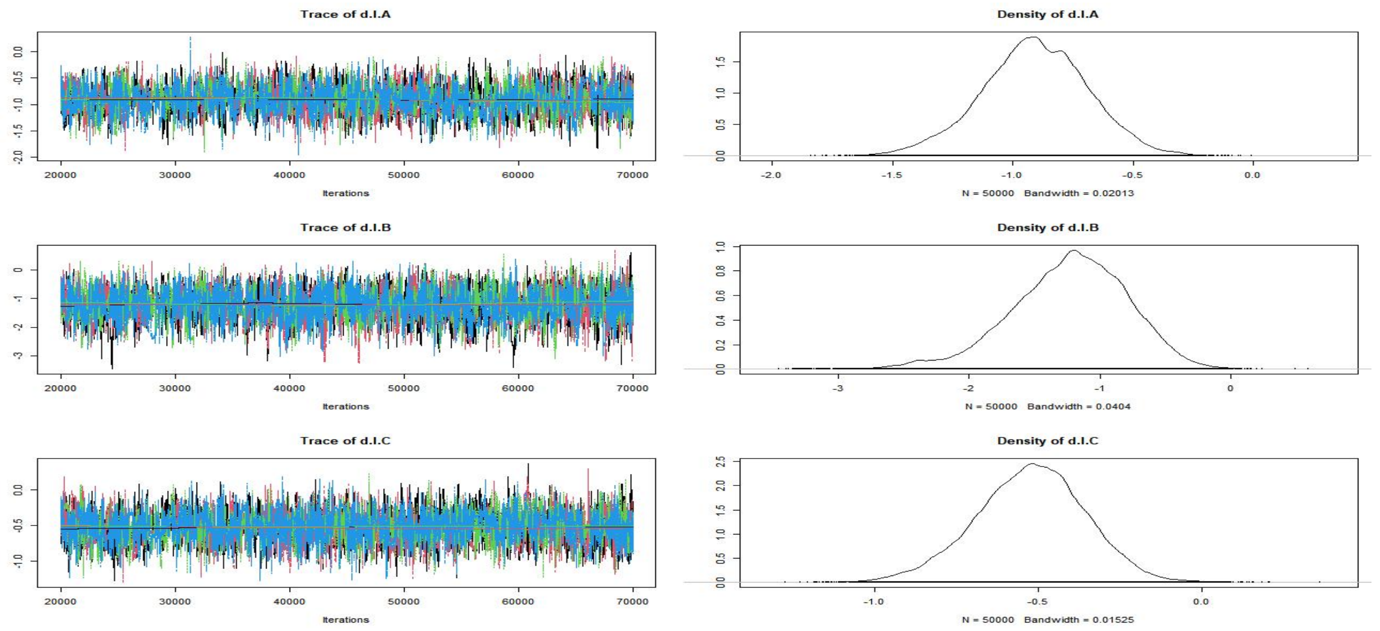
Trace of d.I.A
Iterations
Density of d.I.A
N = 50000 Bandwidth = 0.02013
Trace of d.I.B
Iterations
Density of d.I.B
N = 50000 Bandwidth = 0.0404
Trace of d.I.C
Iterations
Density of d.I.C
N = 50000 Bandwidth = 0.01525

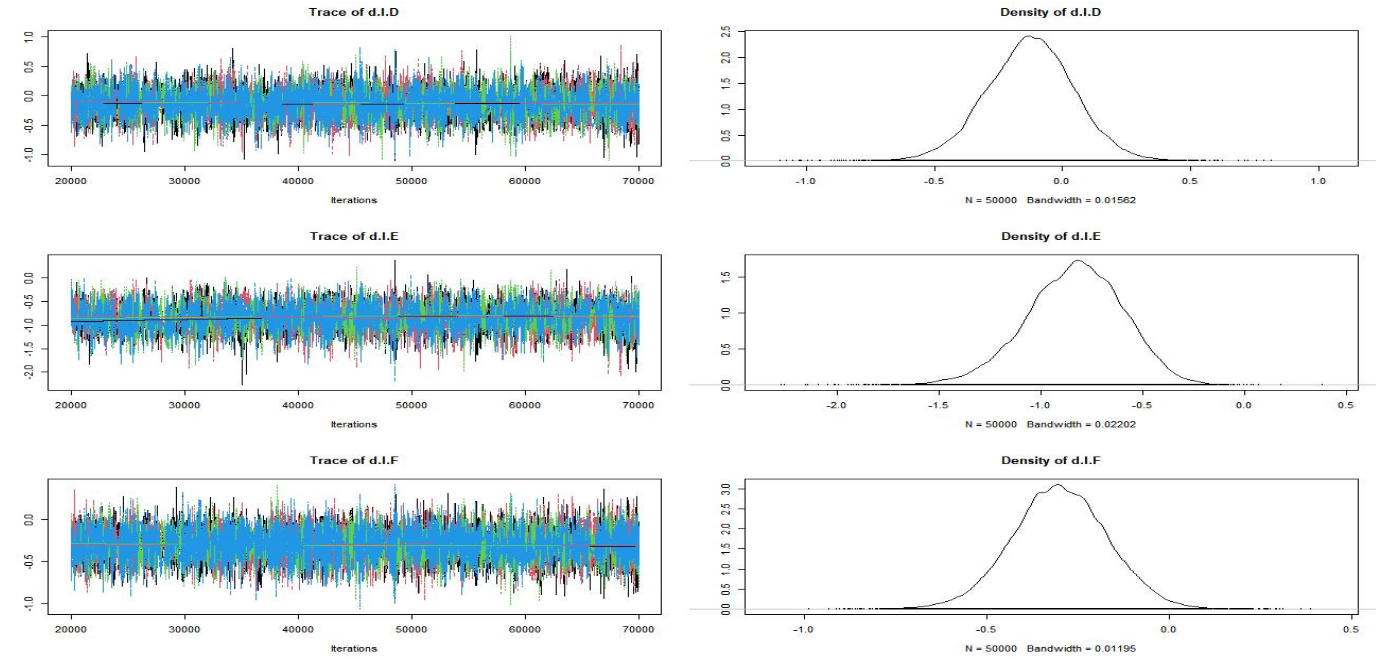
Trace of d.I.D
Iterations
Density of d.I.D
N = 50000 Bandwidth = 0.01562
Trace of d.I.E
Iterations
Density of d.I.E
N = 50000 Bandwidth = 0.02202
Trace of d.I.F
Iterations
Density of d.I.F
N = 50000 Bandwidth = 0.01195

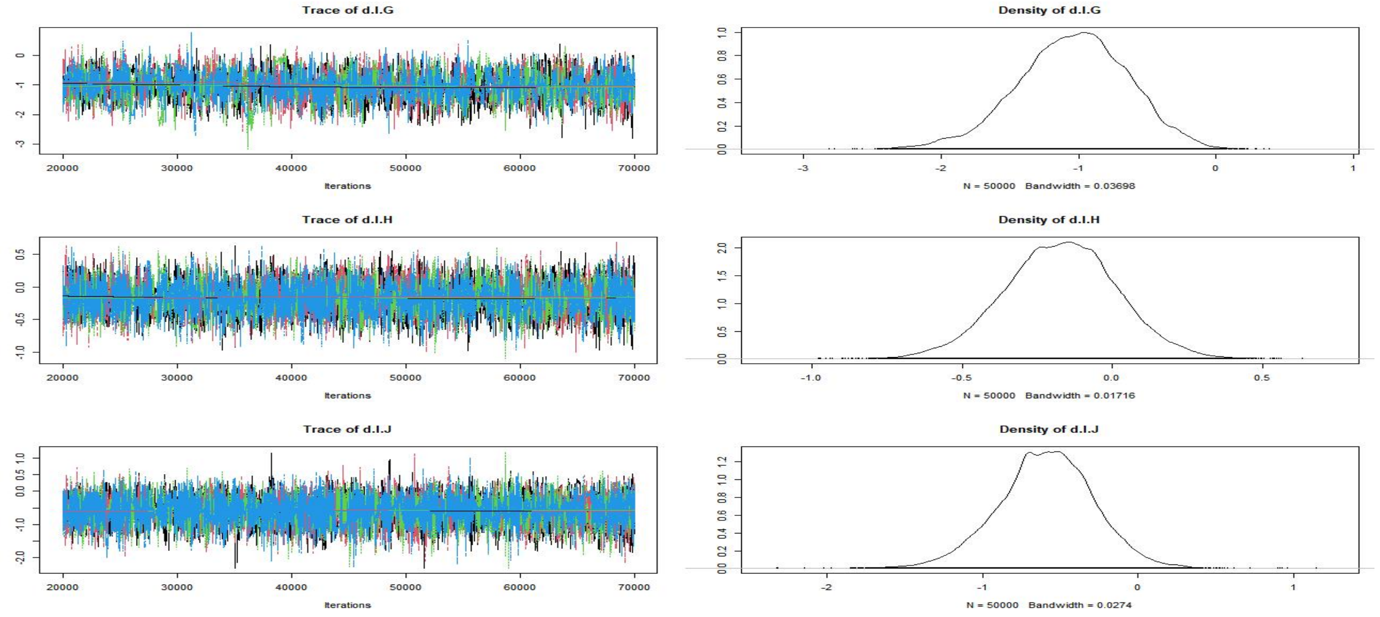
Trace of d.I.G
Iterations
Density of d.I.G
N = 50000 Bandwidth = 0.03698
Trace of d.I.H
Iterations
Density of d.I.H
N = 50000 Bandwidth = 0.01716
Trace of d.I.J
Iterations
Density of d.I.J
N = 50000 Bandwidth = 0.0274

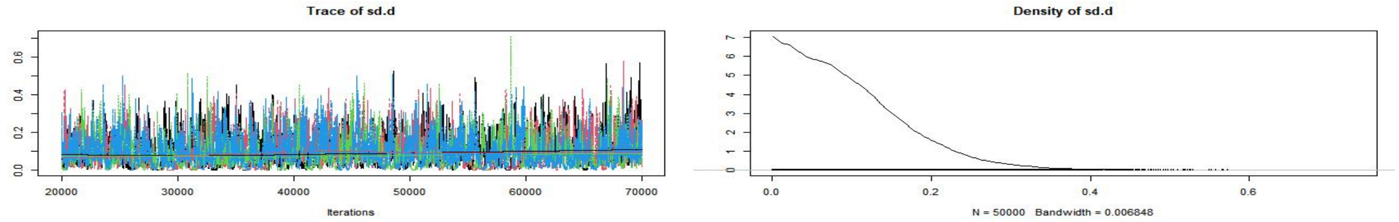

(7)

Figure S7, The specific trajectory maps and density maps. (1), Clinical effectiveness. (2), The improvement rate of KPS score. (3), Incidence of leukopenia rate. (4), Incidence of thrombocytopenia. (5), Incidence of nausea and vomiting. (6), Incidence of liver function damage. (7), Incidence of peripheral neurotoxicity. “A”, ADI+SOX. “B”, SFI+SOX. “C”, SQFZI+SOX. “E”, KAI+SOX. “F”, KLTI+SOX. “G”, HCSI+SOX. “H”, XAPI+SOX. “I”, SOX.“J”, HQI+SOX. ADI, AiDi injections. SFI, Shenfu injections. SQFZI, Shenqifuzheng injections. KAI, Kangai injections. KLTI, Kanglaitei injections. HCSI, Huachansu injections. XAPI, Xiaoaiping injections. SOX, SOX chemotherapy regimens, HQI, Huangqi injections.

Supplement S8

The Brooks-Gelman-Rubin diagnostic plots.

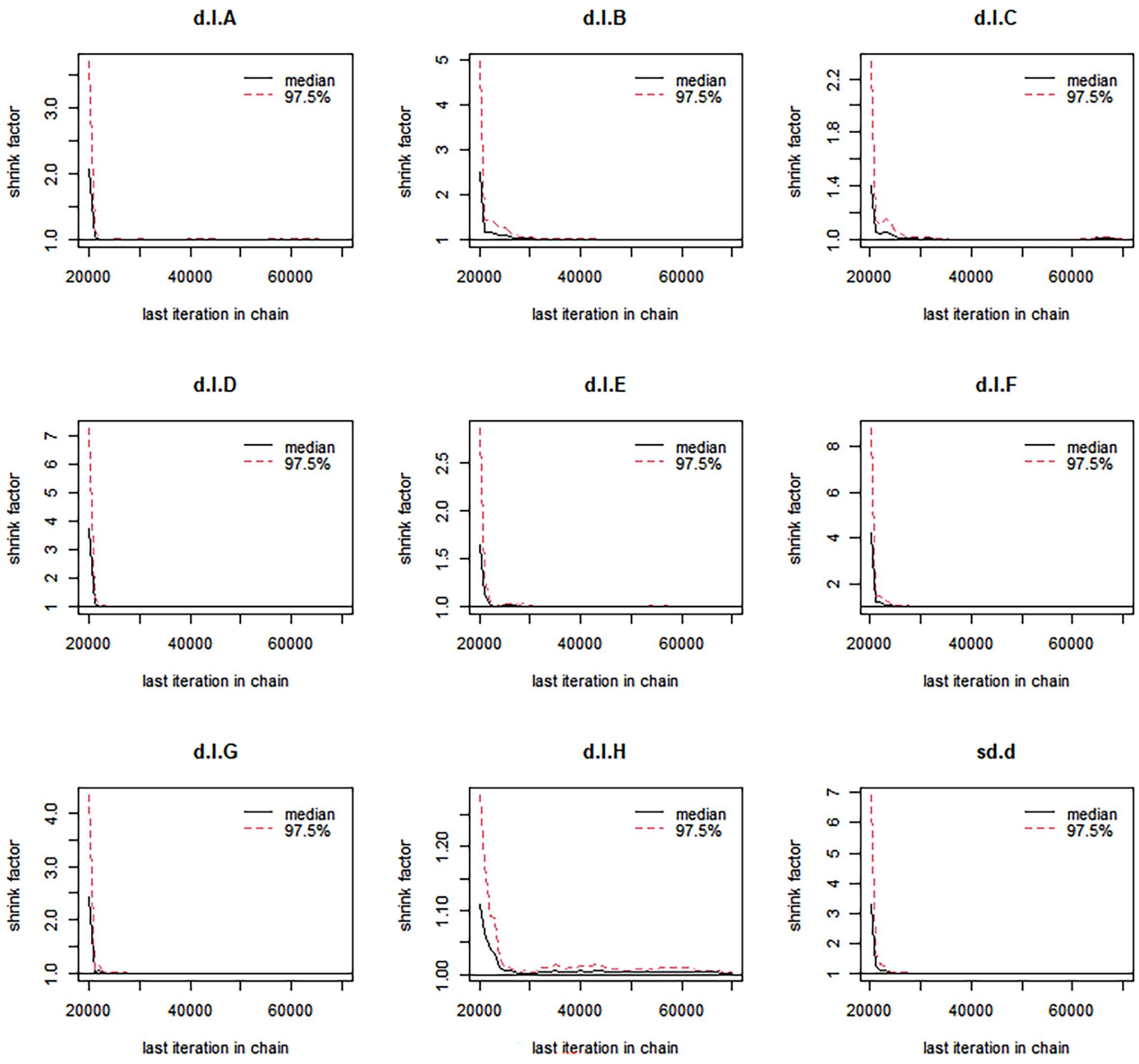

(1)

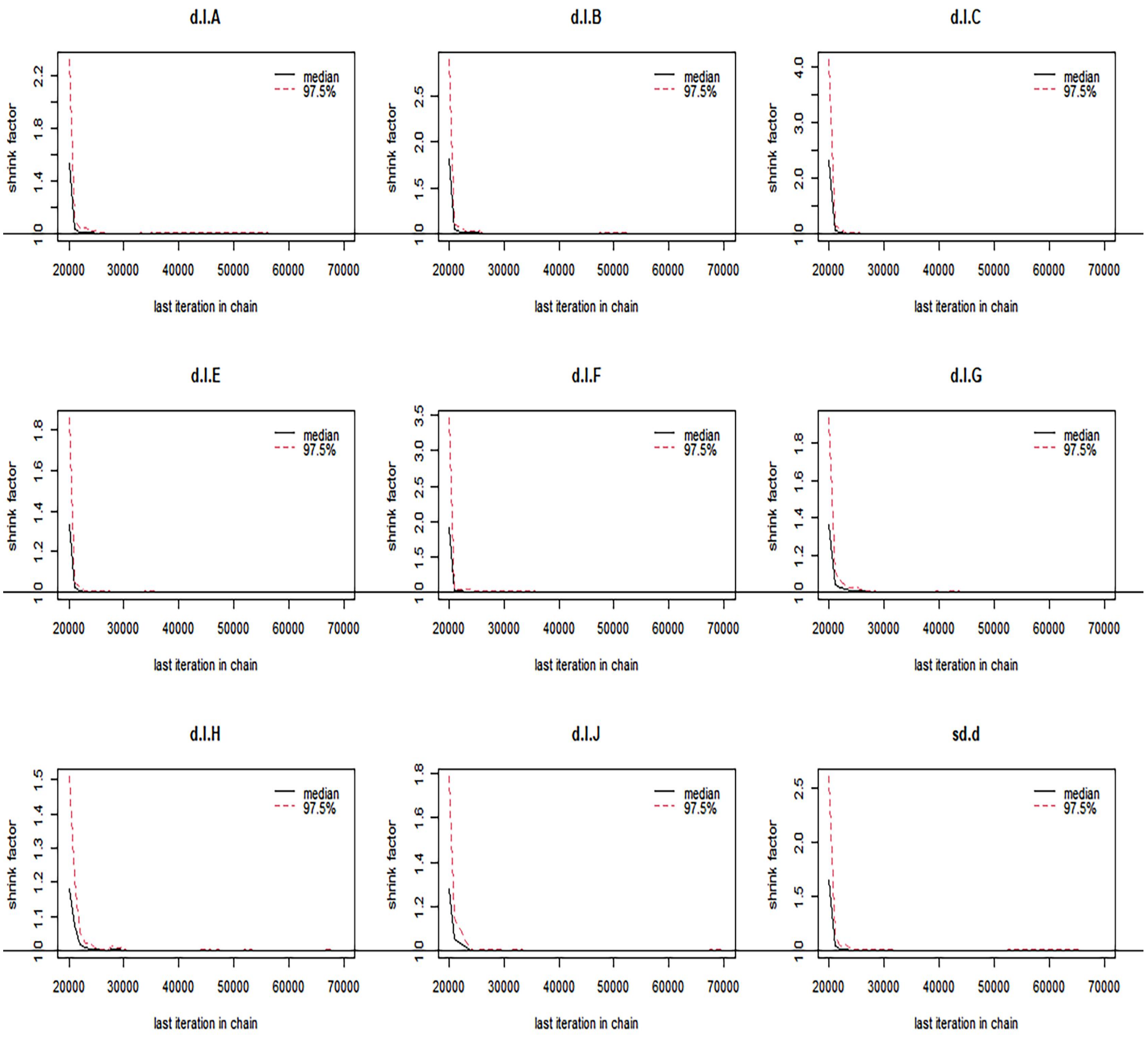
d.l.A
d.l.B
d.l.C
d.l.E
d.l.F
d.l.G
d.l.H
d.l.J
sd.d
shrink factor
last iteration in chain
median
97.5%

(2)

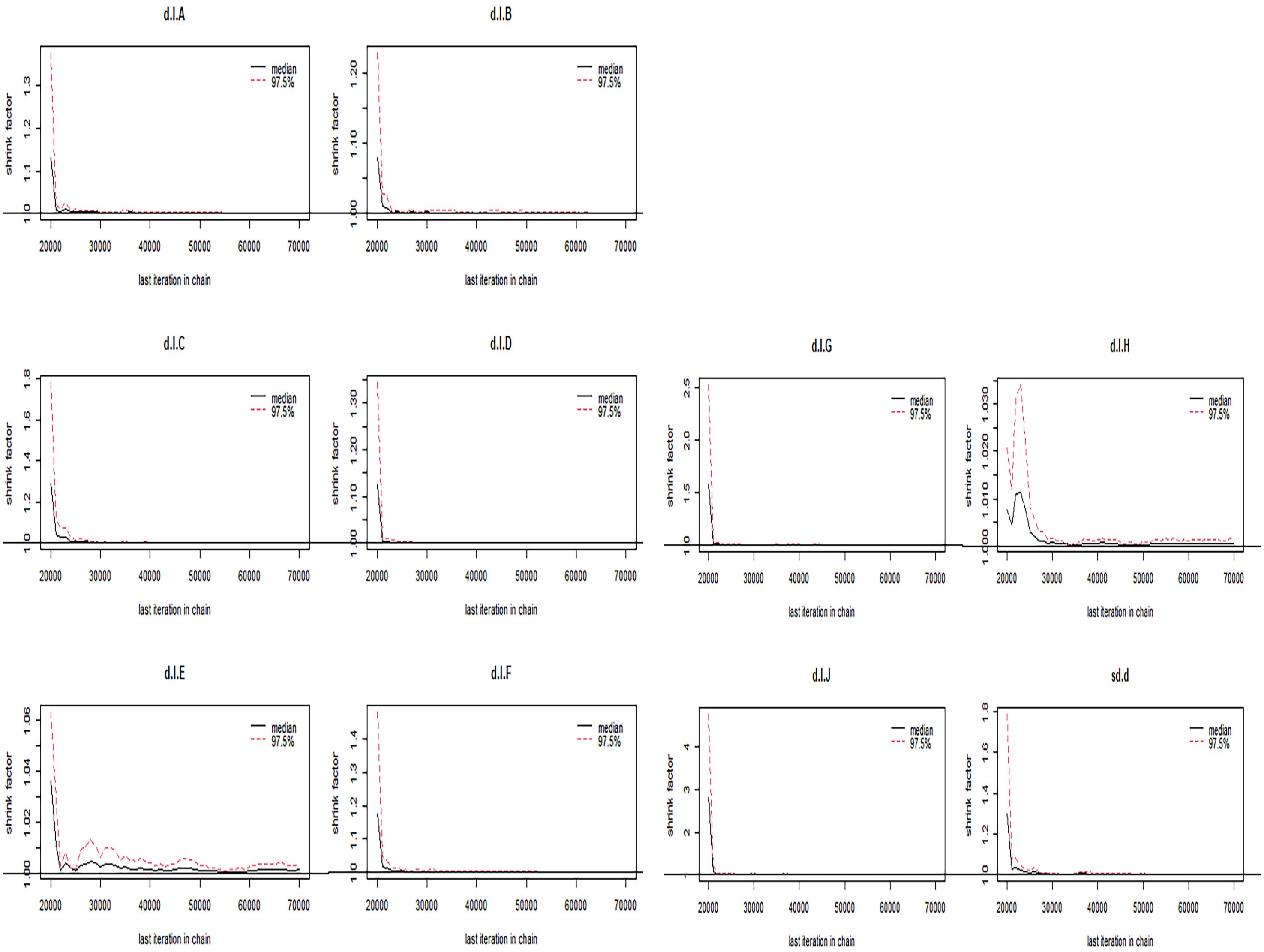
d.I.A
d.I.B
d.I.C
d.I.D
d.I.G
d.I.H
d.I.E
d.I.F
d.I.J
sd.d
shrink factor
last iteration in chain
median
97.5%

(3)

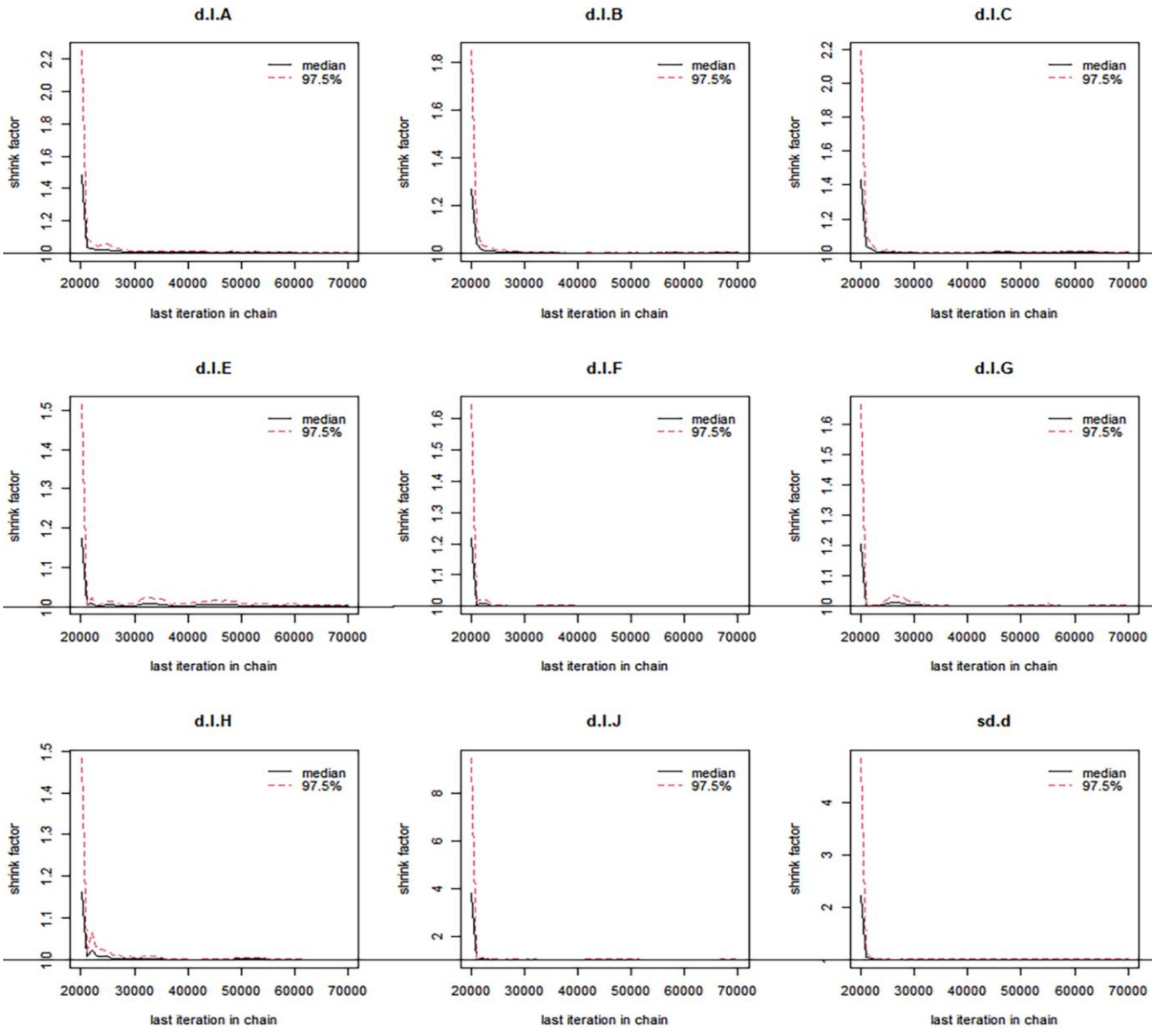
d.l.A
d.l.B
d.l.C
d.l.E
d.l.F
d.l.G
d.l.H
d.l.J
sd.d
median
97.5%
shrink factor
last iteration in chain
20000 30000 40000 50000 60000 70000

(4)

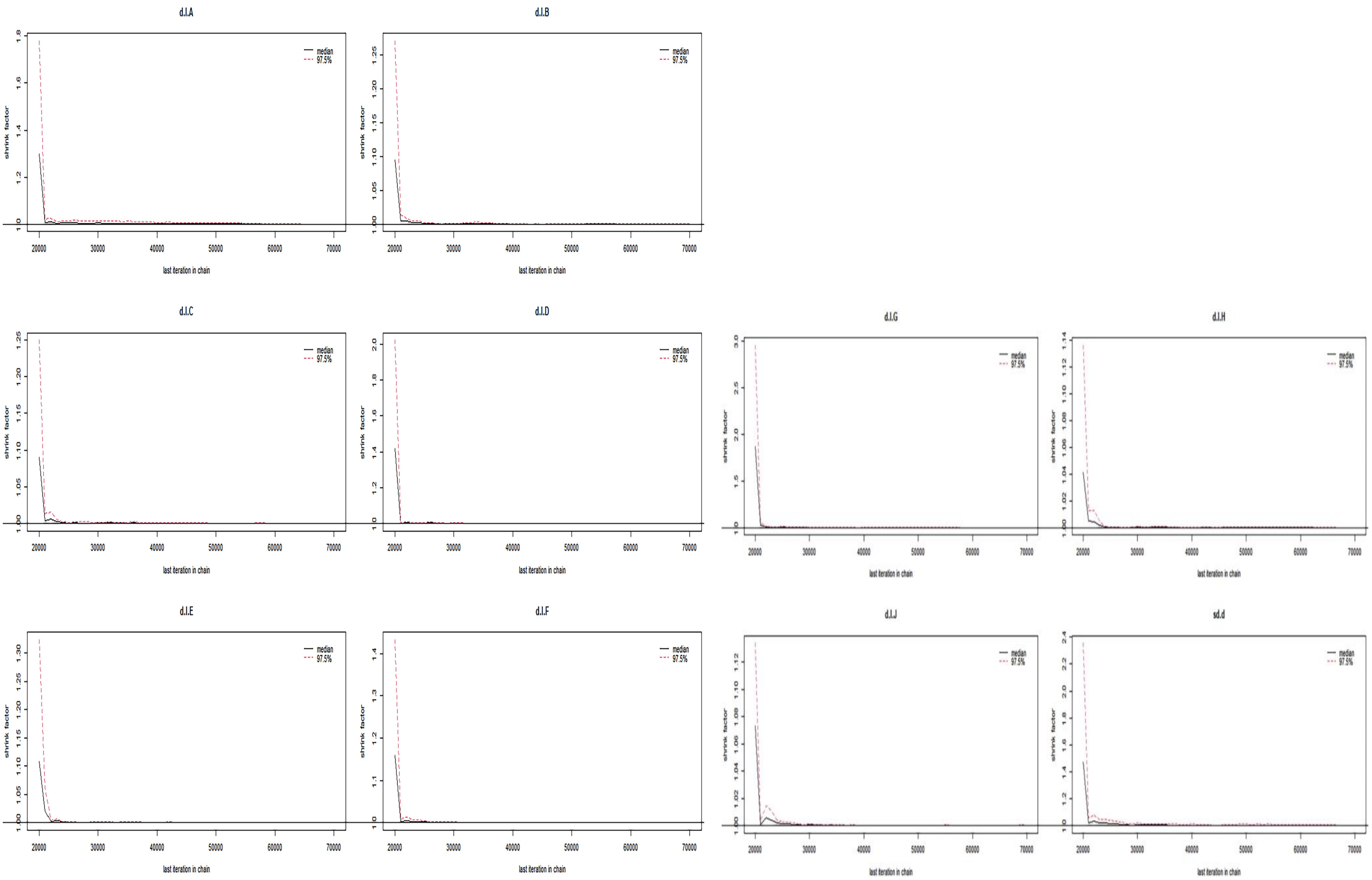

(5)

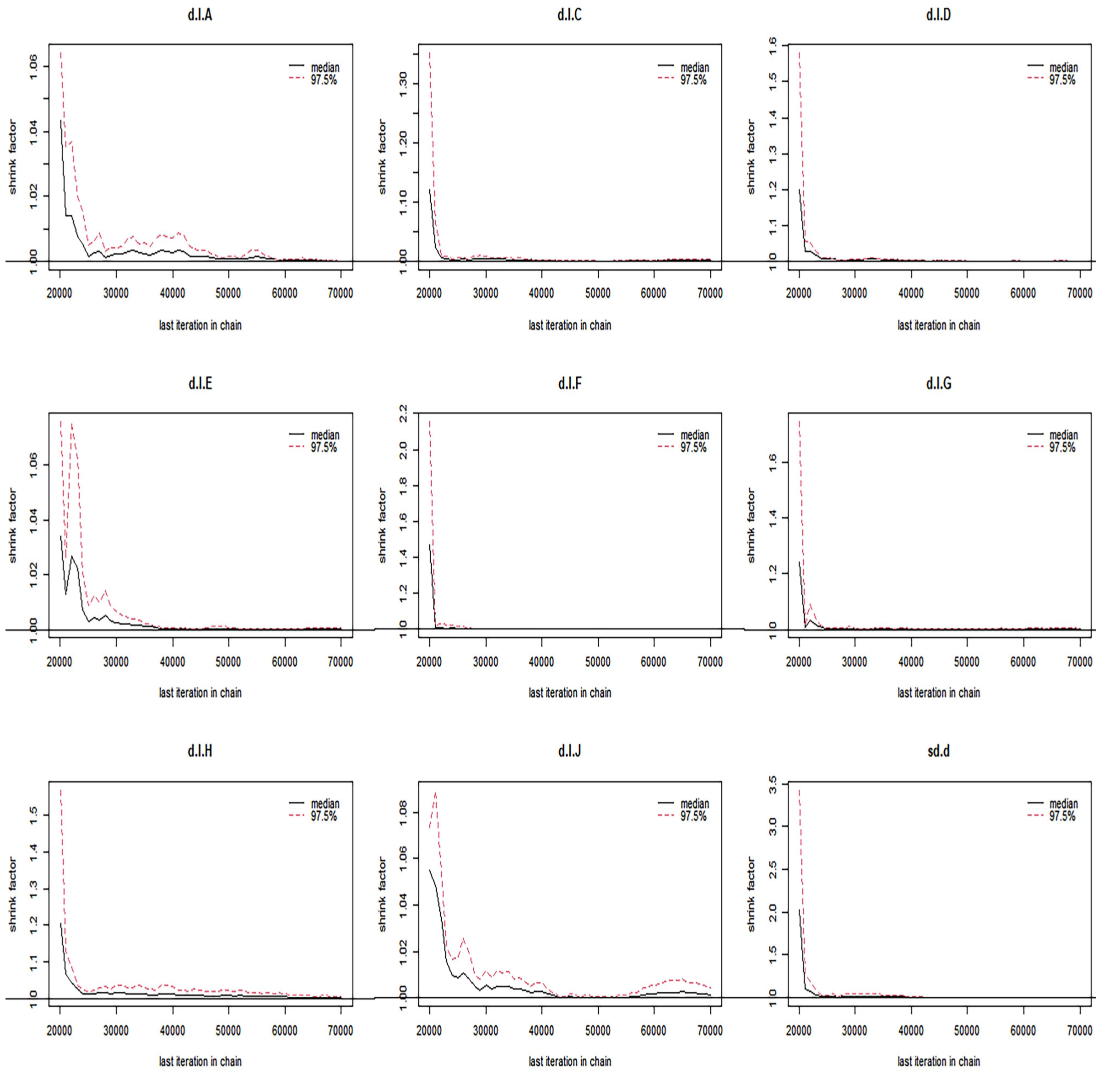
d.I.A
d.I.C
d.I.D
d.I.E
d.I.F
d.I.G
d.I.H
d.I.J
sd.d
median
97.5%
shrink factor
last iteration in chain

(6)

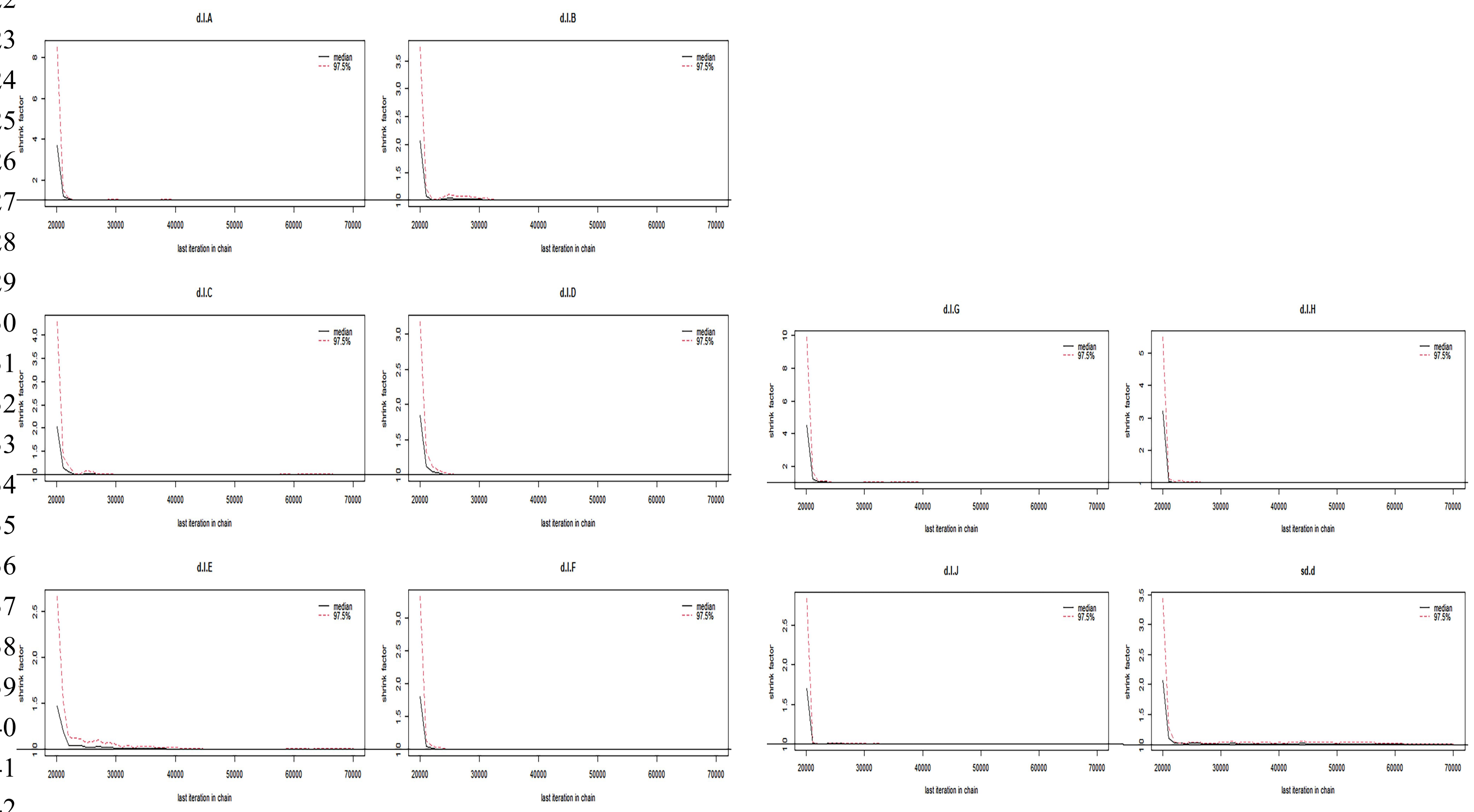

(7)

Figure S8, Brooks-Gelman-Rubin diagnostic maps. (1), Clinical effectiveness. (2), The improvement rate of KPS score. (3), Incidence of leukopenia rate. (4), Incidence of thrombocytopenia. (5), Incidence of nausea and vomiting. (6), Incidence of liver function damage. (7), Incidence of peripheral neurotoxicity. “A”, ADI+SOX. “B”, SFI+SOX. “C”, SQFZI+SOX. “E”, KAI+SOX. “F”, KLTI+SOX. “G”, HCSI+SOX. “H”, XAPI+SOX. “I”, SOX.“J”, HQI+SOX. ADI, AiDi injections. SFI, Shenfu injections. SQFZI, Shenqifuzheng injections. KAI, Kangai injections. KLTI, Kanglaitei injections. HCSI, Huachansu injections. XAPI, Xiaoaiping injections. SOX, SOX chemotherapy regimens, HQI, Huangqi injections.

Supplement S9

Specific characteristics of studies reporting survival data

Id	Treatments	Survival indicators	Intervention (month)	Control (month)
Dou SS 2021	G versus.I	mPFS	36	26
Chang ZG 2021	D versus.I	mPFS	8.4	5.5
Zhao HB 2020	A versus.I	TTP	11.1	4.62
		MST	22.42	11.14
Li RQ 2020	A versus.I	Actual Average Survival	15.58	12.37
Chen XT 2020	C versus.I	3-Year Average Survival Rate	19.8%	46.2%
		3-Year Average Mortality Rate	79.6%	52.8%
Xu JL 2017	A versus.I	TTP	10.45	8.29
		MST	27.36	20.65
Tang XF 2017	C versus.I	TTP	11.76	8.27
		MST	16.38	12.03
Hu Q 2017	E versus.I	MST	22	15
Yin Q 2015	C versus.I	mPFS	5.6	5.1
Xiong L 2015	H versus.I	mPFS	8.41	6.01
		MST	10.36	8.62
Jiang J 2015	C versus.I	1-Year Average Survival Rate	46.7%	26.7%
		3-Year Average Survival Rate	33.3%	20%
		5-Year Average Survival Rate	20%	6.7%
Li YQ 2013	A versus.I	MST	11.2	10.7
		TTP	4.6	4.3
		1-Year Average Survival Rate	40.1%	37.5%
Zhang WH 2012	C versus.I	MST	12	8
Liu HZ 2012	A versus.I	1-Year PFS rate	82.1%	75%
		2-Year PFS rate	67.9%	39.3%
Fan CM 2011	A versus.I	MST	13.5	10.7

Median Survival Time: MST, Median Progression-Free Survival: mPFS, Time to Progression:TTP. A, AiDi injections (ADI), B, Shenfu injections (SFI), C, Shenqifuzheng injections (SQFZI), D, Fufangkushen injections (FFKSI), E, Kangai injections (KAI), F, Kanglaitei injections (KLTI), G, Huachansu injections (HCSI), H, Xiaoaiping injections (XAPI), I, SOX chemotherapy regimens, J, Huangqi injections (HQI).

Supplement S10

The Clinical effectiveness standard of the 51 studies included in the NMA analysis.

Study ID	Clinical effectiveness criteria
Xue TL 2023	RECIST
Han B 2022	RECIST
Yang QW 2021	RECIST
Yang Q 2021	RECIST
Si LL 2021	RECIST
Ruan XJ 2021	RECIST
Dou SS 2021	RECIST
Chang ZG 2021	WHO
Zhao HB 2020	WHO
Zhang MM 2020	-
Shi ZW 2020	RECIST
Qian YM 2020	-
Li RQ 2020	RECIST
ChenYX 2020	WHO
Chen XT 2020	WHO
Wang P 2019	RECIST
Xu RQ 2019	WHO
Song B 2019	WHO
Wu Y 2019	WHO
Gao CL 2019	-
Dong L 2019	WHO
Wang R 2018	WHO

Rao JJ 2018	WHO
Liu HT 2018	-
Gao NN 2018	RECIST
Xu JL 2017	RECIST
Tang XF 2017	WHO
Shen G 2017	RECIST
Pang YP 2017	RECIST
Liu W 2017	RECIST
Li CH 2017	-
Hu Q 2017	RECIST
Yan LF 2016	-
Xu SG 2016	-
Xie JF 2016	WHO
Ma YK 2017	WHO
Gao M 2017	WHO
Yin Q 2015	-
Yao XJ 2015	RECIST
Xiong L 2015	WHO
Ma YJ 2015	WHO
Jiang J 2015	WHO
Yang ZY 2014	RECIST
Xie YG 2014	RECIST
Wang J 2013	RECIST
Sun GZ 2013	WHO
Li YQ 2013	RECIST

Zhang WH 2012	WHO
Ruan XJ 2012	RECIST
Liu HZ 2012	-
Fan CM 2011	WHO

RECIST: Response Evaluation Criteria in Solid Tumours. WHO: World Health Organization criteria
-: Due to the lack of reporting on clinical effectiveness, there is no established standard for clinical effectiveness.
